# StereoSiTE: a framework to spatially and quantitatively profile the cellular neighborhood organized iTME

**DOI:** 10.1093/gigascience/giae078

**Published:** 2024-10-25

**Authors:** Xing Liu, Chi Qu, Chuandong Liu, Na Zhu, Huaqiang Huang, Fei Teng, Caili Huang, Bingying Luo, Xuanzhu Liu, Min Xie, Feng Xi, Mei Li, Liang Wu, Yuxiang Li, Ao Chen, Xun Xu, Sha Liao, Jiajun Zhang

**Affiliations:** BGI Research, Chongqing 401329, PR China; BGI Research, Shenzhen 518083, PR China; BGI Research, Chongqing 401329, PR China; BGI Research, Shenzhen 518083, PR China; JFL-BGI STOmics Center, Jinfeng Laboratory, Chongqing 401329, China; BGI Research, Chongqing 401329, PR China; BGI Research, Shenzhen 518083, PR China; BGI Research, Shenzhen 518083, PR China; BGI Research, Chongqing 401329, PR China; BGI Research, Shenzhen 518083, PR China; BGI Research, Shenzhen 518083, PR China; BGI Research, Shenzhen 518083, PR China; BGI Research, Chongqing 401329, PR China; BGI Research, Shenzhen 518083, PR China; BGI Research, Chongqing 401329, PR China; BGI Research, Shenzhen 518083, PR China; JFL-BGI STOmics Center, Jinfeng Laboratory, Chongqing 401329, China; BGI Research, Chongqing 401329, PR China; BGI Research, Shenzhen 518083, PR China; JFL-BGI STOmics Center, Jinfeng Laboratory, Chongqing 401329, China; BGI Research, Shenzhen 518083, PR China; BGI Research, Chongqing 401329, PR China; BGI Research, Shenzhen 518083, PR China; JFL-BGI STOmics Center, Jinfeng Laboratory, Chongqing 401329, China; BGI Research, Shenzhen 518083, PR China; BGI Research, Chongqing 401329, PR China; BGI Research, Shenzhen 518083, PR China; JFL-BGI STOmics Center, Jinfeng Laboratory, Chongqing 401329, China; BGI Research, Chongqing 401329, PR China; BGI Research, Shenzhen 518083, PR China; JFL-BGI STOmics Center, Jinfeng Laboratory, Chongqing 401329, China; BGI Research, Chongqing 401329, PR China; BGI Research, Shenzhen 518083, PR China; JFL-BGI STOmics Center, Jinfeng Laboratory, Chongqing 401329, China; BGI Research, Chongqing 401329, PR China; BGI Research, Shenzhen 518083, PR China; JFL-BGI STOmics Center, Jinfeng Laboratory, Chongqing 401329, China

**Keywords:** spatial transcriptomics, cellular neighborhood, spatial cell interaction intensity, immune tummor microenvironment, treatment response

## Abstract

**Background:**

Spatial transcriptome (ST) technologies are emerging as powerful tools for studying tumor biology. However, existing tools for analyzing ST data are limited, as they mainly rely on algorithms developed for single-cell RNA sequencing data and do not fully utilize the spatial information. While some algorithms have been developed for ST data, they are often designed for specific tasks, lacking a comprehensive analytical framework for leveraging spatial information.

**Results:**

In this study, we present StereoSiTE, an analytical framework that combines open-source bioinformatics tools with custom algorithms to accurately infer the functional spatial cell interaction intensity (SCII) within the cellular neighborhood (CN) of interest. We applied StereoSiTE to decode ST datasets from xenograft models and found that the CN efficiently distinguished different cellular contexts, while the SCII analysis provided more precise insights into intercellular interactions by incorporating spatial information. By applying StereoSiTE to multiple samples, we successfully identified a CN region dominated by neutrophils, suggesting their potential role in remodeling the immune tumor microenvironment (iTME) after treatment. Moreover, the SCII analysis within the CN region revealed neutrophil-mediated communication, supported by pathway enrichment, transcription factor regulon activities, and protein–protein interactions.

**Conclusions:**

StereoSiTE represents a promising framework for unraveling the mechanisms underlying treatment response within the iTME by leveraging CN-based tissue domain identification and SCII-inferred spatial intercellular interactions. The software is designed to be scalable, modular, and user-friendly, making it accessible to a wide range of researchers.

## Introduction

The immune tumor microenvironment (iTME) consists of tumor cells, immune cells, and noncellular components of the extracellular matrix. The rearrangement of these components within the iTME plays a crucial role in tumor formation, progression, response to therapy, and the development of multidrug resistance. Understanding the dynamic crosstalk between tumor cells and their neighboring microenvironment is essential for unraveling the underlying mechanisms of tumor growth and metastasis [1]. The advent of advanced spatial transcriptomic (ST) technology provides a unique opportunity to study the cellular and molecular mechanisms of tumors by observing the spatial distribution of the transcriptome [2]. The spatially coordinated expression profiles offer insights into the landscape of the iTME and the spatial intercellular communication occurring in pathogenesis-associated iTME regions. However, accurately identifying the precise factors contributing to pathogenesis within the iTME and quantitatively inferring spatial intercellular communication by effectively utilizing spatial information remain significant challenges.

Most researchers rely on tools developed for analyzing single-cell RNA sequencing (scRNAseq) datasets or underutilizing the spatial information available. However, these approaches have limitations. First, most analyses are conducted on the entire dataset of a single sample, disregarding the heterogeneity of the iTME that can be distinguished within ST datasets. By identifying regions consisting of specific iTME components and exploring the molecular mechanisms within these regions, more precise and sensitive results can be achieved. Additionally, existing open-source tools tailored for analyzing scRNAseq datasets do not account for the spatial proximity of cells, such as CellPhoneDB [3] and CellChat v1 [4]. Newly developed tools for inferring spatial communication in ST datasets, like Spatalk [5] and Giotto [6], primarily rely on cell graphs generated using K-nearest neighbors (KNN) or Delaunay triangulation, without incorporating the physical distances between cells. Furthermore, these tools may lack the computational efficiency required to analyze large-scale datasets comprising millions of cells.

Here, we present StereoSiTE, an analytical framework designed to explore the landscape of the iTME and infer spatial cell interactions. StereoSiTE leverages the concept of cellular neighborhoods (CNs) to identify distinct regions within the ST datasets. By dividing the datasets into adjacent windows of a specific size, these windows are clustered into CNs based on their cellular composition, revealing the architectural organization of the iTME. To infer spatial cell interaction intensity within specific CN regions, StereoSiTE constructs a cell graph using both cell coordinates and the expression of ligand–receptor (LR) gene pairs. This enables the quantification of intercellular communication at a spatial level. With a scalable and modular Python package, StereoSiTE offers flexibility for users to substitute or combine different modules based on their needs, including the deconvolution method, tissue domain division method, and the use of the LR database. For the LR database, StereoSiTE provides integration with CellChatDB, which assigns each LR with an interaction distance associated classification. Additionally, users can utilize LR databases collected by other tools like CellPhoneDB [3], CellTalkDB [7], CellDialog [8], CellComNet [9], and CellGiQ [10]. The framework is optimized for efficient computational performance through matrix calculations and is capable of processing ST datasets with millions of cells. To assess the performance of StereoSiTE, we evaluated its capabilities using stereo-seq datasets from xenograft models treated with immunoagonists. The results revealed the landscape of CNs, with 1 CN located at the border of the necrotic region found to be enriched with neutrophils. Through spatial cell interaction intensity (SCII) analysis within this CN region, active communications were identified between neutrophils and nonimmune cells, providing valuable insights into the cellular and molecular mechanisms underlying the tumor’s response to treatment.

## Results

### StereoSiTE: an analytical framework to spatially and quantitatively profile spatial intercellular communications within iTME organized CN regions

**Figure 1:**
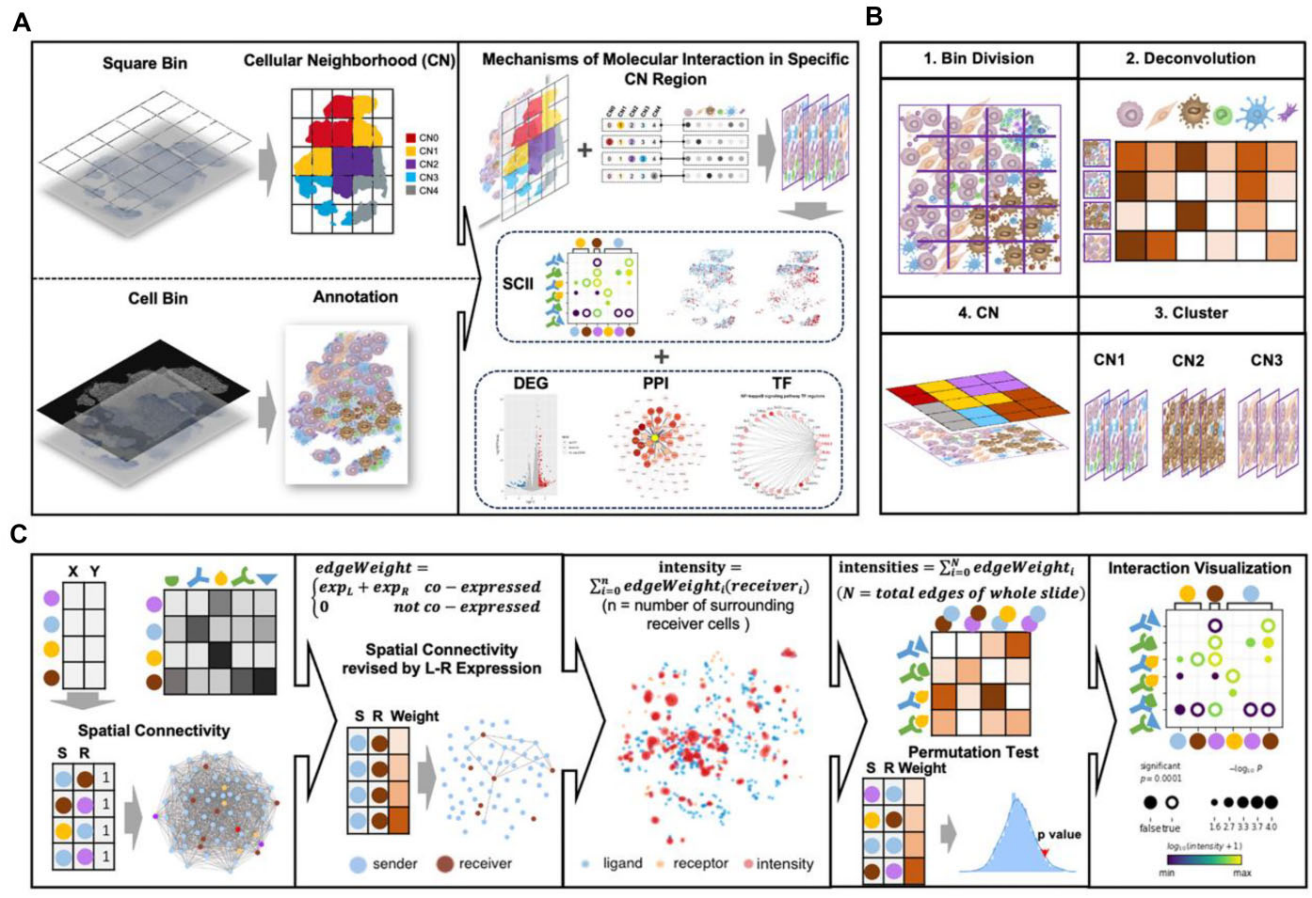
Schematic diagram of the StereoSiTE workflow. (A) Overview of StereoSiTE. (B) Conceptual diagram of CN: 1. The spatial gene expression matrix is converted into a binned data matrix. 2. Resolve the cellular composition of each bin through deconvolution. 3. Cluster bins into distinct CNs based on their cellular composition. 4. Spatial distribution of CNs. (C) Principle of SCII: first, construct the cell graph by connecting cells within a defined radius threshold. Second, assign weights to the connected edges based on coexpression levels of LR genes, and remove the edges without LR coexpression. Third, calculate the local interaction intensity between each sender cell with their surrounding receiver cells and show their spatial distribution. Then, calculate the interaction intensity between any 2 cell types of the entire slide, use a permutation test to build a null distribution by shuffling the cell type labels, and compute the *P* value. Finally, the spatial cell interaction intensities and *P* values are visualized using bubble plots indicating interaction intensity (by color) and *P* value (by size).

In this framework (Fig. 1A), we first performed cell-type deconvolution for squared bin data (each bin contains more than 1 cell) and cell-type annotation for cell bin data by integrating a published single-cell sequencing dataset. For these tasks, we employed Cell2location [11], which demonstrated superior performance compared to other deconvolution methods (Celloscope [12], GraphST [13], POLARIS [14]) on both STARmap [15] datasets (Supplementary Fig. S1A) and the stereo-seq dataset of liver cancer (Supplementary Fig. S1B). However, it is worth noting that results generated by other deconvolution methods can also be utilized for subsequent CN and SCII analyses.

Next, we explored the landscape of distinct CNs based on cellular composition (Fig. 1B), which is vital for understanding the organizational structure of the iTME. This analysis was performed on squared bin data using the deconvolution method (cell2location), with scRNAseq data serving as a reference [16]. The squared bins with similar composition were clustered together using the Leiden algorithm. Each resulting cluster represents a distinct type of iTME, characterized by a specific cellular composition. To identify CNs of interest for further functional analysis, we integrate a matrix that encompasses both CNs and cell types (CTs). We then employed Tensor decomposition to unravel the underlying module matrix.

Moving forward, we employed our self-developed SCII method to analyze spatially resolved intercellular communication within specific CN regions. This analysis provided insights into key molecular activities associated with specific iTME architectures. SCII quantitatively defines the intensity of interactive communication between cells by taking into account their spatial proximity and the expression levels of corresponding LR genes. For improved accuracy, we recommend utilizing spatially resolved data at a single-cell resolution, which can be obtained through sequencing-based methods such as stereo-seq [17], Seq-Scope [18], or imaging-based methods like MERFISH [19], seqFISH [20], and STARMap [21]. In our research, we employed the StereoCell [22] cell segmentation algorithm, which utilizes a deep neural network approach, to generate single-cell masks based on nuclear staining images. These masks were then combined with the spatial expression matrix to derive the single-cell resolution spatial expression profile. Cell-type annotation was performed using cell2location, following the protocol mentioned previously.

With the annotated single-cell resolve data, we constructed a cell graph by connecting cells within a defined radius threshold. To improve the accuracy of the graph, we assigned weights to the connected edges based on the coexpression levels of LR genes. Edges where the end nodes, representing sender and receiver cells, had no expression of ligands or receptors were filtered out. Subsequently, we calculated the local interaction intensity between each sender cell and its surrounding receiver cells by summing up the weights of the connected edges. The overall interaction intensity of the entire slide was determined by summing up the weights of all connected edges. To evaluate the significance of these interactions, we generated a null distribution through a permutation test by shuffling the cell-type annotation labels. By comparing the observed interaction intensity with the null distribution, we could assess the statistical significance of the interactions (Fig. 1C).

To confirm inferred cell–cell communications, both upstream and downstream signaling activities of the CN regions of interest have been comprehensively analyzed. StereoSiTE (RRID: SCR_025,236) incorporates analysis modules of differentially expressed genes (DEGs), the protein–protein interaction (PPI) network, and the transcriptional regulatory factor (TF) network to provide an end-to-end solution for molecular mechanism exploration within specific iTME units.

### CN accurately segments tissue domains

**Figure 2:**
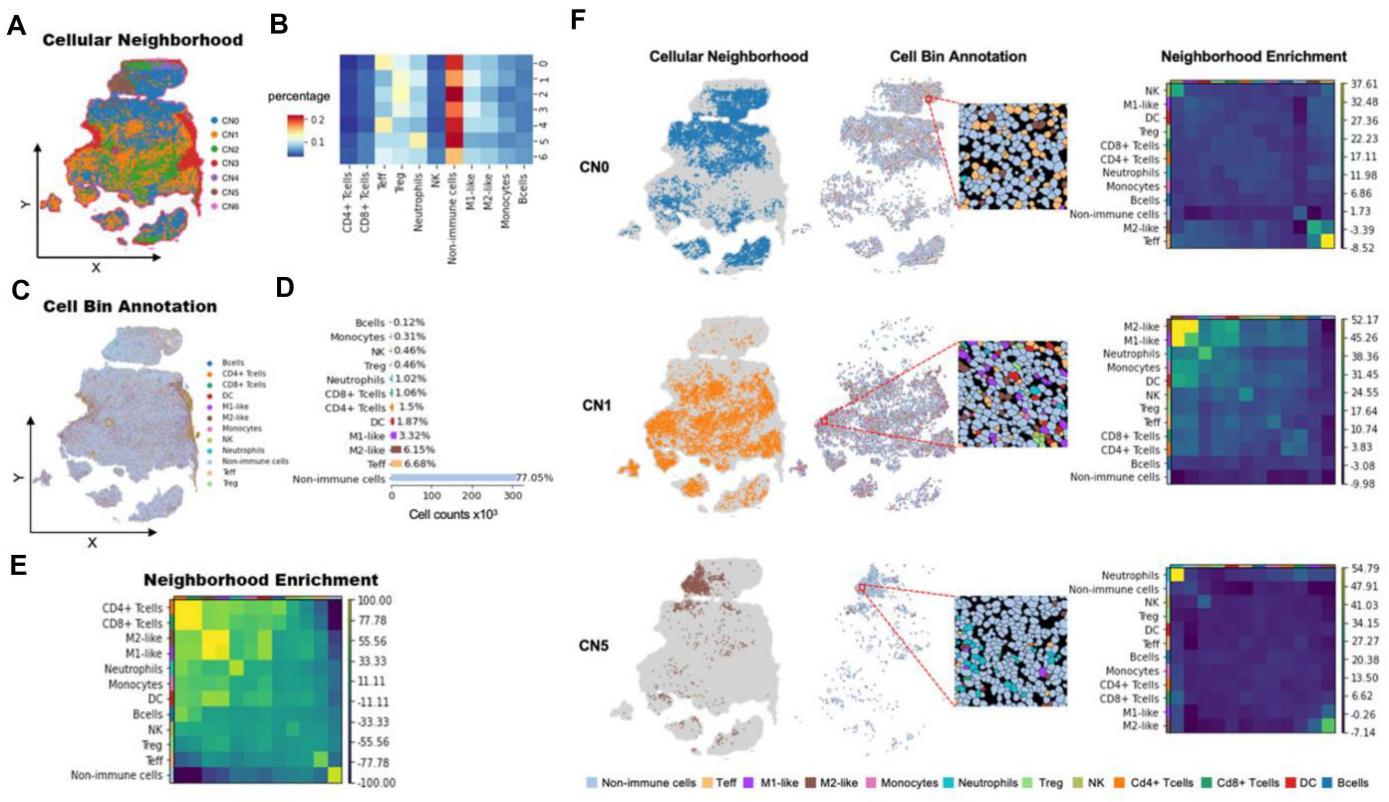
Performance of CN in decoding iTME. (A) The spatial distribution of CNs. (B) Heatmap illustrating the cellular composition of each CN region, with different colors indicating different cell types. (C) Spatial distribution of annotated cells at the single-cell resolution. Each spot represents a single cell, and the color denotes its cell type. (D) Bar plot displaying the count and percentage of each cell type within the analyzed sample. The x-axis represents the cell number, while the y-axis represents the cell types. (E) Heatmap demonstrating the neighborhood enrichment between any 2 cell types, with a radius threshold set to 50 µm. This analysis reveals the spatial aggregation patterns between different cell types. (F) Spatial distribution of representative CNs (CN0, CN1, CN5) (left). The corresponding cell distribution of representative CNs (CN0, CN1, CN5) is shown in the middle, with a zoomed-in region for clearer observation. On the right, neighborhood enrichment between any 2 cell types within CN0, CN1, and CN5 is displayed. DC: dendritic cell; M1-like: M1-type macrophage; M2-like: M2-type macrophage; NK: natural killer cell; Teff: T effector; Treg: T regulatory.

To validate the relevance of studying the iTME using the concept of CN, we applied this framework to analyze representative stereo-seq data obtained from a xenograft model’s cancer tissue. Initially, we constructed a binned data matrix using a bin size of 100 (equivalent to 50 × 50 µm) and utilized the deconvolution method to determine the cell-type composition of each bin. By clustering the bins based on their cellular composition, we identified 7 distinct CN clusters (Fig. 2A). Each cluster exhibited a unique composition of cell types (Fig. 2B). To gain further insight into the microenvironment of each CN, we aligned the annotated data at single-cell resolution onto the clustered CNs, guided by their spatial coordinates. Fig. 2C and D show the spatial distribution of annotated cells and the proportion of each cell type in the sample, respectively.

To verify the ability of CN to identify significant spatial features, we conducted a quantification of the spatial aggregation between different annotated cell types using neighborhood enrichment analysis [23]. Our analysis revealed the presence of various cell types, such as T cells, macrophages, and nonimmune cells, showing aggregation patterns throughout the entire slide, as depicted in Fig. 2E. However, determining the critical cell types that distinguish this particular sample from others based solely on this observation proved challenging. To gain a clearer understanding of the spatial characteristics within individual CN regions, we performed enrichment analysis within each CN. Remarkably, each CN exhibited a dominant cell type with pronounced spatial aggregation, as illustrated in Fig. 2F. Notably, in the CN0 region, the enrichment of T effector (Teff) cells was particularly pronounced compared to other regions. Although this enrichment was observed throughout the entire slide, it was not easily distinguishable due to interference from signals of other cell types. This discrepancy may be attributed to the presence of a small portion of Teff cells and the predominance of other immune cell types on the entire slide, while Teff cells were dominantly aggregated in CN0. In the CN1 region, the neighborhood enrichment of M1-like and M2-like cells was substantially stronger compared to other cell types. This finding indicates a clear dominance of macrophage aggregation within CN1, which is more evident compared to the enrichment observed in the entire slide. Additionally, in the CN5 region, we observed significant neighborhood enrichment of neutrophils, indicating a spatial preference. In summary, our analysis demonstrates that CN analysis enables the division of tissue regions into distinct domains, each associated with a specific iTME. This approach helps to identify spatially dominant cell types and their aggregation patterns, which may be overlooked if the CN of interest is not identified prior to conducting molecular analysis. By leveraging CN analysis, we mitigate the risk of overlooking important candidate cell types for further in-depth investigation.

To evaluate the performance of our CN method, we compared it with other published tissue domain division methods, namely BANKSY [24] and Giotto HMRF [6], using the benchmark dataset STARmap. The results of this evaluation demonstrate that our CN method consistently performs on par with BANSKY and surpasses Giotto HMRF (Supplementary Fig. S1C, D). Furthermore, based on BANKSY’s comparative analysis with various other methods such as GraphST [13], SpaGCN [25], SpiceMix [26], STAGATE [27], and BayesSpace [28] in its original study, we assert that our CN method is competent for tissue domain division. Moreover, in comparison to BANKSY, CN exhibited the capability to handle large-scale datasets with reduced processing time (Supplementary Fig. S1E), indicating enhanced user-friendliness.

In the following section, we will introduce the key module of StereoSiTE, called SCII, which is used to decode the spatial intercellular interaction in specific iTME regions.

### SCII: inferring spatial cell–cell communications

**Figure 3:**
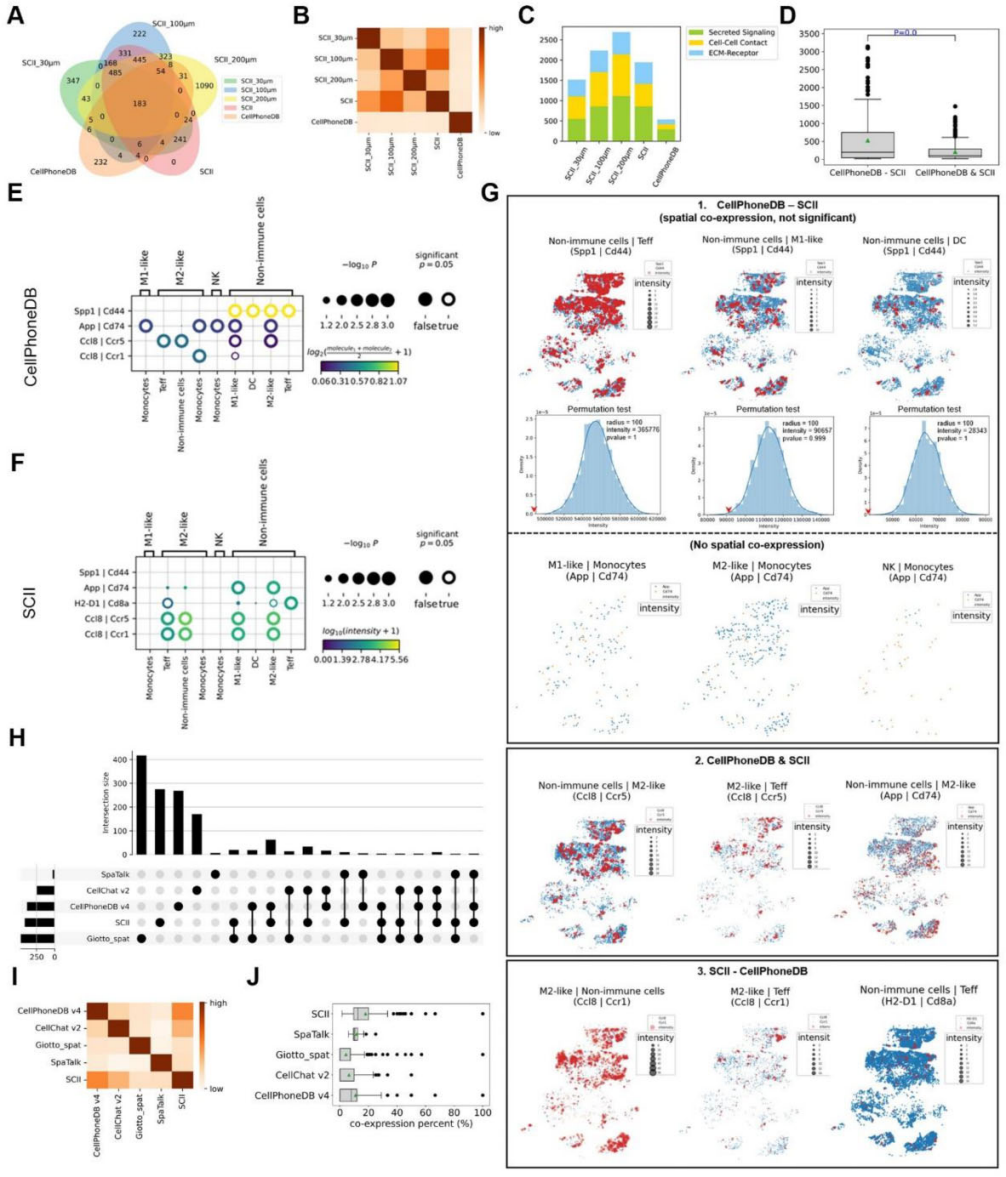
Superior performance of SCII compared to other interaction inference methods. (A) Venn diagram illustrates the intersection and variance in inferred interactions from SCII with radius thresholds of all = 30 µm (SCII_30 µm), all = 100 µm (SCII_100 µm), all = 200 µm (SCII_200 µm), “Secreted Signaling = 100, ECM Receptor = 100, Cell–Cell Contact = 30” (SCII), and CellPhoneDB. (B) The Jaccard index between any 2 interaction results. (C) Stacked bar plots show the composition of different interaction types. Green indicates secreted signaling, yellow indicates cell–cell contact, and blue indicates ECM receptor. (D) For each communication, the sender cell is connected with its *N* surrounding receiver cells to construct a cell graph using KNN. Then, the median distances of all connected cell pairs were calculated and shown by a boxplot. “CellPhoneDB—SCII” indicates interactions inferred by CellPhoneDB alone, and “CellPhoneDB & SCII” indicates interactions inferred by both CellPhoneDB and SCII. An approximate 2-sided *P* -value from a Wilcoxon rank-sum test is shown. The green triangle indicates the mean value. (E) Representative results inferred by CellPhoneDB. The color of the bubble indicates the average expression of the LR pairs, while the size indicates confidence. A confidence *P* value less than .05 is indicated by a circle, representing a significant interaction. (F) Representative results inferred by SCII. The color of the bubble indicates the intensity strength, while the size represents the same as D. (G) Spatial distribution of communications between sender and receiver cells mediated by a specific ligand–receptor. 1. CellPhoneDB—SCII: Interactions detected by CellPhoneDB alone but not by SCII can be divided into 2 categories: spatial coexpression but not significant and no spatial coexpression. The spatial distribution of these interactions is shown, and the corresponding null distribution of permutation tests is displayed at the bottom. Red arrows indicate the actual intensity values. For interactions with no spatial coexpression, a permutation test was not able to be conducted. 2. CellPhoneDB & SCII: Spatial distribution of interactions inferred by both CellPhoneDB and SCII. 3. SCII—CellPhoneDB: Spatial distribution of interactions inferred by SCII alone. Blue spots indicate sender cells expressing the ligand gene, orange spots indicate receiver cells expressing the receptor gene, and red spots indicate local interaction intensity. (H) The upset plot displays the intersection of interactions inferred by SCII and other methods (CellPhoneDB v4, CellChat v2, Giotto, SpaTalk). (I) The Jaccard index indicates the correlation between interactions inferred by different methods. (J) Performance comparison of SCII with other methods. The boxplots display the coexpression percentage of interactions inferred by different methods. The green triangle indicates the mean value.

To evaluate the impact of cellular distance on cell–cell communications, we conducted a validation procedure comparing the results from our SCII method at various distance thresholds. To establish recommended parameters, we also considered the variability among different open-source LR databases and recommend using LR datasets from CellChatDB. These datasets classify each LR based on its associated interaction distance, such as secreted signaling, extracellular matrix (ECM) receptor, and cell–cell contact, which is crucial for SCII analysis. Fig.   3A shows the count and overlap of cell–cell communications inferred by SCII at different distance thresholds (30 µm, 100 µm, and 200 µm) for all types of LR pairs, as well as the different distance thresholds for different LR types (30 µm for cell–cell contact LR pairs and 200 µm for secreted signaling and ECM receptor pairs). We also compared the results from SCII with those inferred by CellPhoneDB, which disregards the distance between cells. Fig. 3B illustrates the association between the communication results from these 2 methods, while Fig. 3C shows the proportion of different types of interactions.

It is important to note that there was limited overlap between the LR interactions inferred by SCII and CellPhoneDB, regardless of the distance thresholds used. We also calculated the median cell distance of communications inferred by CellPhoneDB alone and by both CellPhoneDB and SCII (Fig. 3C). The distances between neighboring cell pairs involved in communications exclusively predicted by CellPhoneDB were significantly longer than others (Fig. 3D), indicating a limitation in the physically reachable interactions predicted by CellPhoneDB. In other words, CellPhoneDB inferred many false-positive interactions, which can be avoided by incorporating a distance threshold in the SCII analysis. The false-positive interactions inferred by CellPhoneDB are listed in Supplementary Table S1.

Figure 3E shows representative LR pairs identified by CellPhoneDB, while Fig. 3F shows those identified by SCII. In Fig. 3G, we mapped the intensity of these inferred cell–cell communications *in situ*, which include 3 categories: (i) interactions inferred by CellPhoneDB alone, (ii) interactions inferred by both CellPhoneDB and SCII, and (iii) interactions inferred by SCII alone. Interactions inferred by CellPhoneDB alone can be further clustered into 2 categories in Fig. 3G and Supplementary Fig. S1F, G. The first category includes interactions that exhibit spatial coexpression between ligands and receptors but lack significance in spatial proximity with high *P* values. An example of this category is the interaction mediated by Spp1-Cd44 between nonimmune cells with Teff cells, M1-like cells, and dendritic cells (DCs). The second category includes interactions that show no spatial coexpression between ligands and receptors. An example of this category is interactions mediated by App-Cd74 between M1-like cells, M2-like cells, and natural killer (NK) cells with monocytes. Interactions induced by App and Cd74 require direct contact between sender and receiver cells. This demonstrates that the introduction of a distance threshold could prevent false positives caused by interactions between unreachable cells. Figure 3G also shows communications inferred by both CellPhoneDB and SCII, such as the interactions between nonimmune cells, M2-like cells, and Teff cells mediated by Ccl8-Ccr5 and App-Cd74. Additionally, communications exclusively inferred by SCII were observed between M2-like cells, nonimmune cells, and Teff cells mediated by Ccl8-Ccr1 and H2-D1-Cd8a, with a strong intensity of interaction and significance. This further supports the superior accuracy of SCII over methods that do not consider spatial information.

In addition, we conducted a comprehensive comparison of SCII with several other measurement methods that consider spatial information. These methods include CellChat v2 [29], Giotto with its spatCellCellcom function [6], and SpaTalk [5]. Due to the limitations in computational efficiency and the incompetence to process large datasets, we extracted a small portion of the demo data containing 11,214 cells for the analysis (Supplementary Fig. S1H). To ensure standardized LR databases across different methods, we selected LR pairs that exist in both CellPhoneDB and CellChatDB [4]. Additionally, as some methods cannot handle LR pairs with complexes, we filtered out protein complexes, resulting in 441 LR pairs for further analysis. The intersections of interactions inferred by different methods are displayed in Fig. 3H and Supplementary Fig. S1I, showing low overlap between the methods. However, the interactions inferred by SCII had a higher overlap with CellPhoneDB and CellChatDB compared to Giotto and SpaTalk. We reasoned that a higher coexpression percentage to the inferred interactions between sender and receiver cells indicates more reliable inference. In this regard, interactions inferred by SCII exhibited the highest coexpression level among the different methods, indicating the superior performance of SCII in measuring spatial cell–cell communication.

### Profiling tumor microenvironment using spatial transcriptomics 

**Figure 4:**
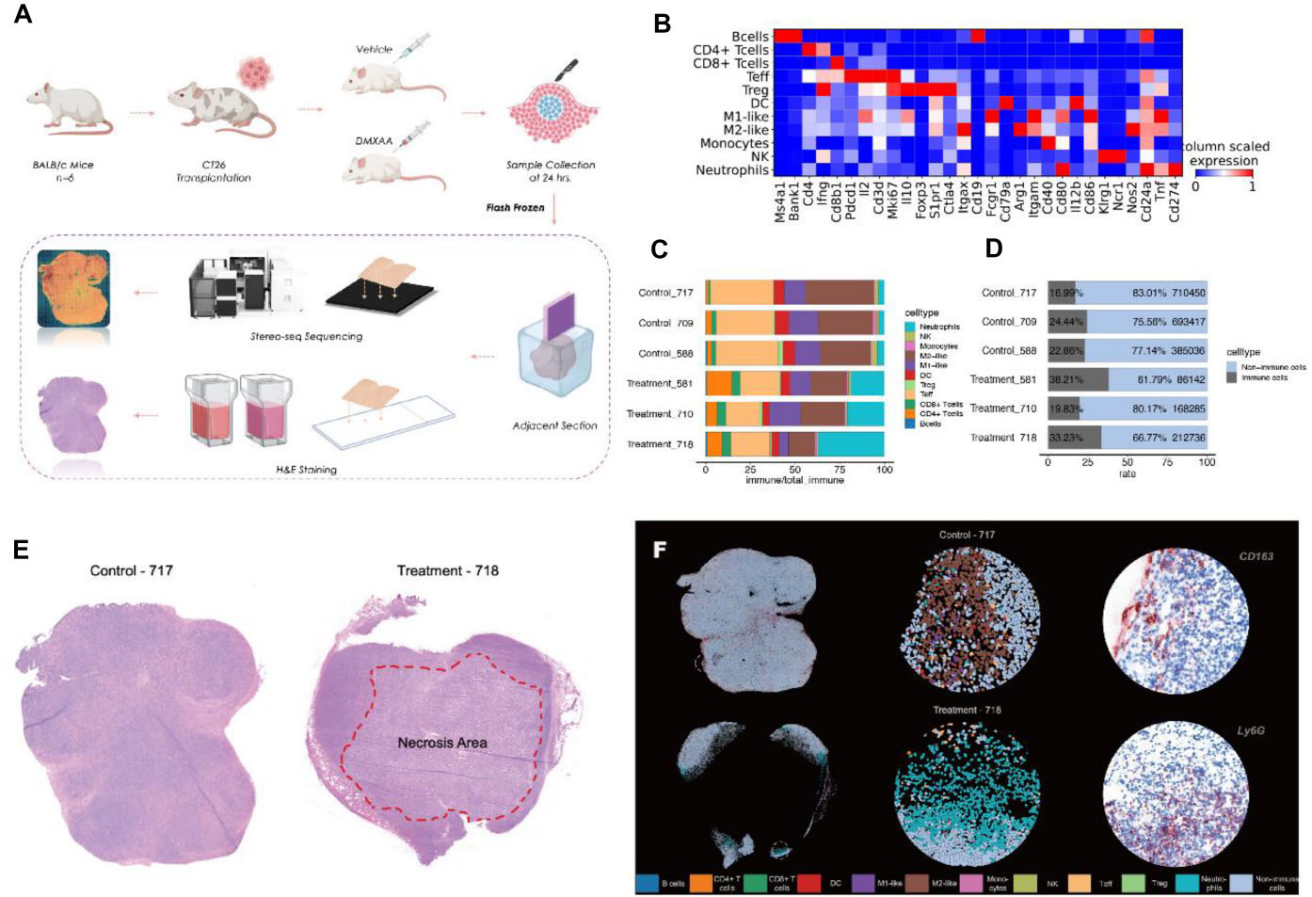
Spatial transcriptomic mapping of xenograft model *in situ*. (A) Flowchart illustrating the construction of the xenograft model is presented. BALB/c mice were subcutaneously injected with CT26 cells derived from colon cancer. Subsequently, the mice were either treated with a vehicle or DMXAA for a period of 14 days posttumor transplantation. Tissue samples were collected 24 hours after the treatment. (B) Heatmap depicting the expression of transcriptional markers of 11 annotated immune cell types at a single-cell resolution is shown. (C) An analysis of the proportion of immune cells at a single-cell resolution in each sample is provided. (D) Histogram comparing nonimmune cells to immune cells is displayed, where the blue bar represents the proportion of nonimmune cells, and the gray bar represents the integrated proportion of immune cells. The numbers associated with each bar indicate the precise cell counts for each sample. (E) Representative hematoxylin and eosin staining images of sample 717 from the control group (left) and sample 718 from the treatment group (right) are presented. The red-thread restricted region highlights the necrotic site. (F) *In situ* visualization of annotated cell types using stereo-seq data is shown on the left, an enlarged image of the marked-circle-site displaying cellular compositions is in the middle, and representative immunohistochemistry staining (CD163 indicating macrophages in sample 717 and Ly6G indicating neutrophils in sample 718) of the same marked-circle-site is presented on the right.

To address iTME-associated research questions, we applied the designed framework on ST datasets from xenograft models (Fig. 4A) with immune agonist (STING agonist) treatment [30]. Spatially resolved transcriptomic data from xenograft tumor tissues were collected using stereo-seq, a spatial sequencing technology with a subcellular resolution of 500 nm [17]. A data matrix at the resolution of single cells was obtained after cell segmentation processing based on nuclear staining [22]. By employing cell2location-induced deconvolution of the spatial transcriptomic matrix with a reference previously reported [16], we identified and validated 12 distinct cell types (Fig. 4B), including 6 of lymphoid lineage, 5 of myeloid lineage, and 1 of a nonimmune cluster. The proportion of cell types across samples (Fig. 4C) revealed varying compositions of immune cells. Further quantitative analysis showed fewer cell numbers in the treatment group compared to the control (Fig. 4D). It was suggested that necrosis caused by the treatment might contribute to the reduction in cell numbers in the treatment group (Fig. 4E). Notably, control groups had a higher frequency of M2-like macrophages, while treatment groups had higher frequencies of neutrophils (Fig. 4C). Interestingly, we also observed a location preference (Fig. 4F) of neutrophils in the treatment group, where they tended to cluster around necrotic niches, whereas other cells like M2-like macrophages in the control group were randomly distributed. However, methods exploring the correlation between specific bioactivities and their spatial preferences were rarely utilized. To validate our hypothesis of spatial preference among different cell types, we conducted an analysis of CN in the following section.

### Decoding TME-associated cellular neighborhood using StereoSiTE

**Figure 5:**
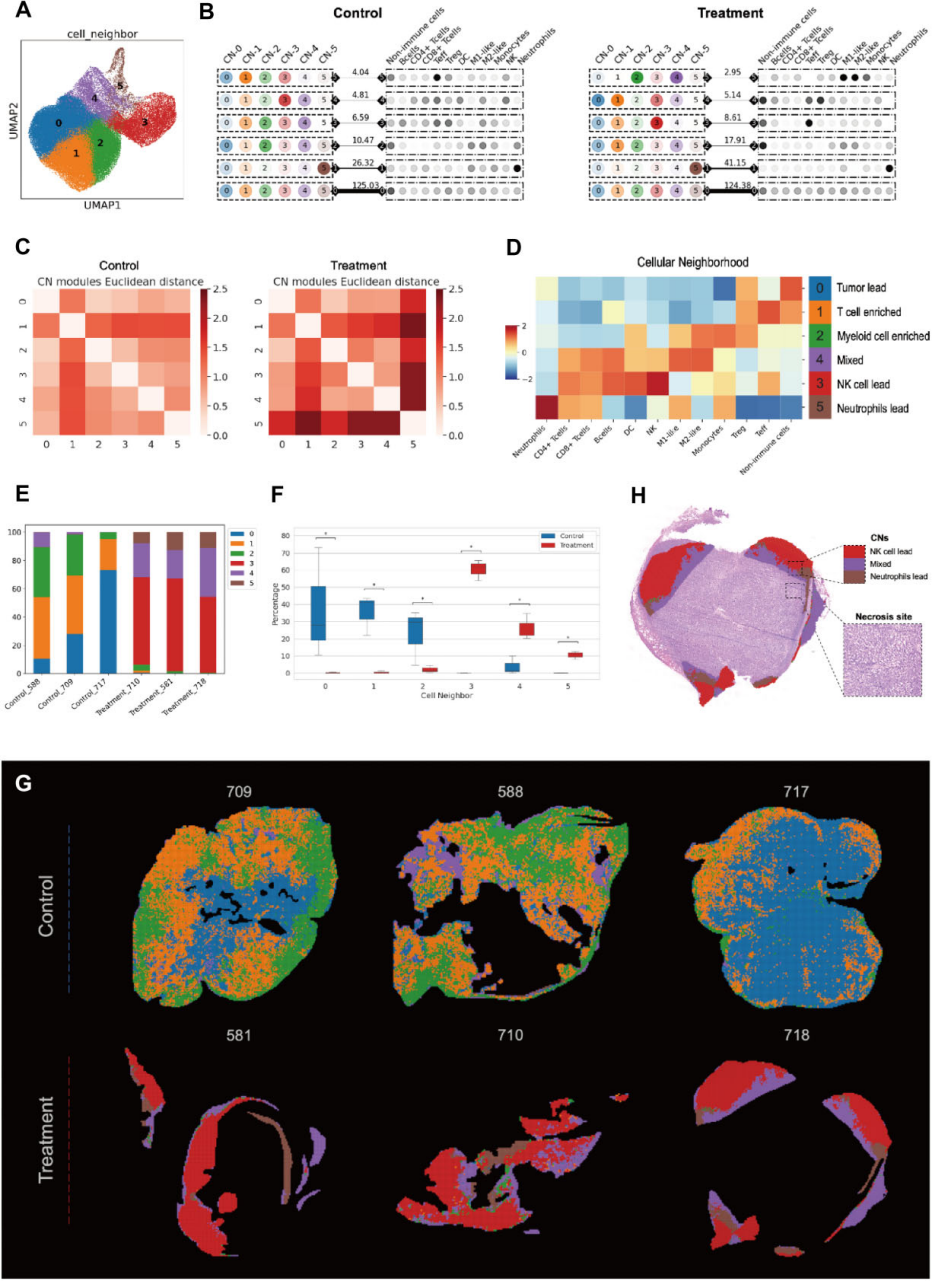
Construction of a cellular neighborhood. (A) UMAP exhibiting the deconvolution of the identified CN clusters at a bin size of 100. (B) Tucker tensor decomposition was applied to control (left) and treatment (right) samples to stratify CN modules and CT modules. The crosstalk extent of associated CN and CT was depicted by the weight of the line with indicated numbers. (C) Heatmap showing the Euclidean distance between CN modules constructed in the control (left) and treatment (right) groups, respectively. (D) Heatmap indicating varied cell composition in different CNs (left) and confetti labeling the corresponding title for each CN (right). (E) Distribution of CN frequencies in different samples from the control and treatment groups. (F) Boxplot illustrating the statistical variation of CN frequency across groups. (G) Spatial distribution of CNs in different groups. (H) Projection of CN5 onto adjacent H&E staining of sample 718, with an enlarged image of the marked-circle-site displaying a highly resolved H&E staining of the necrotic area.

The heterogeneity of the iTME is prevalent both intra- and intertumor, primarily due to diverse cell organizations within each spatially compartmentalized unit. To better understand and elucidate the iTME of xenografts, particularly in the absence of distinct histological characteristics, visualization of tissues with CNs is crucial. In order to identify iTME units that are consistently preserved across samples, we integrated a matrix that simultaneously encompasses CNs and CTs and introduced Tensor to decompose the module matrix. Initially, we clustered windows of varying sizes, labeling all samples and identified unique and exclusive CNs under different benchmarks (Fig. 5A and Supplementary Fig. S2A, B). Through tensor decomposition in different groups (Fig. 5B and Supplementary Fig. S2C), we observed specific CNs correlating with particular CTs in each individual module (Fig. 5B) and distinct Euclidean distances reflecting intermodule heterogeneity (Fig. 5C and Supplementary Fig. S2D) within the context of a bin size of 100 microns, prompting us to further investigate this index. As anticipated, the composition of CTs within each CN significantly varied across the cohort, indicating that different immune cells tend to colocalize and interact with specific cell types in compartmentalized iTME units. Consequently, we categorized each CN based on their predominant cell proportions (Fig. 5D). Subsequently, we calculated the frequencies of CNs in different groups (Fig. 5E) and noted a distinct correspondence of CN3 (NK cells lead), CN4 (mixed), and CN5 (neutrophils lead) in the treatment group (Fig. 5F), which aligned with the treatment context and the tensor indication. To spatially visualize and evaluate CNs (Fig. 5G), we specifically focused on assessing CN5 *in situ*, as neutrophils were notably recruited by chemokine stimulation but not consistently present in targeted tissues [31–33]. We projected CNs orthotopically onto adjacent hematoxylin and eosin (H&E) staining to examine the potential distribution pattern of this neutrophil-dominant iTME unit (Fig. 5H and Supplementary Fig. S2E). An evident trend of CN5 colocalizing around necrotic edges compared to other CNs was observed, potentially highlighting regional bioactivities exerted by tumor cells following immunoagonist treatment and indicating alignment between tensor-indicated transcriptomic traits and histological features. Consequently, we decided to further investigate the burst of bioactivities in CN5 in subsequent studies.

### Deciphering the molecular mechanisms within specific CN regions associated with treatment response

**Figure 6:**
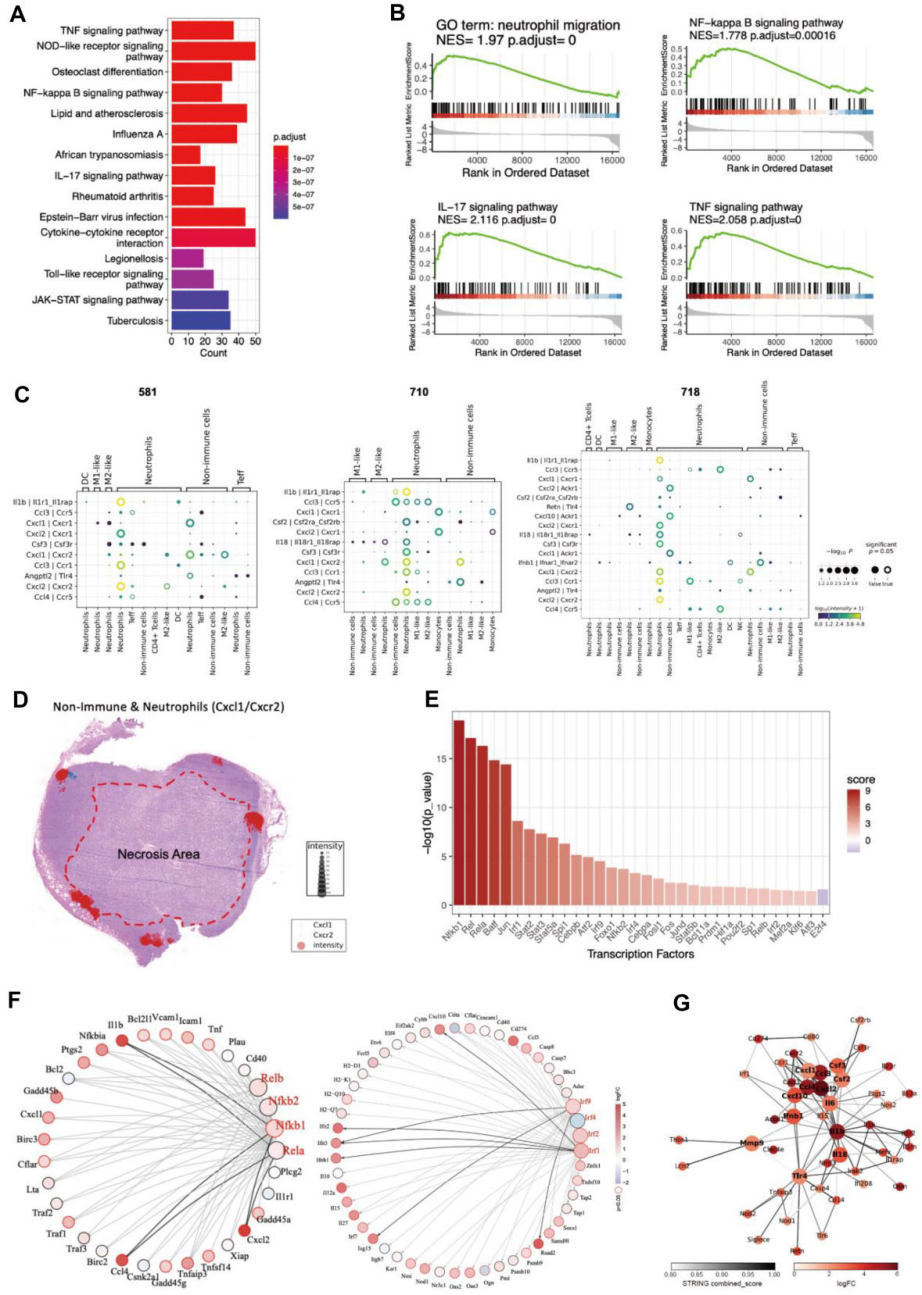
Molecular mechanism within the CN regions of interest. (A) KEGG analysis of upregulated signaling pathways in CN5 compared to those in counterpart CNs. (B) GSEA analysis was performed to assess the indicated signaling pathways in CN5 compared to those in counterpart CNs. (C) Representative LR interactions in CN5 of each sample from the treatment group inferred by SCII. (D) *In situ* visualization of the communication between nonimmune cells and neutrophils mediated by Cxcl1-Cxcr2 was carried out, with a projection to an adjacent H&E image to exhibit the crosstalk coordinates. (E) TF regulon analysis of CN5; score represents the potency of transcriptional factor activity. (F) Graph indicating predicted interactions between genes that were differentially expressed in CN5 (red, transcription factors; black, other genes); the left exhibits the Nfkb1-centric network and the right exhibits the Irf-centric network. (G) PPI network analysis in CN5 of the treatment group. The color intensity of nodes represents the logFC of the indicated gene expression, while the edges represent the STRING database score (confidence level). Hub genes are highlighted in bold font and larger size.

To comprehensively illustrate the unique landscape induced by the STING agonist, an analysis of DEGs was initially conducted. Each CN was compared to the other counterparts in the treatment group, revealing a significant recruitment of neutrophils and activation of STING signaling specifically in CN5 [34, 35] (Fig. 6A, B and Supplementary Fig. S3A). Furthermore, other CNs in the treatment group exhibited distinct phenotypes (Supplementary Fig. S3B), underscoring the drug response characteristics embedded in CN5. Given the predominant presence of neutrophils in this context, the next step was to elucidate the cellular-level signature activities of CN5 using SCII to analyze cell–cell communications at a single-cell resolution. The analysis of CN5 revealed robust interactions between nonimmune cells and neutrophils, with notable pairs such as Cxcl1-Cxcr2, and also highlighted significant neutrophil–neutrophil communication with elevated expression of LR pairs like Il1b-Il1r1 and Cxcl2-Cxcr2 (Fig. 6C and Supplementary Table S2). Additionally, the spatial distribution of Cxcl1-Cxcr2 projected on adjacent H&E images demonstrated frequent crosstalk between nonimmune cells and neutrophils around necrotic areas (Fig. 6D and Supplementary Fig. S3B). To further explore the LR pairs associated with treatment response, functional signaling pathways in CN5 were investigated. Through *in silico* identification of active transcription factors and potential interactions (Fig. 6E), an Nf-kappa b-centric network and an Irf1-centric network were identified, coordinating the upregulation of signature downstream targets such as Il1b and Ifnβ, respectively (Fig. 6F). Notably, Irf1 was found to regulate Ifnβ expression, while Irf3 showed minimal activation at this treatment stage (Supplementary Fig. S3D). Subsequently, protein–protein interaction (PPI) analysis was conducted to construct the signaling network and identify hub genes responsible for the signature activities in CN5. Remarkably, Il1b emerged as the top-scoring gene frequently interacting with other proteins, including Ccl4, Cxcl2, Cxcl1, and Il6 (Fig. 6G), serving as a downstream target of NF-kappa B in the context of STING signaling activation [34]. The analysis of transcription factor (TF) regulon and the PPI network collectively suggested that neutrophils may play pivotal roles in regulating immune activities in response to STING agonist through signature signaling pathways such as Nf-kappa-b and Irf1. In summary, we have presented an integrated analytical approach to delineate functional iTME at both molecular and cellular levels.

## Discussion

The emergence of spatial transcriptomics data at the single-cell resolution has brought a significant transformation in the field [17]. This advancement enables researchers to depict the authentic landscape of TME without losing spatial information due to dissociation processes. The cutting-edge sequencing technique, characterized by its multidimensional nature and high-throughput gene expression profiling, requires a robust algorithmic framework to effectively associate specific cell populations with their functional roles, biological functions, and clinical implications in order to address fundamental scientific inquiries [35]. In this study, we introduce a novel analytical framework named StereoSiTE, which combines publicly available algorithms with in-house developed algorithms to unveil the underlying biological processes by identifying spatially organized CNs and inferring the spatial cell–cell communications within these identified CN regions.

The application of CN analysis in StereoSiTE facilitated the identification of crucial regions within the iTME, indicating neighboring cellular compositions that are more likely to engage in active interactions due to their proximity. By dividing the tissue domain, CN-based analysis accurately pinpointed cell types with spatial signatures that might have been overlooked due to their low abundance and transcriptomic activities across the entire slide. Following the targeting of iTME regions of interest using CN analysis, the proprietary algorithm SCII in StereoSiTE was employed to unravel the spatial intercellular communications within these functional units. SCII constructs a cell graph by linking cell pairs within a specified radius threshold, which can be adjusted based on the type of communication. For secreted signaling, a radius threshold of 100 to 200 µm is recommended, while for cell–cell contact involving LR pairs, the cell size should be taken into consideration. SCII integrates the LR database from CellChatDB, which offers a catalog linked to communicative distances. However, SCII is compatible with any other LR database. A comparative analysis was conducted among CellPhoneDB, CellChat, Giotto, SpaTalk, and SCII using the same stereo-seq data matrix. Figure 3 illustrates that an analysis without a distance threshold could yield false-positive results, with SCII demonstrating superior performance compared to other methods. Furthermore, LR pairs enriched in the CN of interest within the distance threshold exhibit a high potential for functional associations.

To demonstrate the overwhelming performance of StereoSiTE, we applied the framework in a research scenario to investigate the functional CN regions and the cellular and molecular mechanisms in response to immunoagonist treatment within them. We identified CN5, which was led by neutrophils situated around the necrotic regions, indicating the recruitment of neutrophils. Pathway analysis revealed that the STING signaling pathway was distinctly activated in this CN region, while SCII analysis showed frequent communication between neutrophils and neutrophils/nonimmune cells mediated by Cxcl1-Cxcr2. Further analysis of TF and PPI networks indicated that the most influential LR pair was the downstream targets of the STING-activated signaling—NF-kappa B [36]. Altogether, StereoSiTE depicted a neutrophil-led iTME unit by characterizing the regional phenotype from both molecular and cellular perspectives, offering insights into the mechanisms exerted by neutrophils in iTME.

StereoSiTE has effectively elucidated the molecular and cellular mechanisms underlying specific iTME regions by integrating gene expression data with spatial information. The tool’s modular design enables users to easily interchange or combine analysis modules and the LR database to meet their specific needs. However, the construction of the spatial cell graph required by the SCII module currently limits its ability to analyze spatial omics data at a single-cell resolution. With the continuous advancement of spatial omics technology leading to higher resolutions, it is anticipated that StereoSiTE will evolve as a robust and adaptable analytical framework for unraveling the complexities of the iTME. This is anticipated to offer novel insights in cancer research and enhance drug discovery endeavors. Furthermore, considering the current limitations of the functional modules in StereoSiTE, there are plans to expand them to enable the tool to interpret spatial transcriptomics factor activities and spatial pathway enrichment in spatial transcriptomics datasets from various perspectives.

## Methods and Materials

### Mice and cell lines

Female BALB/c mice aged 6 weeks were procured from GemPharmatech Co., Ltd. The mice were accommodated in a specific pathogen–free animal facility at GemPharmatech Co., Ltd. CT26 colon cancer cells were purchased from ATCC. Cells were cultured at 37°C in a 5% CO_2_ environment in Dulbecco’s modified Eagle medium supplemented with 10% fetal bovine serum and 1% penicillin/streptomycin.

### Xenograft tumor models and treatment

We implanted 5 × 10^5^ cells/100 μL of CT26 cells into the right flanks of BALB/c mice. Upon reaching a tumor volume of 250–300 mm^3^, intratumoral injections of the STING agonist (0.5 mg/50 μL/mouse, DMXAA; Vadimezan) were administered (43). Mice in the control group received intratumoral injections of an equivalent volume of phosphate-buffered saline. Xenograft tumor samples were harvested 24 hours posttreatment and embedded in OCT on dry ice.

### Stereo-seq library preparation and sequencing

#### Tissue processing

Two consecutive cryo sections of 10 μm thickness were prepared. One section was mounted on a glass slide and subjected to H&E staining using a previously established protocol. The second section was adhered to the surface of the stereo-seq chip and incubated at 37°C for 3–5 minutes. Subsequently, the sections were fixed in methanol and incubated at −20°C for 40 minutes. The preparation of the stereo-seq library and the sequencing process adhered to a previously published protocol [17].

#### In situ reverse transcription

The prepared section underwent processing in accordance with the Stereo-seq Transcriptomics Set User Manual (STOmics), utilizing reagents from the Stereo-seq Transcriptomics T kit and Stereo-seq Library Preparation kit (STOmics). Initially, tissue sections on the chip were washed with PR rinse buffer and subsequently permeabilized at 37°C for 10 minutes. The RNA released from the permeabilized tissue was captured by the probe and subjected to overnight reverse transcription at 42°C. Following reverse transcription, the tissue sections were treated with tissue removal buffer at 55°C for 30 minutes to digest them. Subsequently, the resulting cDNA was amplified.

#### Amplification

The cDNAs that were collected underwent amplification using KAPA HiFi Hotstart Ready Mix (Roche, KK2602) with 0.8 μM cDNA-PCR primer. The PCR reactions were carried out in a series of steps, starting with an initial incubation at 95°C for 5 minutes, followed by 15 cycles at 98°C for 20 seconds, 58°C for 20 seconds, and 72°C for 3 minutes, and concluded with a final incubation at 72°C for 5 minutes.

#### Library construction and sequencing

The concentrations of the PCR products were quantified using the Qubit dsDNA Assay Kit (Thermo, Q32854). A total of 20 ng DNA was then fragmented with in-house Tn5 transposase at 55°C for 10 minutes. The reactions were stopped by adding 0.02% SDS and gently mixing at 37°C for 5 minutes. Fragmented products were amplified as follows: 25 μL of fragmentation product, 1 × KAPA HiFi Hotstart Ready Mix, 0.3 μM Stereo-seq-Library-F primer, and 0.3 μM Stereo-seq-Library-R primer in a total volume of 100 μL with the addition of nuclease-free H_2_O. The reaction was then run as 1 cycle at 95°C for 5 minutes; 13 cycles at 98°C for 20 seconds, 58°C for 20 seconds, and 72°C for 30 seconds; and 1 cycle at 72°C for 5 minutes. PCR products were purified using the AMPure XP Beads (0.6× and 0.15×) for DNA nanoball (DNB) generation and finally sequenced on an MGI SEQ-2000 sequencer.

### Data analysis

#### Raw sequencing data analysis

Fastq files were generated using the MGI SEQ-2000 sequencer. The process involved cell nuclei staining image stitching, tissue segmentation, gene expression registration, and genome mapping, followed by gene count analysis. These procedures were conducted utilizing the Stereo-seq Analysis Workflow (SAW) available at https://github.com/STOmics/SAW. The stitched cell nuclei staining images were utilized to create single-cell nuclei masks through the application of a cell segmentation script obtained from the StereoCell tool [22] (accessible at https://github.com/STOmics/StereoCell/tree/dev/cellbin/cell_segmentation/segment.py). The script is based on the psaUnet architecture, which integrates Deep Residual Net, U-Net, and EPSANet. Subsequently, the gene expression matrix for each cell was generated by aligning the spatial expression profile matrix with its corresponding single-cell nuclei mask based on spatial coordinates. The expression profile matrix was divided into nonoverlapping bins covering a 100 × 100 DNB area (bin100) for subsequent cellular neighborhood establishment and functional enrichment analysis. The data structure was then established using Scanpy in Python 3.9 for further analytical processes.

### Cell-type annotation

We utilized a single-cell transcriptomics dataset of the mouse colon cancer cell line CT26 [16] as a reference to deconvolute a mixture of 11 immune cell types and nonimmune cells in our stereo-seq data using cell2location with hyperparameters N_cells_per_location = 1 and detection_alpha = 20. The predominant cell type was assigned to each cell, followed by the calculation and visualization of cell-type frequencies using the R package ggplot2.

### Cellular neighborhood construction

The tissue samples were binned into adjacent windows, each measuring 100 × 100 DNBs (bin100), forming squares with a side length of 100 DNB (the unit representing a capturing site). Given that the distance between neighboring sites was 500 nm, bin100 represented a square with a side length of 50 µm. Subsequently, the cellular composition of each bin100 was deconvoluted by aligning the gene expression profiles of different cell types from a single-cell transcriptomic dataset [16] to the spatial data using cell2location. Based on the deconvoluted cell composition matrix, all sample windows were then grouped into 7 CNs through the application of the KNN graph and Leiden clustering with parameters n_neighbors = 19 and resolution = 0.32. Consequently, windows with similar cell compositions were aggregated to create distinct microenvironments. For each CN, the abundance of cell types across all windows within the CN region was aggregated to calculate percentages, which were then visualized using the Python module Seaborn.

### Benchmarking analysis for deconvolution methods

The study conducted a comparative analysis of the cell-type deconvolution capabilities of cell2location and several recently developed deconvolution software tools, namely Celloscope [12], POLARIS [14], and GraphST [13]. The evaluation was based on the benchmark pipeline provided by Kun Qu’s laboratory, accessible at https://github.com/QuKunLab/SpatialBenchmarking. The assessment utilized the mouse visual cortex STARmap dataset (“20180505_BY3_1kgenes”) at a single-cell resolution spatial transcriptome, in conjunction with the corresponding smart-seq data available at https://portal.brain-map.org/atlases-and-data/rnaseq/mouse-v1-and-alm-smart-seq, and simulated the spot-level spatial transcriptome for benchmarking purposes. Performance evaluation of the 4 methods in predicting the cell-type composition of spots or the distribution of cell-type clusters was carried out using metrics such as the Pearson correlation coefficient (PCC), structural similarity index (SSIM), root mean square error (RMSE), Jensen–Shannon divergence (JSD) score, and a combined metric known as the Accuracy Score (AS), which aggregates the aforementioned 4 metrics. The predicted results of STARmap by cell2location from Kun Qu’s group were directly compared in the analysis.

A public stereo-seq dataset of liver cancer was employed for benchmarking analysis [37]. The gene expression matrix was binned into 25 × 25 µm pseudo-spots (approximately 1 cell) following established protocols. To optimize computational efficiency, only the lower right quadrant of the expression matrix was utilized for testing purposes. The kappa score was employed to assess the concordance between the software-predicted outcomes and the documented results, given the absence of a definitive ground truth for the stereo-seq liver dataset.

The cell-type deconvolution process followed the tutorials of each software utilized. For cell2location (https://github.com/BayraktarLab/cell2location) with stereo-seq liver data, the regression model of single-cell reference data was trained with parameters max_epochs = 1,500. The cell2location model was trained with parameters max_epochs = 5,000 and N_cells_per_location = 3. When using Celloscope (https://github.com/szczurek-lab/Celloscope) with STARmap data, the number of cells in each spot was determined based on ground truth. In the case of stereo-seq liver data, the number of cells in each spot was set to 1, and number_of_chains was set to 10, while other parameters were maintained at default values. For POLARIS (https://github.com/JiawenChenn/POLARIS) with STARmap data, the layer label was assigned following Kun Qu’s benchmark research. In the context of stereo-seq liver data, all spots’ layer labels were uniformly set to 1. Lastly, for GraphST (https://github.com/JinmiaoChenLab/GraphST), all genes were designated as high variable genes for STARmap data, whereas stereo-seq liver data was configured with 5,000 high variable genes.

### Benchmarking analysis for tissue region division methods

We utilized the STARmap dataset to conduct a comparative analysis of the accuracy of spatial domain detection between CN and other local niches software (BANKSY [24], Giotto [6]) by evaluating the adjusted Rand index (ARI). The STARmap dataset was simulated with the 835 × 835-pixel spot-level spatial transcriptome following Kun Qu’s benchmark pipeline. In the stereo-seq dataset, the default binsize of pseudo-spots for CN analysis was set to 100, equivalent to 50 × 50 µm. The total dimensions of the STARmap sample were 1,400 × 300 µm. Each square spot in the STARmap dataset, based on spatial coordinates, approximately corresponded to 835 × 835 pixels, equivalent to bin100 of stereo-seq data. The cell-type percentage for each spot was computed for CN analysis. By setting parameters n_neighbors = 15 and resolution = 1.1, we identified 7 clusters (representing the number of spatial domains in the dataset) for ARI calculation. Given that Shyam Prabhakar’s group had previously determined the ARI of BANKSY and other software in the STARmap dataset [15], we subsequently calculated the ARI of the CN results and directly compared them with their findings.

### Tensor decomposition

For each group, a tensor with dimensions of 3 × 7 × 12 (representing 3 samples, 7 CNs, and 12 cell types) was constructed. Nonnegative Tucker decomposition was conducted using the Python package Tensorly [38]. The suitable rank for nonnegative tensor decomposition was determined by calculating the decomposition losses for various combinations of the number of CN modules and CT modules, selecting the rank at the elbow point (Supplementary Fig. S1B). The visualization of the decomposition results is based on Schürch et al. [34].

### Functional enrichment analysis and transcription factor activity inference

Differential expression analysis was conducted on different CNs using the edgeR package [39] in a pseudobulk manner [40]. Genes were considered differentially expressed when the absolute value of log fold changes > 1 and *P* < 0.05. To elucidate the biological function of CN5, KEGG enrichment analysis, gene ontology enrichment analysis, and gene set enrichment analysis (GSEA) were performed using the functions of the R package ClusterProfiler [41]. The top 15 significantly enriched pathways from the KEGG enrichment analysis and gene ontology enrichment analysis were visualized in bar plots, respectively. The noteworthy pathways identified through GSEA were presented using the gseaplot2 function. TF activity inference was carried out using the R packages decoupleR [42] and DoRothEA [43] with the univariate linear model. The regulatory network of the most significantly associated TFs was visualized using the igraph package.

### Spatial cell interaction intensity

Initially, the spatial nearest neighbor graph was created using the spatial coordinates of all cells. Cell pairs within a distance less than the specified radius threshold were linked by edges. Subsequently, the edges were weighted by aggregating the expression levels of ligand and receptor genes from the sender and receiver cells (nodes at the ends of the edge). Edges with a weight of 0 (indicating no coexpression) were eliminated, while edges with a weight greater than 0 (indicating coexpression) were retained. Lastly, the local spatial cell interaction intensity of each sender cell with its connected receiver cells was calculated by summing the edge weights between them. This interaction intensity is defined as:


(1) \begin{eqnarray*}
\textit{intensity} &=& \sum\nolimits_{(i = 0)}^n {\textit{edgeWeight}_i(\textit{receiver}_i)} \\
&&\qquad\left( {{\mathrm{n}} = {\mathrm{number\,\,of\,\,surrounding\,\,receiver\,\,cells}}} \right)
\end{eqnarray*}

Furthermore, the interaction intensities from sender cells to receiver cells mediated by specific LR pairs across the entire slide were equivalent to the total weight of all edges. The following formula delineates the calculation procedure:


(2) \begin{eqnarray*}
\textit{intensities} = \sum\nolimits_{(i = 0)}^N {\textit{edgeWeight}_i} \,\,(N = \textit{total}\,\,\textit{edges}\,\,of\,\,\textit{whole}\,\,\textit{slide})
\end{eqnarray*}

In the case of a complex ligand or receptor consisting of multiple subunits, we opted for the minimal expression or calculated the average expression of all subunits to compute the SCII.

In our research, we utilized the LR database from CellChatDB due to its precise categorization of communication types linked to various active distances. Moreover, the database selection process is adaptable and can be customized by the user. This feature enables users to incorporate LR databases that are of particular interest to them. The database file must adhere to CSV format standards and include columns “source” and “target,” which indicate the ligand and receptor. Additionally, if users intend to analyze different LR types using distinct strategies, the inclusion of a column “annotation” indicating LR types is required.

To investigate the superiority of the SCII method in comparison to the cell–cell interaction (CCI) method, disregarding cell spatial distribution, we assessed the distance of inferred interactions by counting the median distance within the cell graph. This cell graph was established by utilizing the KNN algorithm to link each cell with its closest K neighboring cells.

### Comparison between SCII with other CCI methods

Most of the other methods for inferring cell–cell interactions (such as CellPhoneDB, CellChat, Giotto, Spatalk) are implemented in R, which limits their computational performance. This limitation hinders the analysis of entire stereo-seq datasets from a single sample containing 403,516 cells. To address this issue, we extracted a smaller tissue region from the original dataset, comprising 11,214 cells. Due to variations in the LR pairs database among different methods and the inability of some methods to handle protein complexes, we identified the common LR pairs between CellPhoneDB and CellChatDB. LR pairs associated with complexes were then filtered out, leaving behind LR pairs that were utilized for cell–cell interaction inference by each method. While adjusting certain cell and gene filter parameters due to the constrained gene capture of spatial transcription technology at a single-cell resolution, all methods were benchmarked using default settings. The overlap of inferred interactions from different methods was calculated using set operations and the Jaccard index. Additionally, the coexpression percentage of the cell–cell interactions was determined by quantifying the number of spatially proximal sender and receiver cell pairs expressing corresponding ligand and receptor genes from the cell nearest neighbor graph.

### Protein–protein interaction analysis

We queried 628 significantly upregulated genes (log fold change > 2 and false discovery rate (FDR) < 0.05) in the CN5 area of treatment samples using the STRING v11.5 database [44] (score cutoff = 0.4). Among these, 505 proteins were found. After filtering out 95 nodes with a degree of 0, a PPI network consisting of 402 proteins and 2,042 edges was constructed. Subsequently, we applied the Markov clustering (MCL) algorithm [45] with a parameter (I = 3.0) to generate a functional PPI network. The largest cluster, comprising 82 proteins and 821 edges, was further analyzed to identify hub genes by ranking their degree and maximal clique centrality (MCC) score using the Python module NetworkX 3.1 [46]. The hub genes were determined based on the highest MCC scores within the top 10 degrees, and their interactions with a STRING confidence score above 0.8 were considered key PPI networks.

### STAR

**Table:** 

Stereo-seq Analysis Workflow (SAW)	https://github.com/STOmics/SAW
Python 3.9	https://www.python.org/
Numpy 1.22.4	https://numpy.org/
Pandas 1.5.1	https://pandas.pydata.org/
Sklearn 1.0.1	https://scikit-learn.org/
Cell2location 0.1	https://cell2location.readthedocs.io/en/latest/
Scanpy 1.9.1	https://scanpy.readthedocs.io/en/stable/index.html
Tensorly 0.7.0	http://tensorly.org/stable/index.html
Seaborn 0.11.2	https://seaborn.pydata.org/index.html
Squidpy 1.1.2	https://squidpy.readthedocs.io/en/stable/index.html
NetworkX 3.1	https://networkx.org/
STRING v11.5	https://string-db.org/
mcl 22–282	https://github.com/micans/mcl
R 4.2.1	https://www.r-project.org/
ggplot2 3.4.0	https://ggplot2.tidyverse.org/
ClusterProfiler 4.6.0	https://bioconductor.org/packages/release/bioc/html/clusterProfiler.html
edgeR 3.40.2	https://bioconductor.org/packages/release/bioc/html/edgeR.html
Dorothea 1.10.0	https://bioconductor.org/packages/release/data/experiment/html/dorothea.html
decoupleR 2.4.0	https://www.bioconductor.org/packages/release/bioc/html/decoupleR.html
igraph 1.4.3	https://r.igraph.org/
CellPhoneDB v4	https://cellphonedb.readthedocs.io/en/latest/index.html
CellChat v2	https://github.com/jinworks/CellChat
Giotto	https://drieslab.github.io/Giotto_website/
SpaTalk	https://github.com/ZJUFanLab/SpaTalk
Celloscope	https://github.com/szczurek-lab/Celloscope
POLARIS	https://github.com/JiawenChenn/POLARIS
GraphST	https://deepst-tutorials.readthedocs.io/en/latest/
BANKSY	https://github.com/prabhakarlab/Banksy

## Availability of Source Code and Requirements

Project name: StereoSiTE.

Project homepage: https://github.com/STOmics/StereoSiTE.

Operating system: Linux.

Programming language: Python.

Other requirements: Python3.9 or higher.

License: GPL-3 license.

BiotoolsID: stereosite.

RRID: SCR_025,236.

## Supplementary Material

giae078_GIGA-D-23-00276_Original_Submission

giae078_GIGA-D-23-00276_Revision_1

giae078_GIGA-D-23-00276_Revision_2

giae078_Response_to_Reviewer_Comments_Original_Submission

giae078_Response_to_Reviewer_Comments_Revision_1

giae078_Reviewer_1_Report_Original_Submission Lihong Peng -- 12/23/2023 Reviewed

giae078_Reviewer_1_Report_Revision_1 Lihong Peng -- 12/23/2023 Reviewed

giae078_Reviewer_1_Report_Revision_2 Lihong Peng -- 8/23/2024 Reviewed

giae078_Reviewer_2_Report_Original_Submission Chenfei Wang -- 12/25/2023 Reviewed

giae078_Reviewer_2_Report_Revision_1 Chenfei Wang -- 5/4/2024 Reviewed

giae078_Reviewer_3_Report_Original_Submission Rosalba Giugno -- 1/12/2024 Reviewed

giae078_Reviewer_3_Report_Revision_1 Rosalba Giugno -- 5/17/2024 Reviewed

giae078_Supplemental_Figures_and_Tables

## Data Availability

The data that support the findings of this study have been deposited into CNGB Sequence Archive (CNSA) of the China National GeneBank DataBase (CNGBdb) with accession number CNP0004910 and National Center for Biotechnology Information with accession number PRJNA1087118. Snapshots of our code and other data further supporting this work are openly available in the *GigaScience* repository, GigaDB [47].

## Additional Files

**Supplementary Fig. S1**. Performance comparison among different cell-type deconvolution methods, tissue domain division methods, and intercellular communication inference methods. (A) The performance correlation coefficient (PCC), structural similarity index (SSIM), root mean square error (RMSE), and Jensen–Shannon divergence (JSD) values were calculated to assess the accuracy of cell-type composition in the STARmap dataset. These metrics were evaluated for both individual spots (top) and clusters (bottom) using 4 deconvolution methods. The aggregated accuracy scores for spots or clusters were derived from the PCC, SSIM, RMSE, and JSD values. (B) The spatial distribution of cell types predicted by each deconvolution method was examined in the stereo-seq dataset of liver cancer. A comparison was made between the results and the original cell-type annotations from the primary literature. The agreement between the different annotations was quantified using the kappa statistic (bottom left). (C) The spatial distribution of tissue domains segmented by cellular neighborhood (CN) was analyzed in the STARmap dataset. (D) Bar plot displaying the adjusted Rand index (ARI) for the domain division results obtained from BANSKY, CN, and Giotto HMRF in the STARmap dataset. (E) Runtimes of CN and BANSKY for increasing cell numbers, up to 2 million cells. (F, G) Null distributions were generated through permutation tests for each interaction (top) produced by both CellPhoneDB and SCII, as well as by SCII alone. The red arrow indicates the actual intensity measured by SCII. The spatial distribution of connections between sender cells and receiver cells is shown at the bottom, with a zoomed-in region displayed for clearer observation. (H) A subset of the stereo-seq demo data was extracted to compare the SCII method with other cell–cell interaction (CCI) inference techniques. The selected region is highlighted within a rectangular box. (I) Venn diagram was used to visualize the overlap and distinctions in the inferred interactions from various CCI inference methods, including CellphoneDB v4, CellChat v2, Spatalk, and Giotto.

**Supplementary Fig. S2**. Construction of cellular neighborhood. (A) UMAP exhibiting the deconvolution of the identified CN clusters at a bin size of 150 (left) and 200 (right). (B) Rank selection of Tucker tensor decomposition at different bin sizes to stratify CN modules and CT modules. The tensor decomposition loss is shown in different CN modules (in different colors) or CT modules numbers (x-axis). (C) Decomposition results for both groups at bin sizes of 150 and 200. The crosstalk extent of associated CN and CT is represented by the weight of the line with indicated numbers. (D) Heatmap showing the Euclidean distance between CN modules constructed in the control (left) and treatment (right) groups at the indicated bin sizes, respectively. (E) Projection of CN5 on adjacent H&E staining of samples 518 (left) and 710 (right).

**Supplementary Fig. S3**. Deconvolution of CN of interest. (A) GSEA analysis of indicated signaling pathways in CN5 compared to those in counterpart CNs. (B) KEGG analysis of upregulated signaling pathways respectively in CN4 (left) and CN3 (right) compared to that in counterpart CNs. (C) *In situ* visualization of Cxcl1-Cxcr2 between nonimmune cells and neutrophils in 518 (left) and 710 (right) with adjacent H&E image displayed to exhibit the crosstalk coordinates. (D) Boxplot of Irf3 expression in control groups and treatment groups (*P* = 0.4).

**Supplementary Table S1**. False-positive interactions inferred by CellPhoneDB.

**Supplementary Table S2**. Interactions within the CN5 regions inferred by SCII.

## Abbreviations

ARI: adjusted Rand index; AS: Accuracy Score; CCI: cell–cell interaction; CN: cellular neighborhood; CT: cell type; DC: dendritic cell; DEG: differentially expressed gene; GSEA: gene set enrichment analysis; H&E: hematoxylin and eosin; iTME: immune tumor microenvironment; JSD: Jensen–Shannon divergence; KEGG: Kyoto Encyclopedia of Genes and Genomes; KNN: K-nearest neighbors; LR: ligand–receptor; NK: natural killer; PCC: Pearson correlation coefficient; PPI: protein–protein interaction; RMSE: root mean square error; SCII: spatial cell interaction intensity; scRNAseq: single-cell RNA sequencing; SSIM: structural similarity index; ST: spatial transcriptomic; Teff: T effector; TF: transcription factor.
