# Supplementary material for: StereoSiTE: a framework to spatially and quantitatively profile the cellular neighborhood organized iTME
Source: Gigascience. 2024 Oct 25;13:giae078. doi: 10.1093/gigascience/giae078 (PMC11503478; doi:10.1093/gigascience/giae078)
Supplement: giae078_GIGA-D-23-00276_Revision_1 [file giae078_giga-d-23-00276_revision_1.pdf]

GigaScience

StereoSiTE: A framework to spatially and quantitatively profile the cellular neighborhood organized iTME

--Manuscript Draft--

**Manuscript Number:**	GIGA-D-23-00276R1
**Full Title:**	StereoSiTE: A framework to spatially and quantitatively profile the cellular neighborhood organized iTME
**Article Type:**	Research
**Funding Information:**	
**Abstract:**	Background: With emerging of Spatial Transcriptomics (ST) technology, a powerful algorithmic framework to quantitatively evaluate the active cell-cell interactions in the bio-function associated immune tumor microenvironment (iTME) unit will pave the ways to understand the mechanism underlying tumor biology.<br>Results: This study provides the StereoSiTE incorporating open source bioinformatics tools with the self-developed algorithm, SCII, to dissect a cellular neighborhood (CN) organized iTME based on cellular compositions, and to accurately infer the functional cell-cell communications with quantitatively defined interaction intensity in ST data. We applied StereoSiTE to deeply decode ST data of the xenograft models receiving immunoagonist. Results demonstrated that the neutrophils dominated CN5 might attribute to iTME remodeling after treatment. To be noted, SCII analyzed the spatially resolved interaction intensity inferring a neutrophil leading communication network which was proved to actively function by analysis of Transcriptional Factor Regulon and Protein-Protein Interaction.<br>Conclusions: Altogether, StereoSiTE is a promising framework for ST data to spatially reveal tumoribiology mechanisms.
**Corresponding Author:**	Jiajun Zhang<br>BGI-Research,Chongqing<br>Shenzhen, CHINA
**Corresponding Author Secondary Information:**	
**Corresponding Author's Institution:**	BGI-Research,Chongqing
**Corresponding Author's Secondary Institution:**	
**First Author:**	Xing Liu
**First Author Secondary Information:**	
**Order of Authors:**	Xing Liu
	Chi Qu
	Chuandong Liu
	Na Zhu
	Huaqiang Huang
	Fei Teng
	Caili Huang
	Bingying Luo
	Xuanzhu Liu
	Min Xie
	Feng Xi
	Mei Li

Powered by Editorial Manager® and ProduXion Manager® from Aries Systems Corporation

	Liang Wu
	Yuxiang Li
	Ao Chen
	Xun Xu
	Sha Liao
	Jiajun Zhang
**Order of Authors Secondary Information:**	
**Response to Reviewers:**	(Note: We have prepared a Word document in response to the reviewer's suggestions, which includes replies to all comments along with figures for clarification. This document is provided in the supplementary materials for your review. Please refer to the uploaded supplementary materials for further details. Thank you.)<br><br>Dear editor and reviewers<br><br>First, we (the authors) would like to express our sincere gratitude to the editor and anonymous reviewers for their time and efforts given to our manuscript (Manuscript ID: GIGA-D-23-00276). Thanks a lot, and best regards.<br>Secondly, it is worth pointing out that the reviewers' comments and suggestions have really and constructively helped us improve the quality and presentation of our manuscript much further. Considering their inspiring comments and suggestions, we have duly and carefully revised the manuscript, with the main changes highlighted in red color in the revised manuscript.<br>Thirdly, with many thanks to the reviewers, we would like to address their comments below.<br><br>Authors Response to Comments of Editor<br><br>Comment: In addition, please register any new software application in the bio.tools and SciCrunch.org databases to receive RRID (Research Resource Identification Initiative ID) and biotoolsID identifiers and include these in your manuscript (in the availability section).<br>Response: Many thanks are given to the editor for this comment.<br>We have registered the software application on bio.tools and SciCrunch.org, respectively and added the RRID and biotoolsID to the "Code Availability" section of the revised manuscript on Page 23 (Lines 511-512).<br><br>Authors Response to Comments of Reviewer 1<br><br>Major comments:<br>Comment 1: The authors incorporated several open sources bioinformatics tools. However, how to ensure their combination is the optimal to the spatially resolved cell-cell communication inference performance? For example, cell2location was used to deconvolute cellular composition and construct cellular neighborhood. Why use cell2location for deconvoluting spatial transcriptomics data? Why not use the newest deconvolution algorithms, for example, SpaDecon, Celloscope, POLARIS, GraphST, SPASCER, and EnDecon? No model can adapt to all data. The authors should first verify that cell2location is the best appropriate cell type annotation tool corresponding to iTME. If not, the subsequent analyses will be not appropriate.<br>Response 1: Many thanks are given to the reviewer for the comment.<br>We have compared the performance of Cell2location with other deconvolution algorithms (Celloscope, GraphST, POLARIS) on both STARmap datasets and the stereo-seq dataset of liver cancer. Cell2location showed superior performance in the deconvolution and annotation of cell types. The results figures are presented in Supplementary Figure 1A, B. The detailed description was added to the Results and Methods section of the manuscript on Page 3 (Lines 71-76), Pages 27-28 (Lines 609-644).<br><br>Comment 2: The authors claimed that they computed the decomposition losses of different combinations of the number of CN modules and CT modules. Which combinations? The authors should list them.

Powered by Editorial Manager® and ProduXion Manager® from Aries Systems Corporation

Response 2: We sincerely thank the reviewer for the comment.
The results of various combinations have been displayed in Supplementary Figure 2B. The figure was misreferenced in a previously submitted manuscript. We have corrected this in the revised manuscript on Page 15 (Lines 352-354).

Comment 3: When measuring spatial cell interaction intensity, the authors only simply summed up the ligand and receptor gene expression information of the sender and receiver cells. Why not consider existing classical intercellular communication intensity methods? The authors should compare other intercellular communication intensity measurement methods. Please refer to the following two cites:
Cell-cell communication inference and analysis in the tumour microenvironments from single-cell transcriptomics: data resources and computational strategies, briefings in bioinformatics.
CellDialog: A Computational Framework for Ligand-receptor-mediated Cell-cell Communication Analysis, IEEE Journal of Biomedical and Health Informatics.
Deciphering ligand-receptor-mediated intercellular communication based on ensemble deep learning and the joint scoring strategy from single-cell transcriptomic data, Computers in Biology and Medicine.
Response 3: Thank the reviewer very much for such a comment.
On the one hand, most of the existing classical methods for assessing intercellular communication intensity have not taken spatial information into account, as they were originally designed to analyze single-cell sequencing data, like CellPhoneDB and CellCat. On the other hand, there are some newly published methods for inferring spatial intercellular interactions, such as Giotto, SpaTalk, and CellChat v2. However, these methods have not efficiently utilized spatial information. They simply set a consistent distance threshold without considering different types of interactions or compute N neighboring cells by KNN without considering actual distances. What's more, the amount of stereo-seq data is substantial, often involving millions of cells, which necessitates a significant computational workload. However, most of the published methods were implemented in R and have not been optimized for performance, resulting in the inability to analyze stereo-seq datasets. Instead of integrating gene expression with cell spatial information to infer intercellular communication, SCII optimized computational performance by utilizing matrix calculations to implement functionality. This enables it to process large amounts of data quickly. Despite the aforementioned reasons, we have systematically compared SCII with several other methods for measuring communication intensity (CellphoneDB, CellChat v2, Giotto, SpaTalk) using a small portion of our stereo-seq demo data. SCII showed superior performance compared to other methods. The results are depicted in Figures 3H, I, J. The detailed description was added to the Results and Methods section of the revised manuscript on Pages 10-11 (Lines 241-258) and Page 31 (Lines 717-731).

Comment 4: For protein-protein interaction analysis, the authors queried 628 significant up regulated genes in CN5 area of treatment samples from STRING. Can all obtained proteins be ligands or receptors? In addition, they labeled hub genes and key protein-protein interaction networks, what were these hub genes and key networks used for?
Response 4: Thanks to the reviewer for the comment.
Out of the 628 significantly upregulated genes, 505 proteins were mapped in the STRING database version 11.5. Not all the identified proteins are ligands or receptors. We queried 505 proteins from CellPhoneDB, CellChatDB, and NicheNet ligand-receptor databases, and found that 119 proteins belong to ligands or receptors. The other proteins may interact with proteins within cells that are involved in cellular pathways, such as Irf1 (Interferon regulatory factor 1), which plays a role in regulating IFN and IFN-inducible genes.
We labeled the hub genes and key PPI network in Figure 6G. These hub genes and key networks were used to elucidate the dominant biological protein-protein interactions in the functional iTME unit CN5. The hub genes, including Il1b, Ccl4, Cxcl2, Cxcl1, and Il6, were the responsive downstream targets of NF-kappa B in the context of STING signaling activation, which was well validated in previous publication as follow:

37.Motwani M, Pesiridis S and Fitzgerald KA. DNA sensing by the cGAS-STING pathway in health and disease. Nat Rev Genet. 2019;20 11:657-74. doi:10.1038/s41576-019-0151-1.

Comment 5: Which ligand-receptor pairs could mediate intercellular communication within immune tumor microenvironment? Among these L-R pairs, which L-R pairs are known in existing databases and which L-R pairs are the predicted ones?
Response 5: Thanks to the reviewer for the comment.
In this study, we introduced the existing L-R database from CellChatDB as reference. It integrates signaling molecule interaction data from the KEGG Pathway database and incorporates information from recent experimental studies. Theoretically, all L-R pairs could mediate intercellular communication within the immune tumor microenvironment, and all of them are known in existing databases without any predicted ones.

5.Jin SQ, Guerrero-Juarez CF, Zhang LH, Chang I, Ramos R, Kuan CH, et al. Inference and analysis of cell-cell communication using CellChat. Nat Commun. 2021;12 1 doi:10.1038/s41467-021-21246-9.

Comment 6: "The enrichment analysis of individual CN showed that each CN had a dominant cell type with a spatial aggregation (Fig 2F), which was increasingly obvious than that in whole slide (Fig 2E)." What's a dominant cell type? How to define it?
Response 6: We thank the reviewer for the suggestion.
The dominant cell type was determined based on the neighborhood enrichment matrix, which had the highest neighborhood enrichment score compared to the others. We can clearly identify dominant cell types in neighborhood enrichment heatmaps (Fig 2F) of each CN region. In the CN0 region, the neighborhood enrichment of Teff cells was more pronounced. In the CN1 region, the neighborhood enrichment of M1-like and M2-like cells was stronger than that of other cell types. In the CN5 region, there was a significant neighborhood enrichment of neutrophils, indicating a spatial preference for neutrophil aggregation in CN5.

Comment 7: "To reduce the variance among open-sourced L-R databases, we unified L-R database in SCII by choosing L-R dataset in CellChatDB, which assigned each L-R with an interaction distance associated classification as secreted signaling, ECM receptor and cell-cell contact." How to unify L-R database? Did it allow for user-specified LR databases and/or add user-specified LR databases?
Response 7: Many thanks are given to the reviewer for this comment.
Considering difference communication types (secreted signaling, ECM receptor and cell-cell contact) associated to different active distance, we recommend L-R dataset in CellChatDB for its clear classification (revised sentence in Pages 9 (Lines 202-205)). Also, the selection of databases is flexible and customer defined. It allows users to introduce LR databases of interest. The database file should be formatted in CSV format and contains columns ‘source' and ‘target', which indicate the ligand and receptor. If users want to process different types of LR with different strategies, the column 'annotation' indicating LR types is required. The SOP for “user defined database” was revised in the Methods section of the revised manuscript on Page 30 (Lines 704-710).

Comment 8: In figure 3, how to confirm which L-R pairs mediate intercellular communication?
Response 8: Many thanks are given to the reviewer for this comment.
Every intercellular communication inferred by SCII contains two values: spatial cell interaction intensity and p-value. We defined communications with p-values < 0.05 as significant and considered them as L-R pairs mediating intercellular communication. To be noted, the threshold is flexible. The user can set a threshold for intensity to filter out interactions of interest.

Comment 9: StereoSiTE is composed of multiple modules, is it scalable? Can some of these modules (such as clustering and cell type annotation) be replaced with other more powerful modules?

Powered by Editorial Manager® and ProduXion Manager® from Aries Systems Corporation

Response 9: Sincerely thank the reviewer for the comment.
StereoSiTE is a modular software implemented as a Python package (https://github.com/STOmics/stereosite, https://pypi.org/project/stereosite/). Each module can be called separately to analyze the data. Therefore, it is scalable; any module can be replaced with more powerful alternatives.

Comment 10: The authors claimed that "CellPhoneDB detected many false positive interactions". How to find these false positive LRIs? How to validate the LRIs be false positives? Please list the found false positive LRIs.
Response 10: We thank the reviewer for the suggestion.
We identified the intercellular interactions inferred by CellPhoneDB but not by SCII and classified them as false positive LRIs. To validate these LRIs as false positives, we calculated their median cell distance and compared it to the median cell distance of the interactions inferred by both CellPhoneDB and SCII. The results in Figure 3D indicate that false positive LRIs had a longer median cell distance compared to the others. Besides, we selected several representative false positive LRIs and showed their spatial distribution and permutation test results in Figure 3G. They are clustered into two categories: 1) interactions that exhibit spatial co-expression between ligand and receptor but lack significance in spatial proximity with high p-values. 2) interactions show no spatial co-expression between ligands and receptors. The false positive LRIs found have been listed in Supplementary Table 1. A more detailed description can be found in the revised manuscript on Pages 9-10 (Lines 212-237).

Comment 11: In Figure 3, the authors should add comparison experiments between StereoSiTME and classical intercellular communication analysis tools.
Response 11: Thank the reviewer for such a comment.
We have compared the performance of SCII with other intercellular communication analysis tools (CellPhonDB, CellChat v2, Giotto, Spatalk). In the study, SCII demonstrated superior performance compared to other methods. The result figures are shown in Figure 3H, I, J. The detailed description was added to the Results and Methods section of the revised manuscript on Pages 10-11 (Lines 241-258) and Page 31 (Lines 717-731).

Minor Comments:
Comment 1: The text in subfigure A, B, and C in Supplementary Figure 2 is obscure. The authors should revise Supplementary Figure 2.
Response 1: We thank the reviewer for the suggestion.
We have revised Supplementary Figure 2, which has been renamed to Figure 3 as recommended. The figure can be found in the revised manuscript on Page 37.

Comment 2: In Section "Abstract", iTME should use full name when it first appears.
Response 2: We thank the reviewer for the suggestion.
We have added the full name in the section abstract of the revised manuscript on Page 1 (Line 22).

Comment 3: Which cites of "13 Li, M. et al. (2023)." is in the reference list?
Response 3: We thank the reviewer for the suggestion.
We have corrected the cite of "13 Li, M. et al. (2023)." in the reference list, which is on Page 39 (Lines 866-868).

22.Li M, Liu H, Li M, Fang S, Kang Q, Zhang J, et al. StereoCell enables high accuracy single cell segmentation for spatial transcriptomic dataset. bioRxiv. 2023:2023.02.28.530414. doi:10.1101/2023.02.28.530414.

Authors Response to Comments of Reviewer 2
Comment: Although the authors performed comprehensive works to demonstrate the potential applications of StereoSiTE. This reviewer has strong concerns about the potential novelty and effectiveness of StereoSiTE over existing methods. The CN results were not mindful of the spatial information, and the labeled cellular

neighborhood (CN) may mislead users. Additionally, although the L-R pair could be categorized into three classifications based on interaction distance, the SCII only uses different radius to infer cell communication without employing a different strategy for predicting interactions in distinct L-R pairs.
Response: Sincerely thank the reviewer for the comment.

The concept of cellular neighborhood has been first proposed to analyze multi-fluorescence data. This is a well-recognized concept to decode the TME with cellular identification. The design of Stereosite framework to address the question of “how iTME respond and function under stimulation” with stereo-seq data. Therefore, the composition in iTME community is critical for downstream analysis. We believe the novelties of our work are: 1. A self-developing algorithm called SCII to quantitively calculate the interaction intensity by introducing spatial information; 2. Providing flexible and user friendly framework to analysis spatially resolved data at single cell resolution. Although CN does not utilize spatial information, it is capable of identifying tissue domains. CN showed good performance compared to other tissue domain division methods (BANKSY, Giotto HMRF). The results have been added to the Results section of the revised manuscript on Page 7 (Lines 170-177). In the current framework, the CN is designed to identify functional domain. Afterwards, cellular interaction within CN of interest will be decoded by SCII with full consideration of spatial information. The identification of domain should correspond to the research design; therefore, our framework allows replacement of CN module by other method. Meanwhile, we are planning to integrate spatial information into the CN analysis to take spatial pattern into consideration.
Considering the comment regarding the strategy for predicting interactions in distinct L-R pairs, we have updated the SCII function of the StereoSiTE software. Different strategies for predicting interactions in distinct L-R pair types have been implemented. Users can specify different radius thresholds for distinct L-R types using parameters, for example: {'Secreted Signaling': 100, 'ECM-Receptor': 100, 'Cell-Cell Contact': 30}. The new version of StereoSiTE has been updated on GitHub and PyPI (https://github.com/STOmics/stereosite, https://pypi.org/project/stereosite/). At the same time, we have updated the Results section of the revised manuscript on Pages 9-10 (Lines 202-240), and Figure 3A-G.

Comments:
Comment 1: The authors fail to show the novelty and advantages of CN compared to other methods, such as DeepST, which integrates gene expression, spatial location and image information. The authors should provide the comparison with the several recent strategies that consider the effect of local niches including BANKSY, stLearn, Giott, and DeepST.
Response 1: Sincerely thank the reviewer for the comment.
We have compared CN with other methods. The results demonstrate that the CN method exhibits nearly consistent performance with BANSKY, while outperforming Giotto HMRF on the benchmark dataset STARmap. The detailed description has been added to the Results section of the revised manuscript on Page 7 (Lines 170-177). Because stLearn and DeepST require H&E images, which were not included in our dataset, we have not been able to conduct a comparison with them.

Comment 2: The authors should compare SCII with additional methods such as CellPhoneDB v3 and Cellchat v2, demonstrating its superior performance.
Response 2: Thank the reviewer for such a comment.
We have compared SCII with additional methods (CellPhonDB v4, CellCgat v2, Giotto, Spatalk) using part of our stereo-seq demo data. SCII showed superior performance compared to other methods. The results are depicted in Figure 3H, I, J. The detailed description was added to the Results and Methods section of the revised manuscript on Pages 10-11 (Lines 241-258) and Page 31 (Lines 717-731).

Comment 3: The method used for cell segmentation should offer more comprehensive

information rather than solely citing "Li, M. et al. (2023)".
Response 3: Many thanks are given to the reviewer for raising the comment.
We have added comprehensive information about the cell segmentation method in the method section of the revised manuscript on Page 26 (Lines 573-581).

Comment 4: Format of the paper. The alignment inconsistency within the manuscript—with some paragraphs centered and others justified—should be corrected for uniformity.
Response 4: Sincerely thank the reviewer for the comment.
We have corrected the format of the revised manuscript.

Comment 5: The figures and manuscript containing 'Teff' and 'M2-like' cell types should provide a legend explaining the abbreviations for clarity.
Response 5: Sincerely thank the reviewer for the comment.
We have provided a legend explaining the abbreviations for clarity in the legend of figure 2 on Page 8 (Lines 191-193).

Comment 6: The font size of the labels in Figures 5E-F is insufficient for easy reading and should be enlarged.
Response 6: Sincerely thank the reviewer for the comment.
We readjusted the font size of Figures 5E-F for easy reading. The figure can be found in the revised manuscript on Page 17.

Authors Response to Comments of Reviewer 3
Comment 1: The paper's objective is commendable, and the overall organization of the content, along with the obtained results, holds great promise. Nevertheless, certain aspects need to be addressed. The proposed approach's novelty is significantly anchored in the SCII software. However, the paper has notable drawbacks. It falls short in providing a theoretical and scientific comparison with other similar tools. Moreover, the comparison includes systems that do not incorporate spatial considerations, posing a limitation in assessing the method's uniqueness in a broader context.
Response 1: Sincerely thank the reviewer for the comment.
We have systematically compared SCII with several other intercellular communication measurement methods (CellPhoneDB v4, CellChat v2, Giotto, Spatalk). Considering the limited computational efficiency, we selected part of the demo data containing 11,214 cells for comparison. We calculated the co-expression percentage of interactions between sender and receiver cells inferred by distinct methods. We hypothesized that a higher co-expression percentage indicates more reliable inference. Interactions inferred by SCII showed the highest level of co-expression, indicating the superior performance of SCII in measuring spatial cell-cell communication. The results have been updated in Figures 3H, I, J, and on Pages 10-11 (Lines 241-258). The method has been added on Page 31 (Lines 717-731).

Comment 2: Give more details on which systems are you referring here: "To improve accuracy, we recommended using spatially resolved data at single cell resolution". Please provide your insights on the rationale for employing or abstaining from downstream analysis to comprehend the spatial distribution of gene expression in tissue, as https://doi.org/10.1093/gigascience/giac075 and https://doi.org/10.1038/s41467-023-36796-3. Additionally, consider discussing how this is associated with the prediction, validation of the functional enrichment or on step: Clustering bins into different cellular neighborhoods based on their cellular composition.
Response 2: Sincerely thank the reviewer for the comment.
We have given more details about the single cell resolution systems mentioned in the paper in the Results section of the revised manuscript on Page 4 (Lines 93-95). Compared with methods like GraphST and Stardust, which achieve tissue domain division by utilizing gene expression matrix and genes' spatial distribution, tissue domain division based on cellular composition (CN) offers a more detailed and accurate understanding of tissue biology, cellular function, and disease mechanisms.

Powered by Editorial Manager® and ProduXion Manager® from Aries Systems Corporation

	1. It allows for a more accurate identification of specific cell types present within a tissue. This can help in understanding the functional roles and characteristics of individual cell types within a tissue, providing a more detailed insight into tissue function. 2. By focusing on cellular composition, tissue domain division allows for the analysis of individual cell types within a tissue. This can help uncover specific cellular functions, interactions, and pathways that may be relevant to tissue function or disease processes. 3. Tissue domain division based on cellular composition can provide insights into disease mechanisms at the cellular level. By studying specific cell types implicated in a disease, researchers can better understand the underlying cellular processes and develop targeted therapies.<br>Tissue domain division by CN can help us focus on specific regions with an enrichment of the cell types of interest. As described in our manuscript, we next analyzed the intercellular communications in the specific CN region and found some biologically meaningful interactions. Theoretically, after the interaction between the ligand expressed by sender cells and the receptor expressed by receiver cells, downstream transcriptome factors and corresponding pathways in receiver cells will be activated. Therefore, we analyzed the transcription factor activities and pathway enrichment in the specific CN region.<br><br>Finally, we (the authors) would like to express thanks again sincerely to the editor and anonymous reviewers for their time and efforts spent in handing the manuscript, as well as providing us many constructive comments for improving further the presentation and quality of this manuscript.<br><br>Sincerely,<br><br>Xing LIU<br><br>(On behalf of all authors)<br><br>11 Apr 2024
**Additional Information:**	
**Question**	**Response**
Are you submitting this manuscript to a special series or article collection?	No
**Experimental design and statistics**<br><br>Full details of the experimental design and statistical methods used should be given in the Methods section, as detailed in our Minimum Standards Reporting Checklist. Information essential to interpreting the data presented should be made available in the figure legends.<br><br>Have you included all the information requested in your manuscript?	Yes
**Resources**	Yes

A description of all resources used, including antibodies, cell lines, animals and software tools, with enough information to allow them to be uniquely identified, should be included in the Methods section. Authors are strongly encouraged to cite Research Resource Identifiers (RRIDs) for antibodies, model organisms and tools, where possible.<br><br>Have you included the information requested as detailed in our Minimum Standards Reporting Checklist?	
**Availability of data and materials**<br><br>All datasets and code on which the conclusions of the paper rely must be either included in your submission or deposited in publicly available repositories (where available and ethically appropriate), referencing such data using a unique identifier in the references and in the "Availability of Data and Materials" section of your manuscript.<br><br>Have you have met the above requirement as detailed in our Minimum Standards Reporting Checklist?	Yes

Powered by Editorial Manager® and ProduXion Manager® from Aries Systems Corporation

Manuscript

Click here to access/download;Manuscript;Revised Submission Final Version-2024.docx

StereoSiTE: A framework to spatially and quantitatively profile the cellular neighborhood organized iTME

Xing Liu[1,2*], Chi Qu[1,2,3*], Chuandong Liu[1,2*], Na Zhu[2], Huaqiang Huang[1,2], Fei Teng[2], Caili Huang[2], Bingying Luo[1], Xuanzhu Liu[2], Min Xie[1,2,3], Feng Xi[1,2,3], Mei Li[2], Liang Wu[1,2,3], Yuxiang Li[2], Ao Chen[1,2,3#], Xun Xu[1,2,3#], Sha Liao[1,2,3#], Jiajun Zhang[1,2,3#]

[1.] BGI Research, Chongqing, 401329, PR China

[2.] BGI Research, Shenzhen, 518083, PR China

[3.] JFL-BGI STOmics Center, Jinfeng Laboratory, Chongqing 401329, China

*： Theses authors contributed equally to this study.

#: Co-corresponding author

Correspondence to:

Dr. Jiajun ZHANG

BGI-Shenzhen, Shenzhen 518083, China

Email: zhangjiajun1@genomics.cn

Abstract

Background: With emerging of Spatial Transcriptomics (ST) technology, a powerful algorithmic framework to quantitatively evaluate the active cell-cell interactions in the bio-function associated immune tumor microenvironment (iTME) unit will pave the ways to understand the mechanism underlying tumor biology.

Results: This study provides the StereoSiTE incorporating open source bioinformatics tools with the self-developed algorithm, SCII, to dissect a cellular neighborhood (CN) organized iTME based on cellular compositions, and to accurately infer the functional cell-cell communications with quantitatively defined interaction intensity in ST data. We applied StereoSiTE to deeply decode ST data of the xenograft models receiving immunoagonist. Results demonstrated that the neutrophils dominated CN5 might

attribute to iTME remodeling after treatment. To be noted, SCII analyzed the spatially resolved interaction intensity inferring a neutrophil leading communication network which was proved to actively function by analysis of Transcriptional Factor Regulon and Protein-Protein Interaction.

Conclusions: Altogether, StereoSiTE is a promising framework for ST data to spatially reveal tumoribiology mechanisms.

Introduction:

Cell-cell communication by molecular interaction within immune tumor microenvironment (iTME) is closely related to tumorigenesis, progression, and treatment response, which provides important targets for diagnosis, prognosis, disease monitoring, drug design, etc[1]. The advanced spatial transcriptomic (ST) technology provides opportunities to better elucidate physiological and pathological progress by observing the comprehensive nature of transcriptome in spatial[2, 3]. The expression profile with spatial coordination empowers the ability to reveal the landscape of iTME and to dissect the cell-cell communication in pathogenesis associated iTME region[3]. However, how to precisely find pathogenesis associated iTME, quantitatively infer cell-cell communications by reasonably utilizing spatial information, remain to be the significant challenges. Most open source software for cell-cell communication analysis are designed based on either gene expression[4, 5] or biological distance, without considering the interactable cell-cell distance[6-8]. Comprehensively understanding the importance of spatial information needs a well-designed framework composed of powerful algorithms to decode spatial data.

Here, we present StereoSiTE, an analytical framework for comprehensive depiction of landscape of iTME which is defined by Cellular Neighborhood (CN)[9] and dissection of spatial cell interaction intensity (SCII). CNs are defined by the cellular composition obtained from cell type deconvolution result and SCII next inferred cell-cell communication using both cell graph network constructed by cell coordinates and targeted L-R expression. Moreover, analysis of SCII in CN region of

interest can elucidate multicellular communities in correspondent iTME unit, which provides new dimension in evaluating iTME. To exhibit the application scenario, we applied StereoSiTE to analyze ST data of xenograft models receiving immunoagonist treatment and revealed the iTME landscape and spatial cell interactions in functional iTME units, which provided molecular interaction and sequential activities of tumor in response to treatment.

Result

StereoSiTE: A novel framework to spatially and quantitatively profile cell-cell communications in cellular neighborhood organized iTME

In this framework (Fig 1A), we first performed cell type deconvolution for squared bin data (each bin contains more than one cell) and cell type annotation for cell bin data by integrating the single-cell sequencing data. Cell2location, which demonstrated superior performance compared to other deconvolution methods (Celloscope[10], GraphST[11], POLARIS[12]) on both STARmap[13] datasets (Supplementary Fig 1A) and the stereo-seq dataset of liver cancer (Supplementary Fig 1B), was selected to perform these tasks. Absolutely, the results generated by other deconvolution methods can be used to conduct the following CN and SCII analysis. Next, analyze the cellular neighborhood to investigate the tissue domain based on the cellular composition, which is critical to iTME organization. Then, the self-developed SCII was applied to analyze spatially resolved cell-cell communication, indicating key molecular activities by constructing a biological network.

Cellular Neighborhood (CN) analysis was performed on squared bin data based on cellular composition resolved through the deconvolution method[14] (cell2location) using single-cell sequencing data as a reference. Squared bins with similar cellular composition were clustered together using Leiden[15]. Each cluster can be defined as a type of iTME constructed by a specific cellular composition. To select CNs of interest for functional analysis, we integrated a matrix that concurrently covers cellular

neighborhoods (CNs) and cell types (CTs) and introduced Tensor[16] to decompose the module matrix.

To analyze the spatially informed cell-cell interaction, we developed Spatial Cell Interaction Intensity (SCII). SCII detected actively functional LR pairs by quantitatively defining interactive intensity based on spatial proximity of cells and the expression of genes. To improve accuracy, we recommend using spatially resolved data at the single-cell resolution, which can be generated by some sequencing-based method Stereo-seq[17], Seq-Scope[18], and imaging-based methods MERFISH[19], seqFISH[20], STARMap[21]. To get the expression matrix at single-cell resolution from the original Stereo-seq data which was analyzed in our research, the StereoCell cell segmentation algorithm, utilizing a deep neural network approach[22], was employed to produce single cell masks based on nuclear staining images. Subsequently, these single cell masks were combined with spatial expression matrix to derive the single-cell resolution spatial expression profile. Then we conducted cell type annotation using cell2location referring to the protocol mentioned above. With the annotated single-cell resolved data, we constructed the cell graph network by connecting cells within a radius threshold and revised it by assigning the weight to connected edges based on ligand-receptor co-expression levels. Edges where the end nodes, representing sender and receiver cells, had no ligand or receptor expression were filtered out. Then, we calculated the local cell interaction intensity of each sender cell with its surrounding receiver cells by summing up the weight of edges linked to it. The overall interaction intensity of the entire slide was determined by summing up the weight of all edges. Finally, to measure the significance of the interaction, we used the permutation test to generate a null distribution by shuffling cell type annotation labels, and the p-value can be calculated from this null distribution (Fig 1C).

To demonstrate that testable hypotheses can be derived from inferred cell-cell communications, both upstream and downstream signaling activities must be comprehensively analyzed. StereoSiTE also included analysis of Differentially Expressed Genes (DEG), Protein-Protein Interaction (PPI) network, and

Transcriptional Regulatory Factory (TF) network to provide an end-to-end solution for selected L-R pairs induced molecular mechanisms of specific iTME units.

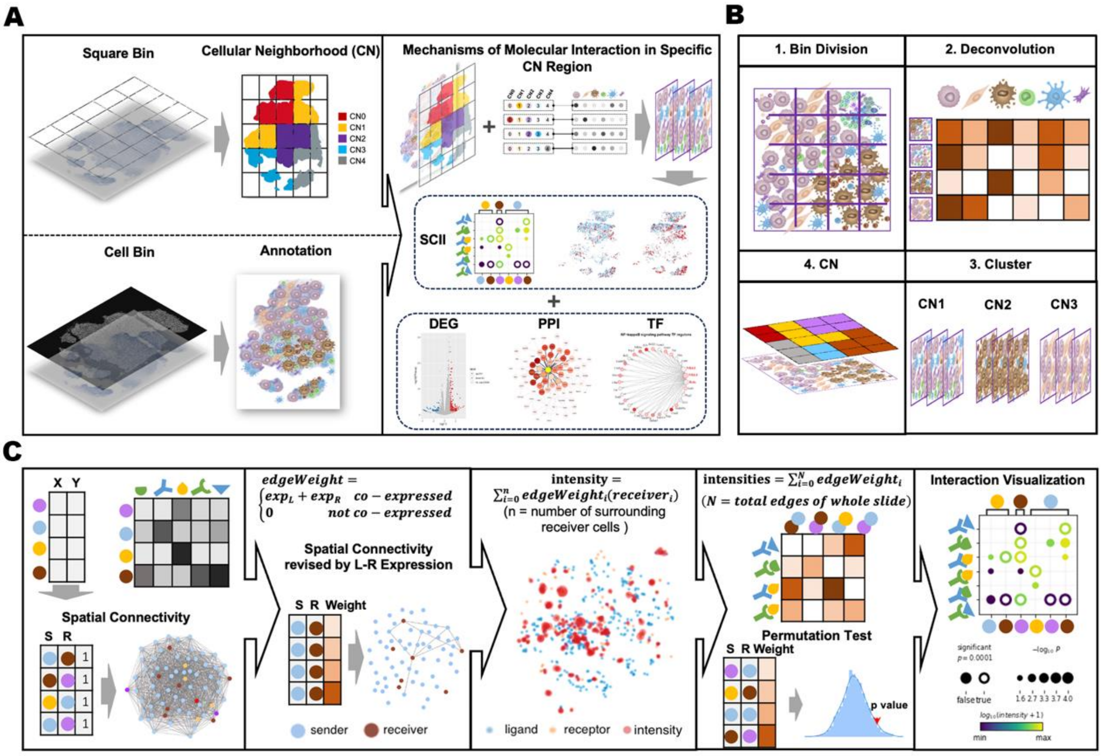

Figure 1. Schematic diagram of the StereoSiTE workflow.

A. Overview of StereoSiTE, including analysis of CN, annotation, tensor deconvolution, SCII, DGE, pathway enrichment, and protein-protein interaction. **B.** Conceptual diagram of CN: 1. Converting the spatial gene expression matrix into a binned data matrix. 2. Resolving the cellular composition of each bin through deconvolution (cell2location). 3. Clustering bins into different cellular neighborhoods based on their cellular composition. 4. Mapping the CNs in situ. **C.** Principle of SCII: Firstly, constructed the cell graph network based on coordinates of cells by connecting cells with their surrounding cells within a certain distance; Secondly, revised the cell graph network by assigning the connected edges with weight counting from the corresponding expression of ligand-receptor genes, removing the connections without LR co-expression; Thirdly, calculated the locally interaction intensity between each sender cell with their surrounding receiver cells and showed their spatial distribution; Then, calculated the interaction intensity between any two cell types of the whole slide,

permutation test was used to build a null distribution by shuffling the cell type labels and computed the p value. Finally, the spatial cell interaction intensities and p-values were visualized using bubble plots indicating interaction intensity (by color) and p-value (by size).

Decoding iTME into cellular neighborhood (CN) organized units

To verify that CN is a reasonable unit to study iTME, we applied this framework to analyze representative Stereo-seq data from the cancer tissue of a xenograft model and analyzed CN-based iTME units. We generated a binned data matrix with a bin size of 100 (equivalent to 50µm*50µm) and decoded the cell-type composition of each bin. The ST data was clustered into 7 different CN clusters (Fig 2A), each with a unique cell-type composition (Fig 2B). To decode the microenvironment of each CN, we aligned annotated data at single-cell resolution on clustered CNs under the guidance of coordinates. Fig 2C shows the spatial distribution of annotated cells, and the proportion of different cell types in this sample was calculated (Fig 2D). Overall, non-immune cells accounted for 77.05%, lymphoid cells accounted for approximately 10.28%, and myeloid cells accounted for approximately 12.67%.

To confirm that outstanding spatial features can be identified by CN, we quantified the spatial aggregation between any two annotated cell types using neighborhood enrichment[23]. We observed the aggregation of various cell types, such as T cells, macrophages, and non-immune cells, throughout the entire slide (Fig 2E). Based on this result, it is difficult to define the critical cell types that distinguish the sample from others. The enrichment analysis of individual CN showed that each CN had a dominant cell type with spatial aggregation (Fig 2F), which was more pronounced than that in the whole slide (Fig 2E). In the CN0 region, the neighborhood enrichment of Teff cells was more pronounced compared to other regions. This enrichment was noticeable throughout the entire slide, although it was not clearly distinguishable due to interference from signals of other cell types. This might result from a small portion of

Teff cells and a predominance of other immune cells on the entire slide, while Teff cells were dominant in CN0. In the CN1 region, the neighborhood enrichment of M1-like and M2-like cells was stronger than that of other cells. Compared with the results of the entire slide, we can clearly see the dominant aggregation of macrophages in CN1. In the CN5 region, there was a significant neighborhood enrichment of neutrophils, indicating a spatial preference for neutrophil aggregation in CN5. Therefore, CN analysis can help divide the tissue region into different tissue domains corresponding to specific iTME. It was highly risky to overlook important candidates for in-depth analysis without identifying the CN of interest before conducting molecular analysis.

Considering there are some other published tissue domain division methods, we compared the performance of our CN methods with them (BANKSY[24], Giotto HMRF[8]) on the benchmark dataset STARmap. The comparison results demonstrate that the CN method shows nearly consistent performance with BANSKY, while it is much better than Giotto HMRF (Supplementary Fig 1C, D). Taking into account the comparison results of BANSKY with other methods (GraphST[11], SpaGCN[25], SpiceMix[26], STAGATE[27], BayesSpace[28]) in its original research, we believe that the CN method is competent for tissue region division.

In the following section, we would like to demonstrate the key module of StereoSiTE, SCII, which was used to decoding the spatial intercellular interaction in specific iTME region.

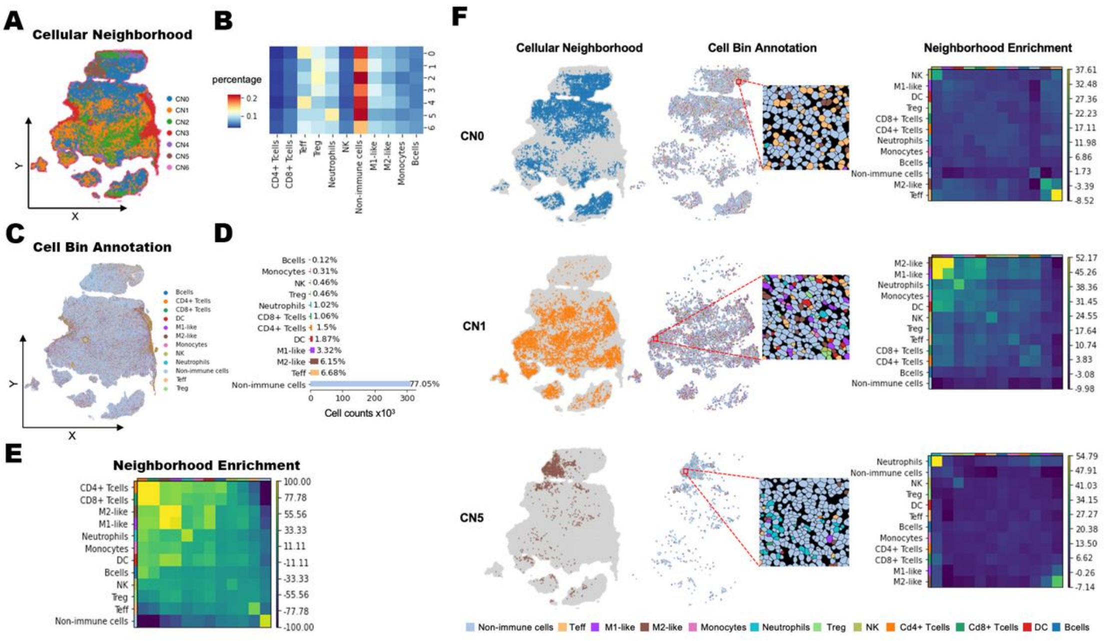

Figure 2. The Performance of Cellular Neighborhood (CN) in decoding iTME.

A. The spatial distribution of CN. **B.** Heatmap indicated the cellular composition in each CN region. **C.** Spatial distribution of cell type annotation at single-cell resolution. Each spot represented a single cell. **D.** The counts and percentages of each cell type, the X-axis represented the cell number. **E.** The heatmap showed the neighborhood enrichment between any two cell types. The radius threshold was set to be 50 μm. **F.** Spatial distribution of representative CNs (CN0, CN1, CN5) (left). The corresponding cell distribution was shown in the middle, with a zoomed-in region displayed for clearer observation. Neighborhood enrichment between any two cell types in regions of CN0, CN1, and CN5 was shown on the right. Teff: T effector cell, Treg: T regulatory cell, M1-like: M1-type macrophage, M2-like: M2-type macrophage, NK: Nature Killer cell, DC: Dendritic Cell.

Spatial Cell Interaction Intensity (SCII): Inferring spatial cell-cell communications

The association of cellular distance with cell-cell communication is proposed by stimulated experiments and computational calculation[29]. To validate the impact of distance on communicative activities and establish recommended parameters, we

conducted an evaluation procedure to compare results from SCII at various distance thresholds.

Considering the variance among open-source LR databases, we recommend LR datasets from CellChatDB, which assigned each LR with an interaction distance associated classification as secreted signaling, ECM receptor and cell-cell contact, for SCII analysis. Fig 3A demonstrated the count and overlap of cell-cell communications inferred by SCII at radius of 30μm, 100μm, and 200μm for all types of LR pairs, and at a radius of 30μm for cell-cell contact LR pairs while at a radius of 200μm for secreted signaling and ECM receptor pairs. It also showed the communication inferred by CellPhoneDB disregarding cell spatial distribution in the CN0 region, which comprised 131013 cells. Fig 3B demonstrated the association between these results. The proportion of interaction types at various distance thresholds was illustrated in Fig 3C. To be noted, the inferred LR results had limited overlap between SCII and CellPhoneDB despite of distance thresholds. We calculated the median cell distance of communications inferred by CellPhoneDB alone and by both CellPhoneDB and SCII (Fig 3C). The distances between neighboring cell pairs involved in communications exclusively predicted by CellPhoneDB were significantly longer than others (Fig 3D), indicating limited reachable interaction. In other words, CellPhoneDB inferred many false positive interactions, which could be avoided in SCII by including a distance threshold in the analysis. The false positive interactions were listed in Supplementary Table 2.

Fig 3E showed representative LR pairs identified by CellPhoneDB, while Fig 3F showed those identified SCII. In Fig 3G, we mapped the intensity of these inferred cell-cell communications in situ, which included three categories: 1. inferred by CellPhoneDB alone, 2. by both CellPhoneDB and SCII, and 3. by SCII alone. Interactions inferred by CellPhoneDB alone can be further clustered into two categories (Fig 3G): 1) interactions that exhibit spatial co-expression between ligand and receptor but lack significance in spatial proximity with high p-values, such as interactions mediated by Spp1-Cd44 between non-immune cells and Teff, M1-like, and DC. 2)

interactions show no spatial co-expression between ligands and receptors, such as interactions mediated by App-Cd74 between M1-like, M2-like, and NK with monocytes. The interaction induced by App and Cd74[30] requires direct contact between sender and receiver cells. This demonstrates that the introduction of a distance threshold could prevent false positives caused by unreachable cells. Fig 3G also showed communications inferred by both CellPhoneDB and SCII, such as between non-immune cells, M2-like cells, and Teff cells mediated by Ccl8-Ccr5 and App-Cd74. Additionally, communications exclusively inferred by SCII were observed between M2-like cells, non-immune cells, and Teff cells mediated by Ccr8-Ccr1 and H2-D1-Cd8a, with a high intensity of interaction and significance (Supplementary Fig 1E, F). This supports the superior accuracy of SCII over methods without considering spatial information.

Instead of comparing SCII solely with one classical method that ignores spatial information, we systematically compared SCII with several other communication measurement methods that consider spatial information. These methods include CellChat v2[31], Giotto with its spatCellCellcom function[8], and SpaTalk[6]. Considering the limited computational efficiency and the inability to process large datasets of other methods, we selected a small portion of the demo data containing 11,214 cells for the following analysis (Supplementary Fig 1G). To standardize the LR databases across different methods, we selected the LR pairs that exist in both CellPhoneDB and CellChatDB[5]. Considering that some methods cannot handle LR pairs with complexes, we filtered out protein complexes, resulting in 441 LR pairs for the following analysis. The intersections of interactions generated from different methods were displayed in Fig 3H and Supplementary Fig 1H, demonstrating that they varied from each other with low overlap. The interactions inferred by SCII had more overlap with CellPhoneDB and CellChatDB than with Giotto and SpaTalk. We reasoned that a higher co-expression percentage of the inferred interactions between sender and receiver cells indicates more reliable inference. Interactions inferred by SCII

showed the highest co-expression level compared with other methods, indicating the superior performance of SCII in measuring spatial cell-cell communication.

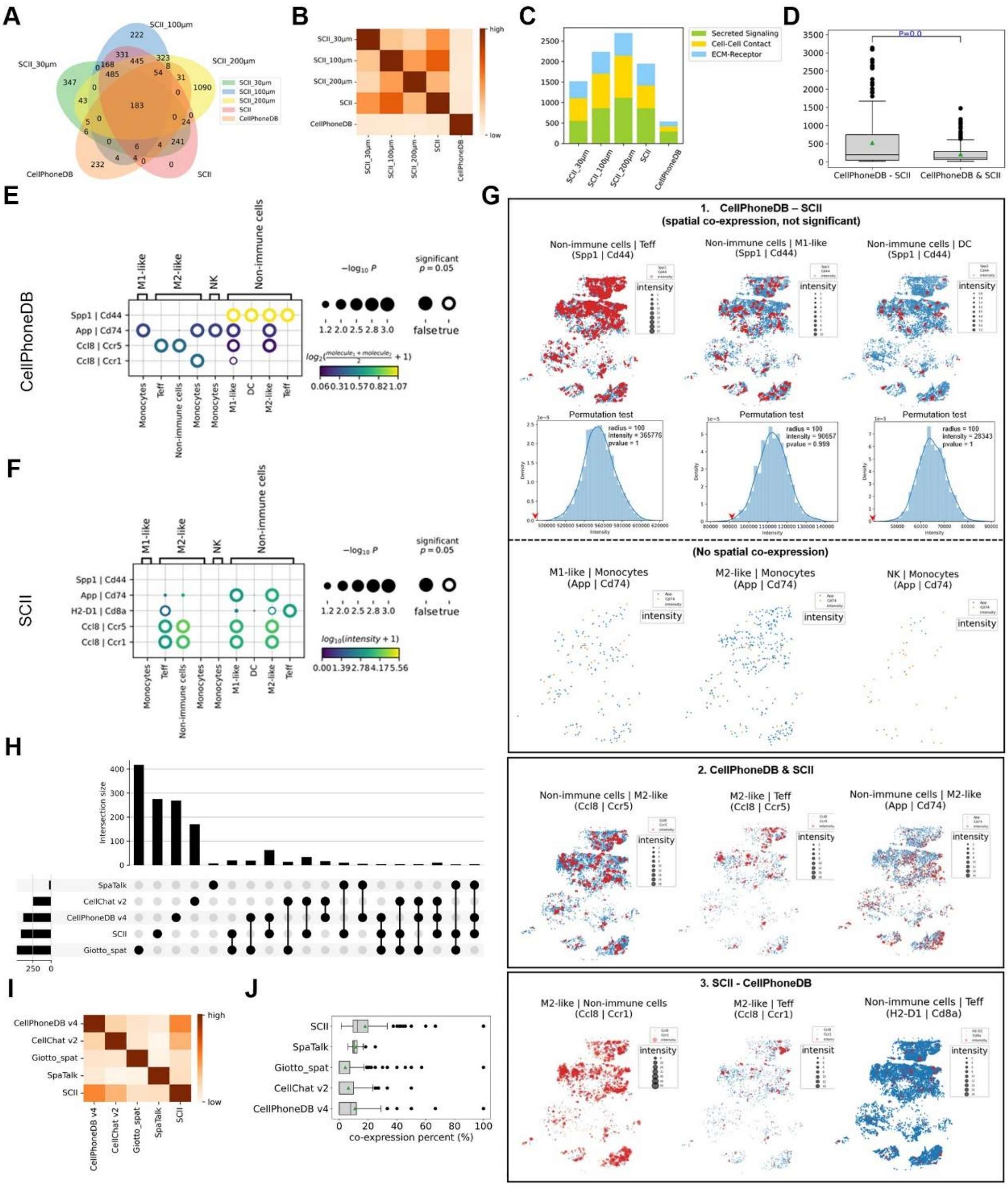

Figure 3. Superior performance of Spatial Cell Interaction Intensity (SCII) compared with method ignoring spatial information and other cell-cell interaction inference methods.

A. Venn diagram illustrated the intersection and variance in inferred interactions from SCII with radius thresholds of all=30μm, all=100μm, all=200μm, 'Secreted

Signaling=100, ECM-Receptor=100, Cell-Cell Contact=30', and from CellPhoneDB. Label SCII indicated the results of various radius thresholds for LR pairs in different types. **B.** Jaccard index between any two interaction results. **C.** Stacked bar plots show the number of predictive interactions of different types detected by SCII at radius thresholds of 30 µm, 100 µm, and 200 µm, as well as various radius thresholds, and by CellPhoneDB. Green indicates secreted signaling, yellow indicates cell-cell contact, and blue indicates ECM-receptor. **D.** For each communication, we connected the sender cell with its N surrounding receiver cells to construct a cell graph network using KNN. We then calculated the median distance of all connected cell pairs. We calculated the median cell distance quantile of communications inferred by CellPhoneDB alone and by both CellPhoneDB and SCII. Shown is an approx two-sided P value from Wilcoxon rank-sum test. The green triangle indicates mean value. **E.** The representative results inferred by CellPhoneDB. The color of the bubble indicates the average expression of the LR pairs, while the size indicates confidence. The confidence p-value less than 0.05 was indicated by a circle and represents that the interaction was significant. **F.** The representative results inferred by SCII. The color of the bubble indicates the intensity strength, while the size represents the same as D. **G.** Spatial distribution of communications between sender cells and receiver cells mediated by specific ligand-receptor. 1. CellPhoneDB - SCII: Interactions detected by CellPhoneDB alone but not by SCII can be divided into two categories: spatial co-expression but not significant. The spatial distribution of these interactions was shown, and the corresponding null distribution of permutation tests was displayed at the bottom. Redarrows indicated the actual intensity values. For interactions with no spatial co-expression, the spatial distribution was shown, but there was no cell spatial interaction intensity. 2. CellPhoneDB & SCII: Spatial distribution of interactions inferred by both CellPhoneDB and SCII. 3. SCII – CellPhoneDB: Spatial distribution of interactions inferred by SCII alone. Blue spots indicate sender cells expressing the ligand gene, orange spots indicate receiver cells expressing thereceptor gene, and red spots indicated local interaction intensity. **H.** Upset plot displayed the intersection of interactions

inferred by SCII and other methods for measuring intercellular communication (CellPhoneDB v4, CellChat v2, Giotto, SpaTalk). **I.** Jaccard index indicates the interactions inferred by any two methods. **J.** Performance comparison of SCII with other methods for inferring intercellular interactions. The box plots display the co-expression percentage of interactions inferred by different methods. The green triangle indicates the mean value.

Profiling tumor microenvironment using spatial transcriptomics

To apply the designed framework to address iTME associated research questions, we introduced xenograft models (Fig 4A) with immune agonist (STING agonist) treatment[32]. Here, we collected spatially resolved transcriptomic data from xenograft tumor tissues by Stereo-seq, a spatial sequencing technology with the subcellular resolution of 500nm[17]**.** Data matrix at resolution of single cell was obtained after cell segmentation processing based on nuclear staining[22]. By cell2location induced deconvolution of spatial transcriptomic matrix with reference previously reported[33] we identified and validated 12 distinct cell types (Fig 4B), including 6 of lymphoid-lineage, 5 of myeloid-lineage and 1 of non-immune cluster. With proportion of cell types across samples (Fig 4C), we noticed the different compositions of immune cells across samples. Further comparison in quantitative analysis indicated less cell numbers in treatment group when compared to control (Fig 4D). It was proposed that necrosis caused by treatment might attribute to reducing cell numbers in treatment group (Fig 4E). Notably, control groups had higher frequency of M2-like macrophages, meanwhile, treatment groups possessed higher frequencies of neutrophils (Fig 4C). Interestingly, we additionally observed a location preference (Fig 4F) of neutrophils in treatment group, where they tended to cluster around necrosis niches, vice versa, other cells like M2-like macrophages in control group were randomly distributed. However, methods interrogating the correlation between specific bioactivities and the corresponding spatial preference were rarely exploited. To validate

our hypothesis of spatial preference between different cell types, we applied analysis of CN in following section.

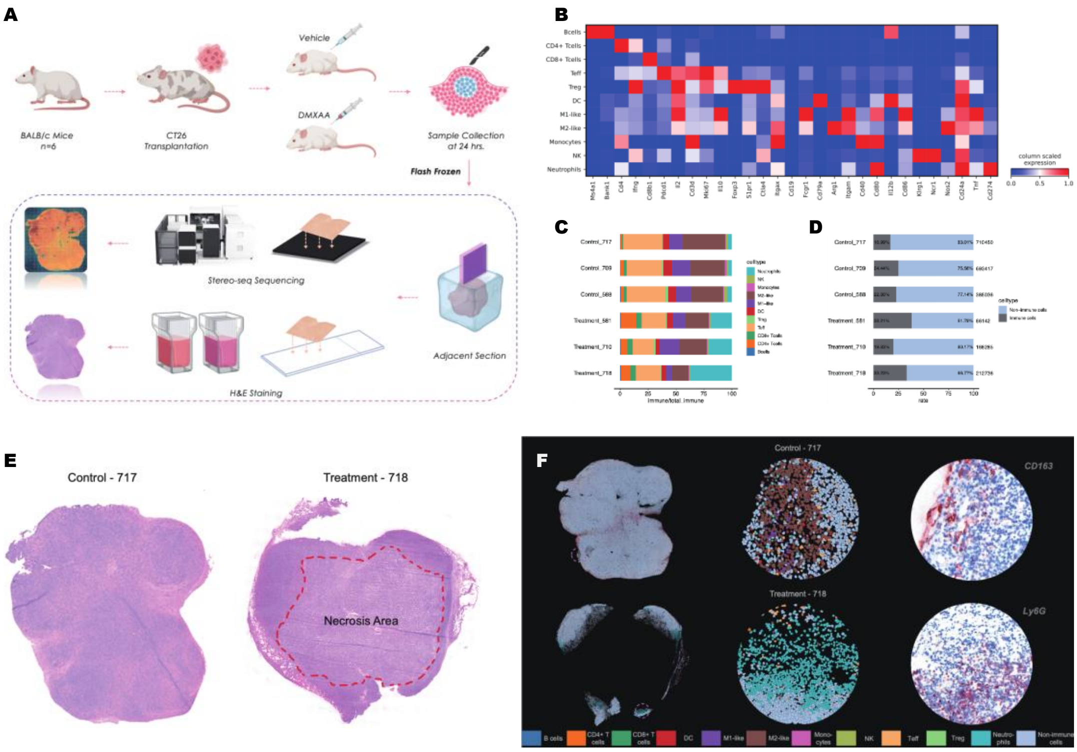

Figure 4. Spatial Transcriptomic mapping of xenograft model in situ.

A. Flow chart of xenograft model construction. BALB/c mice were subcutaneously injected with colon cancer originated CT26 cells and treated with vehicle or DMXAA for duration of 14 days after tumor transplantation and samples were harvested 24 hours after treatment. **B.** Heatmap of transcriptional markers expression of 11 annotated immune cell types at single-cell resolution. **C.** Analysis of immune cell proportion at single cell resolution in each sample. **D.** Histogram of non-immune cells vs immune cells (blue bar represents non-immune cell proportion and grey bar presents integrated immune cell proportion; numbers displayed aligned to statistical bars indicate precise cell counts regarding to each sample). **E.** Representative H&E staining of sample 717 from control group (left) and sample 718 from treatment group (right). Red-thread restricted region presents necrosis site. **F.** In situ visualization of annotated cell types using Stereo-seq data (Left); Enlarged image of marked-circle-site exhibiting cellular compositions (Middle); Representative IHC staining (CD163 indicating macrophage in

sample 717 and Ly6G indicating neutrophils in sample 718) of same marked-circle-site in middle column (Right).

Organizing TME-associated cellular neighborhood

The immune tumor microenvironment (iTME) heterogeneity prevails both intra- and inter-tumor, which is intrinsically attributed by varied cell organizations in each spatially compartmented unit. The most possible extension to interrogating and elucidating iTME of xenografts, in short of distinct histological characteristics, is to visualize tissues with CNs. With the aim of organizing iTME units that were consistently conserved across samples, we integrated the matrix co-currently covering cellular neighborhoods (CNs) and cell types (CTs) and introduced Tensor to decompose the module matrix. We first clustered windows at ranging size labelling all samples and identified distinct and exclusive CNs under different benchmarks (Fig 5A and Supplementary Fig 2A & 2B). By performing tensor decomposition in different groups (Fig 5B and Supplementary Fig 2C), we observed a given CN correlating with specific CT in each individual module (Fig 5B) and distinct euclidean distance reflecting inter-module heterogeneity (Fig 5C and Supplementary Fig 2D) in the context of bin size 100 micron, we therefore decided to interrogate with this index. As expected, constitution of CTs in each CNs significantly varied across the cohort, which in return suggested that different immune cells preferentially co-localize and associate with certain cell types in compartmented iTME units. We therefore entitled each CN based on their dominant cell proportions (Fig 5D). We next calculated the frequencies of CNs in different groups (Fig 5E) and observed a distinct correspondence of CN3 (NK cell lead), CN4 (Mixed) and CN5 (neutrophils lead) in treatment group (Fig 5F), which was aligned with the treatment background and the tensor indication. To spatially exhibit and evaluate CNs (Fig 5G), we sought to reinforce and assess CN5 in situ since neutrophils were markedly recruited by chemokine motivation but not persistent in targeted tissues[34-36]. We therefore orthotopically projected CNs to adjacent H&E staining to probe the putative distribution pattern of this neutrophils dominant iTME

unit (Fig 5H and Supplementary Fig 2E). An obvious trend of CN5 co-localizing around necrosis edges comparing to that of other CNs was observed, which potentially highlighted regional bioactivities exserted by tumor cells after immunoagonist treatment and evidenced alignment between tensor indicated transcriptomic traits and histological features, we therefore decided to further interrogate the bioactivities burst in CN5 in the following work.

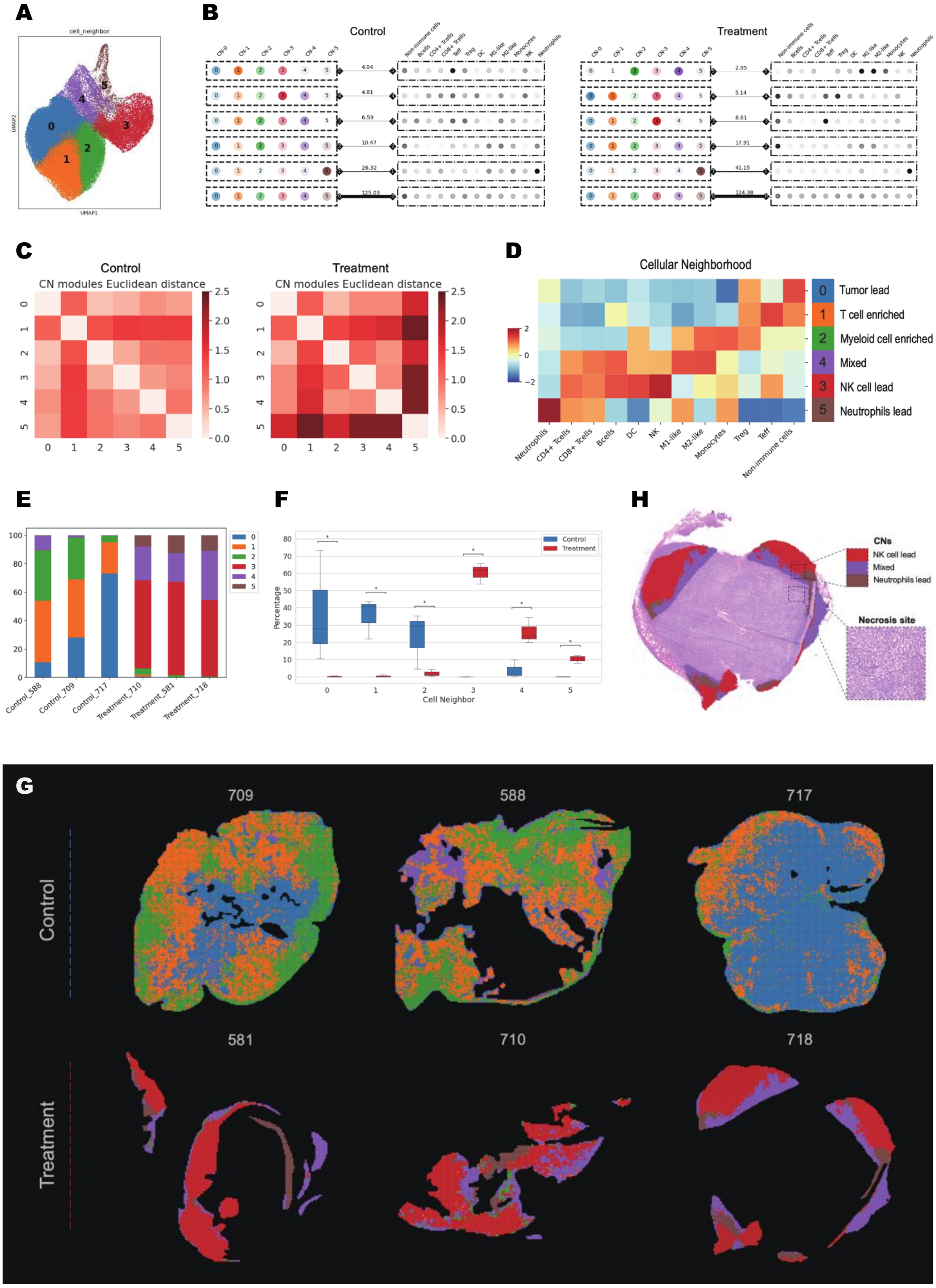

Figure 5. Construction of cellular neighborhood.

A. UMAP exhibiting the deconvolution of the identified CN clusters at bin size of 100.
B. Tucker tensor decomposition of control (left) and treatment (right) samples to stratify CN modules and CT modules. The crosstalk extent of associated CN and CT was

represented by weight of the line with indicated numbers. **C.** Heatmap of Euclidean distance between CN modules constructed in control (left) and treatment (right) group, respectively. **D.** Heatmap indicating varied cell composition in different CNs (left) and confetti labeling the corresponding title for each CN (right). **E.** CN frequencies in different samples from control and treatment group. **F.** Box plot indicating the statistical variation of CN frequency across groups. **G.** In situ distribution of CNs in different groups. **H.** Projection of CN5 on adjacent H&E staining of sample 718, with enlarged image of marked-circle-site displaying highly resolved H&E staining of necrosis area.

Deconvolving iTME regarding to individual CNs

To comprehensively depict the distinct landscape STING agonist induced, differentially expressed genes (DEG) analysis was first performed. Each CN was compared to the rest counterparts in treatment group and a potent recruitment of neutrophils and activation of STING signaling was exclusively observed in CN5[36, 37] (Fig 6A & 6B and Supplementary Fig 3A). In addition, other CNs in treatment group displayed different phenotypes (Supplementary Fig 3B), highlighting the drug-response imprinted function in CN5. Since neutrophils were leading in this milieu, we next decided to decipher the signature activities of CN5 at cellular level, SCII was applied to infer the cell-cell communications at single cell resolution. The result of CN5 indicated a potent interaction between non-immune cells and neutrophils cells with representative pair like Cxcl1/Cxcr2 and synchronously revealed prominent neutrophils-neutrophils communication with overload L-R pairs expression like Il1b/Il1r1 and Cxcl2/Cxcr2, (Fig 6C and Supplementary table 1). Meanwhile, the in situ intensity visualization of Cxcl1/Cxcr2 projecting on adjacent H&E image revealed a frequent crosstalk between non-immune cells and neutrophils around necrosis niches (Fig 6D and Supplementary Fig 3B). To deeply investigate the L-R pairs associated to treatment response, we explored the functional signaling pathways in CN5. By performing in silico identification of active transcription factors and potential interactions (Fig 6E), we identified an Nf-kappa b-centric and an Irf1-centric network

coordinating the upregulation of signature downstream targets respectively like Il1b, and Ifnβ (Fig 6F). Interestingly, Irf1 was herein determined responsible for Ifnβ regulation while Irf3 was hardly activated at this stage of treatment (Supplementary Fig 3D). Protein-protein interaction (PPI) analysis was next performed to construct the signaling network and to investigate the hub genes responsible for the signature activities in CN5. Excitingly, Il1b was the top scored gene frequently interacting with other proteins, including Ccl4, Cxcl2, Cxcl1 and Il6 (Fig 6G), which was the acquiescent downstream target of NF-kappa B in the context STING signaling activation[37]. The analysis of TF regulon and PPI network coordinately suggested that neutrophils might play key roles of regulating immunoactivities in response to STING agonist via signature signaling pathways like Nf-kappa-b and Irf1. Altogether, we provided an integrated analysis flow to depict the functional iTME unit at both molecular and cellular level.

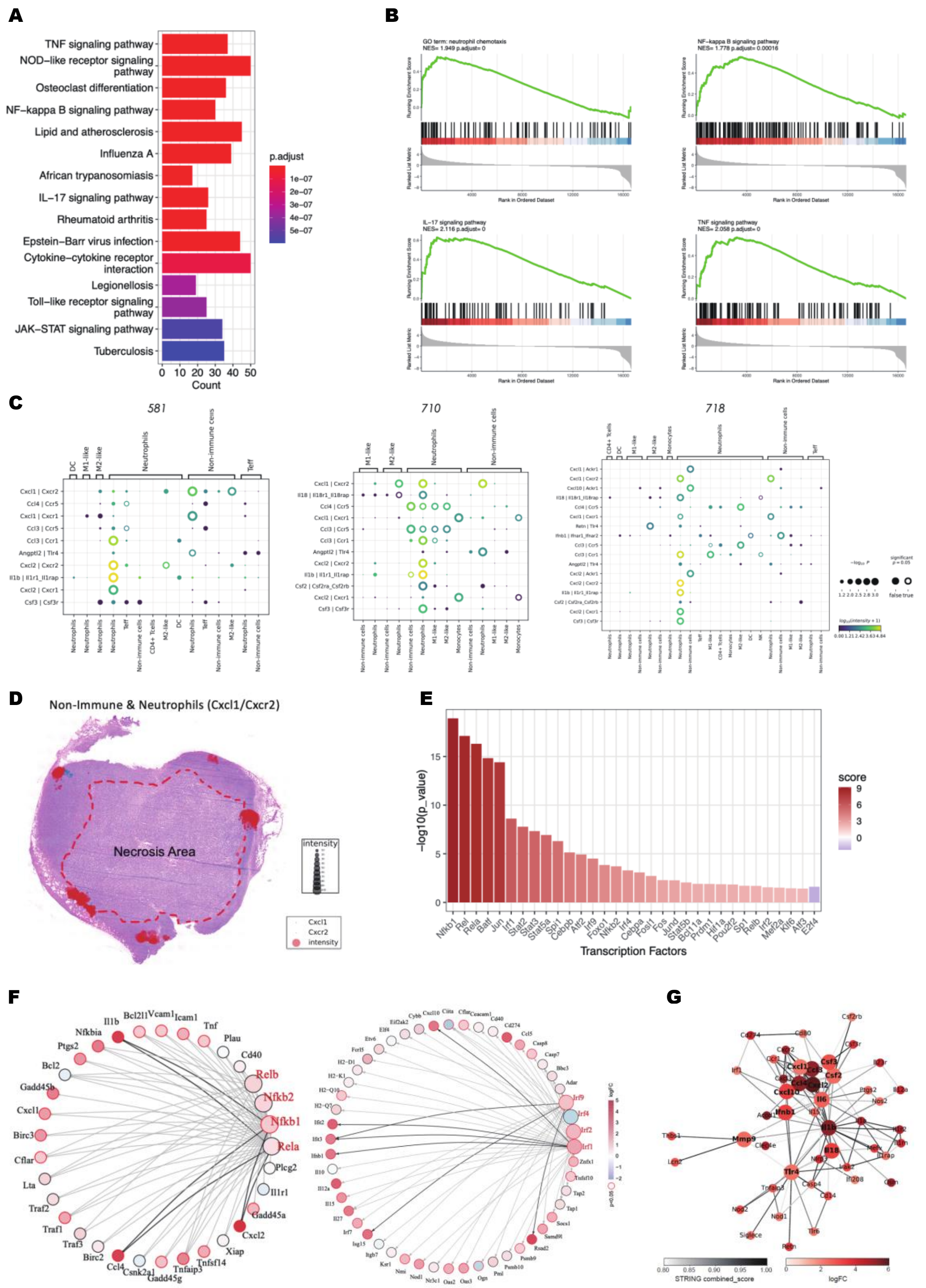

Figure 6. Deconvolution of CN of interest.

A. KEGG analysis of upregulated signaling pathways in CN5 comparing to that in counterpart CNs. **B.** GSEA analysis of indicated signaling in CN5 comparing to that in counterpart CNs. **C.**SCII calculation of representative ligand-receptor interaction in

CN5 of each sample from treatment group. **D.** In situ visualization of Cxcl1/Cxcr2 between non-immune cells and neutrophils with projection to adjacent H&E image to exhibit the crosstalk coordinates. **E.** TF regulon analysis of CN5, score represents the potency of transcriptional factor activity. **F.** Graph indicating predicted interactions between genes differentially (red, transcription factors; black, other genes) expressed in CN 5, (left) exhibited Nfkb1-centric network and (right) exhibited Irf-centric network. **G.** PPI network analysis in CN5 of treatment group, chroma of nodes represents logFC of indicated gene expression and edges represents STRING database score (confidence level). Hub genes were presented with bold font and larger size.

Discussion

With spatial transcriptomics data at single-cell resolution available[17], the state-of-art sequencing method enables researchers to obtain the genuine landscape of TME without unbalanced loss of cells caused by dissociation. With multi-dimensional and high throughput of gene expression, it requires powerful algorithmic framework to efficiently associate specific cell types to communicative activities, biological processes and clinical manifestation to address scientific questions[38]. In this paper, we developed a framework called StereoSiTE to identify cellular neighborhood unit harboring disease associated cell types, and to functionally decipher underlying biological activities of signature CN by inferring the active cell-cell communications with supplemented analysis of regional transcriptomic network. StereoSiTE is applicable to spot-based spatial datasets and compatible with high resolution resolved data like single cell resolution.

Spatially organized iTME and its disorganized manner can be manifested as pathological disease. In StereoSiTE, CN analysis helped to target the critical iTME units with involved cell types while SCII demonstrated the potential communication activities happened in these functional units with indication of associated L-R pairs. We recommend CN based analysis to identify the regions of interest with indication of neighbored cellular composition which was more likely to have active interaction due

to the proximity. CN based analysis also could accurately find the cell types with spatial signature despite low cell numbers and low transcriptomic activities. Moreover, CN based analysis offered the potent candidates for SCII to find out functional cell-cell communications, and promised the exploring of molecular mechanism with PPI and TF network, etc. Referring to scientific questions, StereoSiTE can provide clinicopathogenesis or biological event associated CNs. In previous study, spatial proteomics based CNs provided insights in CRC patients prognosis prediction with specific CNs and cell types[9], however, the limited throughput dilemma that proteomics-based technology inherent hindered the investigation of regional molecular mechanisms in each iTME units, we therefore designed this framework.

The foundation is the self-developed algorithm, SCII, which infers the active Ligand-Receptor pairs with consideration of spatial distance between interacting cells. Based on this consensus, SCII constructs the cell graph network by connecting cell pairs within a defined radius threshold. The radius threshold could be set referring to communication types. Radius threshold is recommended to be set as 100μm ~ 200μm for interactions of secreted signaling, while L-R pairs of cell-cell contact should consider close cellular proximity. Therefore, SCII incorporates the reference from CellChatDB which provides catalog associated to communicative distance. A benchmarked analysis is performed between CellPhoneDB, CellChat, Giotto, SpaTalk, and SCII with the same Stereo-seq data matrix. Figure 3 highlights that the analysis without distance threshold may lead to false positive results, and SCII showed more superior performance than other methods. What's more, enriched L-R pairs in a CN of interest within distance threshold possess high potential for association to functions.

To prove the overwhelmed performance of SCII, we applied the framework in a research scenario to explore the functional cell-cell communications in response to immuno-agonist treatment. The downstream targets and activated pathways coordinately suggested that neutrophils were markedly recruited to CN5 and STING signaling were distinctly activated in this unit. Further SCII analysis revealed a frequent crosstalk between neutrophils and neutrophils/non-immune cells and subsequent in situ

mapping implied a location preference of certain L-R pairs, like Cxcl1/Cxcr2. The TF analysis and PPI network analysis were next performed and indicated the most contributing L-R pairs, which were the downstream targets of STING activated signaling – NF-Kappa B[39]. Altogether, we depicted a neutrophil leading iTME units by defining the regional phenotype from both molecular and cellular view, which might shed light on neutrophils exserted mechanism in iTME.

Here, we presented a practical framework (StereoSiTE), which was designed to analyze the spatial transcriptomics data by incorporating open-sourced bioinformatics tools with self-developed algorithm to prove that spatial proximity is a must to guarantee an effective investigation.

Data Availability

The data that support the findings of this study have been deposited into CNGB Sequence Archive (CNSA) of China National GeneBank DataBase (CNGBdb) with accession number CNP0004910 (https://db.cngb.org/search/project/CNP0004910/), and NCBI National Center for Biotechnology Information with accession number PRJNA1087118 (https://www.ncbi.nlm.nih.gov/sra/?term=PRJNA1087118).

Code Availability

Project name: StereoSiTE

Project home page: https://github.com/STOmics/StereoSiTE

Operating system: Linux

Programming Language: Python

Other Requirements: Python3.9 or higher

License: MIT license

BiotoolsID identifiers: biotools:stereosite

RRID: SCR_025236

Method and materials

Mice and cell lines.

Female BALB/c mice at 6 weeks of age were obtained from GemPharmatech Co., Ltd. All mice were housed in a specific pathogen–free animal facility at GemPharmatech Co., Ltd. CT26 colon cancer cells were purchased from ATCC. Cells were cultured at 37 oC under 5% CO2 in DMEM supplemented with 10% FBS and 1% penicillin/streptomycin.

Xenograft tumor models and treatment.

We implanted 5×10^5 cells/100μl CT26 cells into the right flanks of BALB/c mice. When the tumors reached a volume of 250-300 mm^3, we performed intratumorally injections of the STING agonist (0.5 mg/50ul/mouse, DMXAA, Vadimezan) (43). Mice in the control group were intratumorally injected with the same volume of PBS, accordingly. Xenograft tumor samples were collected 24hours after treatment and embedded by OCT on dry ice.

Stereo-seq library preparation and sequencing

Tissue processing

Two consecutive cyro sections of 10 μm were prepared. One section was attached to glass slide and stained by H&E staining following previous protocol. The second section was adhered to the Stereo-seq chip surface and incubated at 37°C for 3-5 minutes. Then, the sections were fixed in methanol and incubated for 40 minutes at -20°C. Stereo-seq library preparation and sequencing followed previous published protocol[17].

In situ reverse transcription

Prepared section was processed according to the Stereo-seq Transcriptomics Set User Manual (STOmics) and all reagents were from the Stereo-seq Transcriptomics T kit and Stereo-seq Library Preparation kit (STOmics). Briefly, after washed with PR rinse buffer, tissue sections placed on the chip were permeabilized at 37°C for 10

minutes. RNA released from the permeabilized tissue and captured by the DNB was reverse transcribed overnight at 42°C. After reverse transcription, tissue sections were digested with Tissue Removal buffer at 55°C for 30 minutes. The resulting cDNA was then amplified.

Amplification

The collected cDNAs were amplified with KAPA HiFi Hotstart Ready Mix (Roche, KK2602) with 0.8 μM cDNA-PCR primer. PCR reactions were performed in sequential steps as incubation at 95°C for 5 minutes, 15 cycles at 98°C for 20 seconds, 58°C for 20 seconds, 72°C for 3 minutes and a final incubation at 72°C for 5 minutes.

Library construction and sequencing

The concentrations of the PCR products were quantified by Qubit™ dsDNA Assay Kit (Thermo, Q32854). A total of 20 ng of DNA were then fragmented with in-house Tn5 transposase at 55°C for 10 minutes. The reactions were stopped by the adding of 0.02% SDS and gently mixing at 37°C for 5 minutes. Fragmented products were amplified as follows: 25 μl of fragmentation product, 1 × KAPA HiFi Hotstart Ready Mix and 0.3 μM Stereo-seq-Library-F primer, 0.3 μM Stereo-seq-Library-R primer in a total volume of 100 μl with the addition of nuclease-free H2O. The reaction was then run as: 1 cycle of 95°C 5 minutes, 13 cycles of 98°C 20 seconds, 58°C 20 seconds and 72°C 30 seconds, and 1 cycle of 72°C 5 minutes. PCR products were purified using the AMPure XP Beads (0.6× and 0.15×), used for DNB generation and finally sequenced on MGI SEQ-2000 sequencer.

Data analysis

Raw sequencing data analysis

Fastq files were generated by MGI SEQ-2000 sequencer. Cell nuclei staining image stitching, tissue segmentation, gene expression register, and genome mapping, gene counts were performed by online analysis Platform: Stereo Analysis Platform

(SAP, https://uat.stomics.tech/sap/researchProject/index.html). The stitched cell nuclei staining images generate from SAP were used to generate the single cell nuclei mask through the application of a cell segmentation script sourced from the StereoCell tool[22]（ available at https://github.com/STOmics/StereoCell/tree/dev/cellbin/cell_segmentation/segment.py). The script is based on psaUnet architecture, which integrates Deep Residual net, U-Net, and EPSANet. Then gene expression matrix of each cell was generated by mapping spatial expression profile matrix and its corresponding single cell nuclei mask according to spatial coordinates. Expression profile matrix was divided into non-overlapping bins covering an area of 100 × 100 DNBs (bin100) for further cellular neighborhood construction and functional enrichment analysis. Then data structure was constructed by Scanpy[40] in python 3.9 for further analysis.

Cell type annotation

We used a single-cell transcriptomics dataset of mouse colon cancer cell line CT26[41] as a reference to deconvolute a mixture of 11 immune cell types and non-immune cells in our Stereo-seq data by Cell2location with hyperparameter N_cells_per_location= 1, detection_alpha=20. The cell type with maximum abundance was assigned to each cell, then cell types of frequency were calculated and visualized by R package ggplot2.

Cellular neighborhood construction

The tissues were binned with side-by-side windows, each with an area of 100 × 100 DNBs (bin100), representing a square with side length of 100 DNB (the unit is capturing site). As the distance between two neighbor sites was 500 nm, bin100 corresponded square with side length of 50μm. Then the cellular composition of each bin100 was deconvoluted by mapping gene expression profile of each cell type from single-cell transcriptomic dataset[33] to spatial data with cell2location. According to the deconvoluted cell composition matrix, the windows of all samples were

subsequently clustered to 7 cellular neighborhoods (CNs) by using KNN graph and Leiden with n_neighbors=19, resolution=0.32. Therefore, windows with similar cell composition were gathered to form a microenvironment. For each CN, the cell type abundance of all windows in this CN region were summed to calculate percentage and visualized by Python module Seaborn.

Benchmarking analysis for deconvolution methods

We compared the cell type deconvolution capacity of cell2location and several newer deconvolution software, including Celloscope[10], POLARIS[12], and GraphST[11], based on Kun Qu's lab provided benchmark pipeline (https://github.com/QuKunLab/SpatialBenchmarking). Following Kun Qu's benchmark pipeline, we used the mouse visual cortex STARmap dataset (https://www.starmapresources.com/data, '20180505_BY3_1kgenes'), which is a single-cell resolution spatial transcriptome, along with corresponding smart-seq data (https://portal.brain-map.org/atlases-and-data/rnaseq/mouse-v1-and-alm-smart-seq) and simulated spot-level spatial transcriptome for benchmark analysis. For the STARmap dataset, we utilized the Pearson correlation coefficient (PCC), structural similarity index (SSIM), root-mean-square error (RMSE), Jensen–Shannon divergence (JSD) score, and the aggregation of these four metrics (referred to as the Accuracy Score, abbreviated as AS score) to evaluate the performance of the four methods in predicting the cell type composition of spots or the distribution of cell-type clusters. The STARmap predicted result of cell2location from Kun Qu's group was directly used for comparison.

We also utilized a public stereo-seq dataset of liver cancer for benchmarking analysis[42]. The gene expression matrix was binned into 25 μm × 25 μm pseudo-spots (approximately one cell) as previously described. To save on computing resources, we only utilize the bottom right quarter of the expression matrix for testing. We used the kappa score to compare the software-predicted results with the reported results because there is no ground truth for the stereo-seq liver dataset.

Generally, we followed the tutorials of each software for cell-type deconvolution: Cell2location (https://github.com/BayraktarLab/cell2location), for stereo-seq liver data, the regression model of single-cell reference data was trained with parameters max_epochs = 1500, and the Cell2location model was trained with parameters max_epochs = 5000, N_cells_per_location = 3. For Celloscope (https://github.com/szczurek-lab/Celloscope), for STARmap data, the number of cells in each spot was calculated based on ground truth. For stereo-seq liver data, the number of cells in each spot was set to 1, and number_of_chains was set to 10, while other parameters were set to default. For POLARIS (https://github.com/JiawenChenn/POLARIS), for STARmap data, the layer label was set according to Kun Qu's benchmark research. For stereo-seq liver data, all spot's layer label was set to 1. For GraphST (https://github.com/JinmiaoChenLab/GraphST), all genes were set as high variable genes for STARmap data, while stereo-seq liver data set 5000 high variable genes.

Benchmarking analysis for tissue region division methods

We further used the STARmap dataset to compare the accuracy of spatial domain detection between cellular neighborhood (CN) and other local niches software (BANKSY[24], Giotto[8]) by calculating the adjusted Rand index (ARI). The STARmap dataset was simulated with 835 × 835 pixels spot-level spatial transcriptome following Kun Qu's benchmark pipeline. On the stereo-seq dataset, the default binsize of pseudo-spots is set to 100 for CN analysis, which is equivalent to 50 μm × 50 μm. The total size of the STARmap sample is 1400 μm × 300 μm. According to the spatial coordinates of STARmap, each square spot is approximately 835 × 835 pixels corresponding to bin100 of stereo-seq data. The cell-type percentage of each spot was calculated for CN analysis. We set parameters n_neighbors=15 and resolution=1.1 to obtain seven clusters (corresponding to the number of spatial domains in the dataset) for ARI calculation. As Shyam Prabhakar's group has already calculated the Adjusted Rand Index (ARI) of BANKSY and other software in the STARmap dataset[13], we

then calculated the ARI of the CN result and compared it directly with their findings.

Tensor decomposition

For each group, we constructed a tensor with 3×7×12 dimensions (3 samples, 7 CNs and 12 cell types). We performed Non-negative Tucker decomposition by Python package Tensorly[33]. By calculating the decomposition losses of different combinations of the number of CN modules and CT modules, we selected the suitable rank in the elbow point to perform non-negative tensor decomposition (Supplementary Fig 1B). The visualization of decomposition result refers to Schürch's article[9].

Functional enrichment analysis and transcription factors activity inference

We performed differential expression analysis on different CNs using the edgeR package[43] in pseudobulk manner[44]. Differential expressed genes were retained when abs(logfoldchanges)>1 and pvalue<0.05. The KEGG enrichment analysis, gene ontology enrichment analysis and GSEA were employed to dissect the biological function of CN5 using functions of R package ClusterProfiler[45]. The top 15 significantly enriched pathways of KEGG enrichment analysis and gene ontology enrichment analysis was displayed as bar plot, respectively. The interesting pathways of GSEA were shown through the gseaplot2 function. The R package decoupleR[46] and DoRothEA[47] was used to perform transcription factors (TF) activity inference with Univariate Linear Model, the most significantly TF regulatory network was exhibited by igraph package.

Spatial cell interaction intensity

Firstly, we constructed the space nearest neighbor graph based on spatial coordinate of all cells, and cell pairs with distance less than the radius threshold were connected by edges. Secondly, the edges were assigned with weight by summing up the ligand and receptor gene expression of the sender and receiver cells (ends nodes of the edge), then the edges with weight 0 (no co-expression) were removed and the edges with

weight more than 0 (co-expression) were reserved. Thirdly, we computed the locally spatial cell interaction intensity of every sender cell with its linked receiver cells by summing the edge weights between them, defined as

$$intensity = \sum_{i=0}^{n} edgeWeight_i(receiver_i) \quad \text{(n = number of surrounding receiver cells) (1)}$$

Besides, the interaction intensities from sender cell to receiver cell mediated by specific L-R pairs of the entire slide equaled the sum of weight of all edges. The formula below presents the computation rule.

$$intensities = \sum_{i=0}^{N} edgeWeight_i \quad (N = total\ edges\ of\ whole\ slide) \qquad (2)$$

As to the complex ligand or receptor which was composed of several subunits, we selected the minimal expression or calculated the mean expression of all submits to compute the SCII.

We used the LR database from CellChatDB in our research for its clear classification of communication types associated to different active distance. Also, the selection of databases is flexible, and customer defined. It allows users to introduce LR databases of interest. The database file should be formatted in CSV format and contains columns “source” and “target”, which indicate the ligand and receptor. If users want to process different types of LR with different strategies, the columns “annotation” indicating LR types is required.

To investigate the superiority of SCII compared with CCI method ignoring cell spatial distribution, we calculated the distance of inferred interactions by counting the median distance of the cell graph network constructed by applying KNN algorithm to each cell to connect it with K nearest cells.

Comparison between SCII with other Cell-Cell Interaction (CCI) methods

Most of other cell-cell interaction inference methods (CellPhoneDB, CellChat,

Giotto, Spatalk) were implemented by R and their computing performance were limited, resulting the disability of analyzing a whole stereo-seq data of one sample which contains 403516 cells. Considering the incompetent, we extracted a small part tissue region from the original data, which contains 11214 cells. Considering the LR pairs database divergence of each method and some methods can't process protein complexes, we extracted the common LR pairs of CellPhoneDB and CellChatDB, then filtered out LR pairs constructed with complexes. The remaining LR pairs are used to run cell-cell interaction inference by each method. Except some cell and gene filter parameters were turned down, because of the limited gene capture of spatial transcription technology at single cell resolution, all methods were benchmarked with default parameters. We calculated the overlap of inferred interactions from different methods by set operation and Jaccard index, and the co-expressed percent of the CCI by counting the number of spatial proximal sender and receiver cell pairs which expressed corresponding ligand and receptor genes from the cell nearest neighbor graph.

Protein-protein interaction analysis

We queried 628 significant up regulated genes (logFC>2 & FDR<0.05) in CN5 area of treatment samples in STRING v11.5 database[48] (score cutoff = 0.4) and 505 proteins be found, after filtered 95 nodes with 0 degree, we get a protein-protein interaction (ppi) network with 402 proteins and 2042 edges. Then we performed markov clustering by MCL cluster algorithm[49] with parameter (I=3.0) to get functional ppi network. The largest cluster with 82 proteins and 821 edges was further used to found hub genes by ranking degree and Maximal Clique Centrality (MCC) score in python module NetworkX 3.1[50]. The highest MCC-scored genes within top 10 degree were labeled as hub genes and their interactions with STRING confidence score over 0.8 were considered as key ppi networks.

STAR

Stereo Analysis	https://uat.stomics.tech/sap/researchProject/index.html

Platform (SAP)	
Python 3.9	https://www.python.org/
Numpy 1.22.4	https://numpy.org/
Pandas 1.5.1	https://pandas.pydata.org/
Sklearn 1.0.1	https://scikit-learn.org/
Cell2location 0.1	https://cell2location.readthedocs.io/en/latest/
Scanpy 1.9.1	https://scanpy.readthedocs.io/en/stable/index.html
Tensorly 0.7.0	http://tensorly.org/stable/index.html
Seaborn 0.11.2	https://seaborn.pydata.org/index.html
Squidpy 1.1.2	https://squidpy.readthedocs.io/en/stable/index.html
NetworkX 3.1	https://networkx.org/
STRING v11.5	https://string-db.org/
mcl 22-282	https://github.com/micans/mcl
R 4.2.1	https://www.r-project.org/
ggplot2 3.4.0	https://ggplot2.tidyverse.org/
ClusterProfiler 4.6.0	https://bioconductor.org/packages/release/bioc/html/clusterProfiler.html
edgeR 3.40.2	https://bioconductor.org/packages/release/bioc/html/edgeR.html
Dorothea 1.10.0	https://bioconductor.org/packages/release/data/experiment/html/dorothea.html
decoupleR 2.4.0	https://www.bioconductor.org/packages/release/bioc/html/decoupleR.html
igraph 1.4.3	https://r.igraph.org/
CellPhoneDB v4	https://cellphonedb.readthedocs.io/en/latest/index.html
CellChat v2	https://github.com/jinworks/CellChat
Giotto	https://drieslab.github.io/Giotto_website/
SpaTalk	https://github.com/ZJUFanLab/SpaTalk

Celloscope	https://github.com/szczurek-lab/Celloscope
POLARIS	https://github.com/JiawenChenn/POLARIS
GraphST	https://deepst-tutorials.readthedocs.io/en/latest/
BANKSY	https://github.com/prabhakarlab/Banksy

Conflict of Interest

The authors report no conflicts of interest in this work.

Acknowledgments

The authors would like to acknowledge China National GeneBank. The authors would like to acknowledge Ms. Meisong Yang (BGI Research-Shenzhen) for her assistance in Stereo-seq experiments.

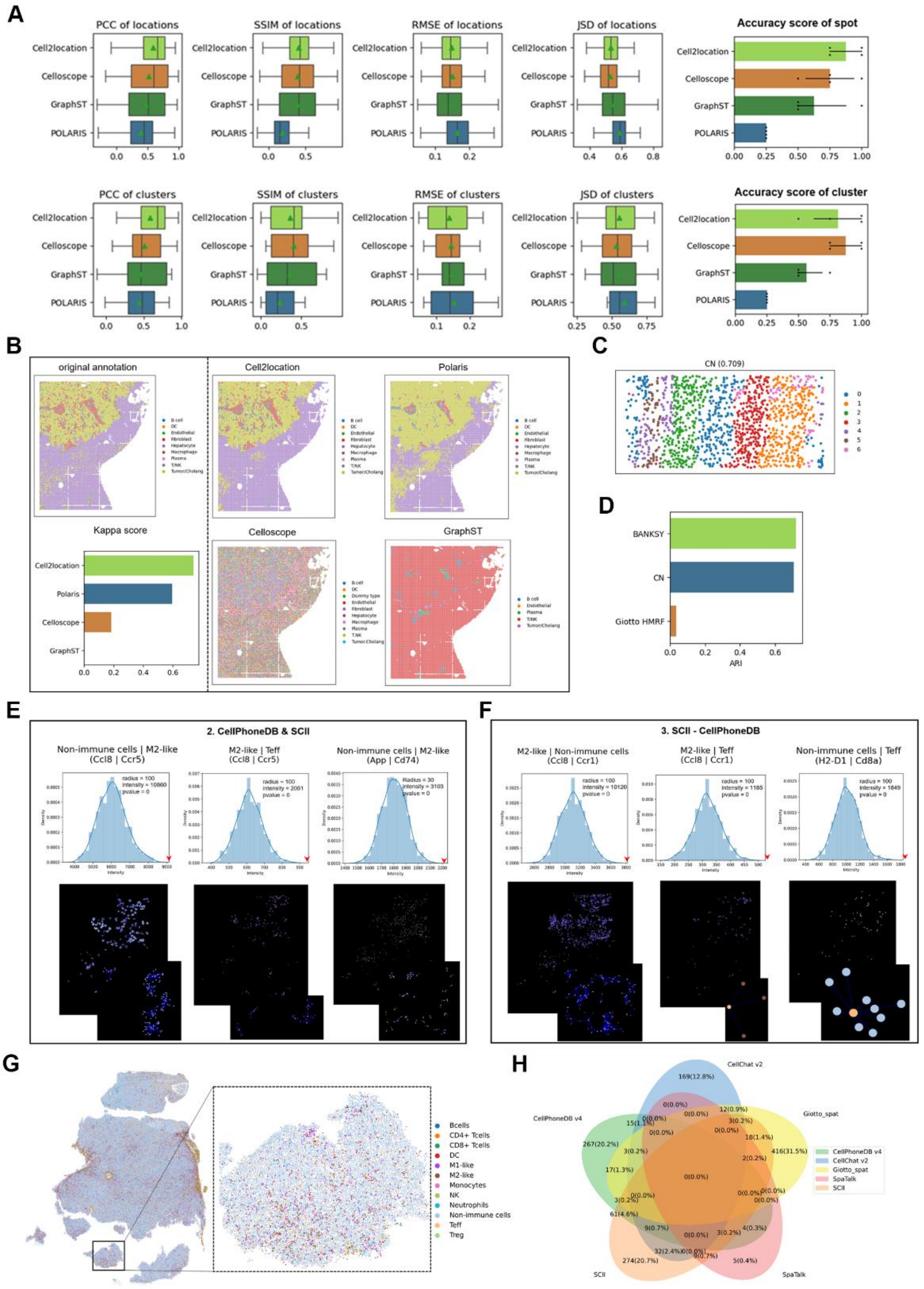

Supplementary Figure 1. Performance comparison among different cell type deconvolution methods, tissue domain division methods, and intercellular communication inference methods.

A. PCC, SSIM, RMSE, and JSD values for the cell type composition of the spots (top) or clusters (bottom) in the STARmap dataset, as measured by four deconvolution methods. The accuracy score of the spots or clusters aggregated from the corresponding PCC, SSIM, RMSE, and JSD is shown on the right. PCC: Pearson correlation coefficient, SSIM: structural similarity index, RMSE: root mean square error, JSD: Jensen-Shannon divergence. **B.** Spatial distribution of cell types predicted by each deconvolution method for the stereo-seq dataset of liver cancer. Each result was compared with the original cell type annotation obtained from the original paper, and the interrater reliability was indicated by the kappa statistic (bottom left). **C.** Spatial distribution of tissue domain divided by cellular neighborhood (CN) for the STARmap dataset. **D.** Bar plot displayed the Adjusted Rand Index (ARI) of domain division results generated by BANSKY, CN, and Giotto HMRF for the STARmap dataset. **E.** Extracted a portion of the stereo-seq demo data for comparing SCII with other cell-cell interaction (CCI) inference methods. The rectangular box indicates the selected region. **F.** Venn diagram illustrated the intersection and differences in inferred interactions from various CCI inference methods (CellphoneDB v4, CellChat v2, Spatalk, Giotto). **G, H.** Null distribution generated by permutation test for each interaction (top) produced by both CellPhoneDB and SCII, and by SCII alone. The red arrow indicates the actual intensity measured by SCII. The spatial distribution of connections between sender cells and receiver cells was shown at the bottom, with a zoomed-in region displayed for clearer observation.

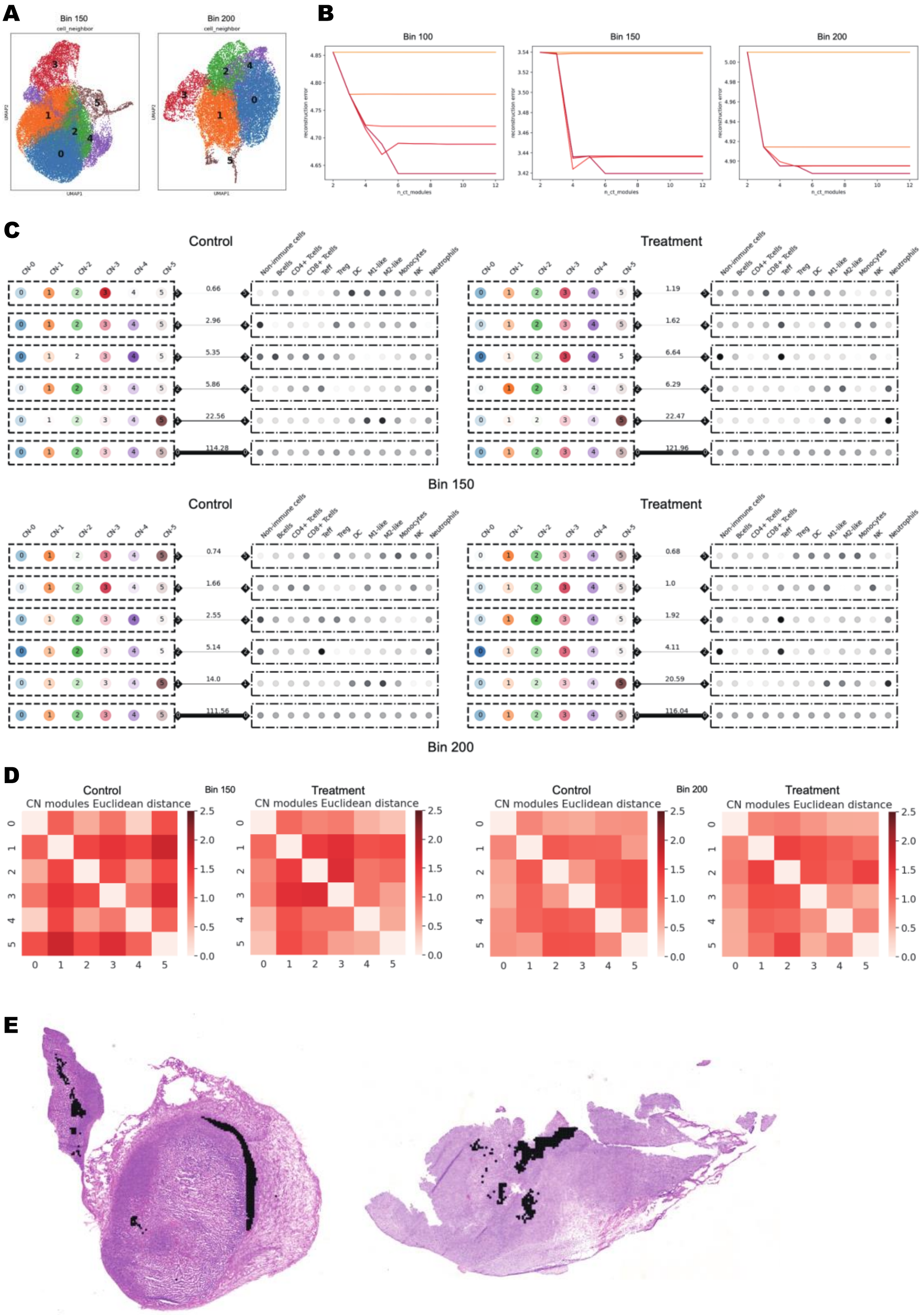

Supplementary Figure 2. Construction of cellular neighborhood.

A. UMAP exhibiting the deconvolution of the identified CN clusters at bin size of 150 (left) and 200 (right); **B.** Rank selection of Tucker tensor decomposition at different bin size to stratify CN modules and CT modules. Tensor decomposition loss in different

CN modules (different colors) or CT modules numbers (x axis). **C.** Decomposition results for both groups at bin size 150 and 200. The crosstalk extent of associated CN and CT was represented by weight of the line with indicated numbers. **D.** Heatmap of Euclidean distance between CN modules constructed in control (left) and treatment (right) group at indicated bin size, respectively. E. Projection of CN5 on adjacent H&E staining of sample 518 (left) and 710 (right).

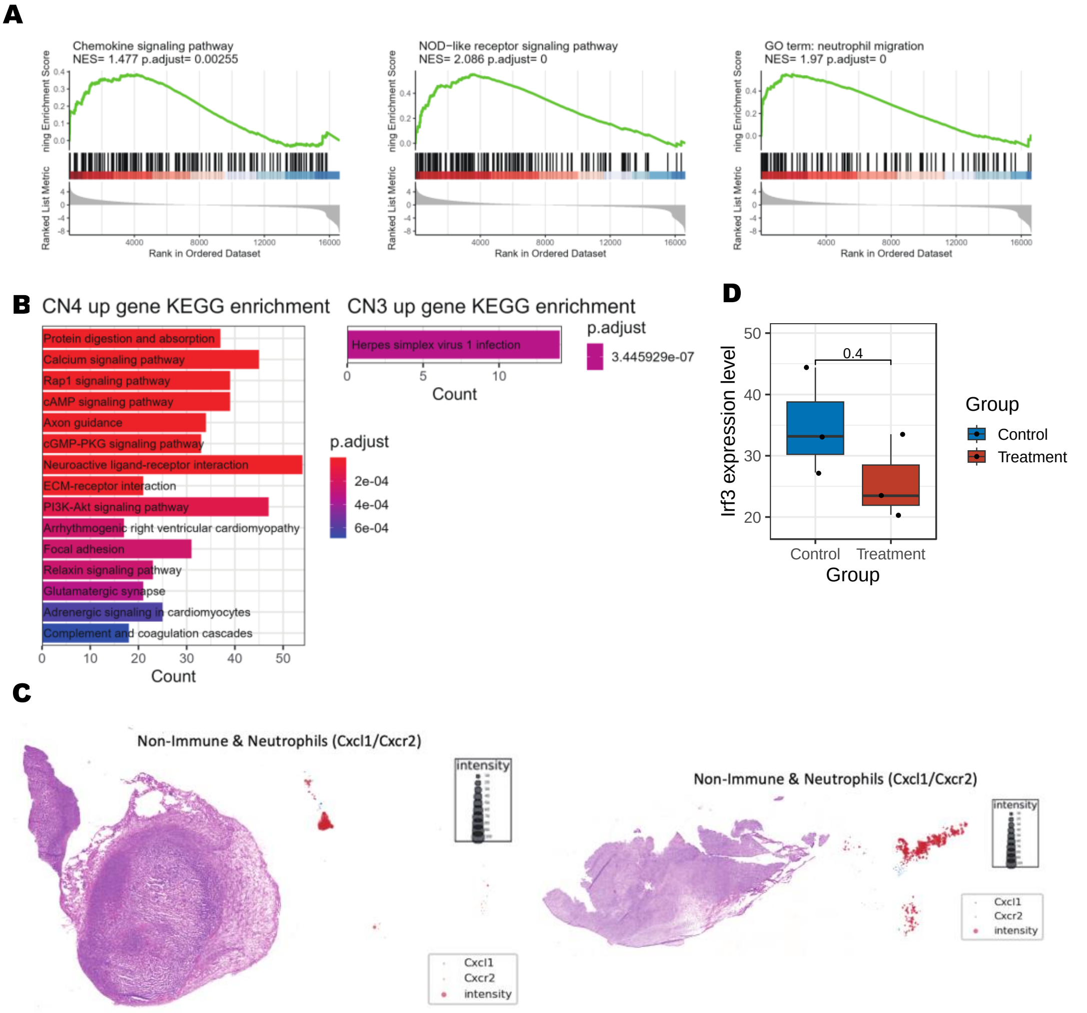

Supplementary Figure 3. Deconvolution of CN of interest.

A. GSEA analysis of indicated signaling in CN5 comparing to that in counterpart CNs. **B.** KEGG analysis of upregulated signaling pathways respectively in CN4 (left) and CN3 (right) comparing to that in counterpart CNs. **C.** In situ visualization of

Cxcl1/Cxcr2 between non-immune cells and neutrophils in 518 (left) and 710 (right) with adjacent H&E image displayed to exhibit the crosstalk coordinates. D. Box plot of Irf3 expression in control groups and treatment groups (P = 0.4).

Reference

1. Baghba R, Roshangar L, Jahanban-Esfahlan R, Seidi K, Ebrahimi-Kalan A, Jaymand M, et al. Tumor microenvironment complexity and therapeutic implications at a glance. Cell Commun Signal. 2020;18 1 doi:10.1186/s12964-020-0530-4.
2. Wu YC, Cheng YF, Wang XD, Fan J and Gao Q. Spatial omics: Navigating to the golden era of cancer research. Clin Transl Med. 2022;12 1 doi:10.1002/ctm2.696.
3. Zhang L, Chen D, Song D, Liu X, Zhang Y, Xu X, et al. Clinical and translational values of spatial transcriptomics. Signal Transduct Target Ther. 2022;7 1:111. doi:10.1038/s41392-022-00960-w.
4. Efremova M, Vento-Tormo M, Teichmann SA and Vento-Tormo R. CellPhoneDB: inferring cell-cell communication from combined expression of multi-subunit ligand-receptor complexes. Nat Protoc. 2020;15 4:1484-506. doi:10.1038/s41596-020-0292-x.
5. Jin SQ, Guerrero-Juarez CF, Zhang LH, Chang I, Ramos R, Kuan CH, et al. Inference and analysis of cell-cell communication using CellChat. Nat Commun. 2021;12 1 doi:10.1038/s41467-021-21246-9.
6. Shao X, Li CY, Yang HH, Lu XY, Liao J, Qian JY, et al. Knowledge-graph-based cell-cell communication inference for spatially resolved transcriptomic data with SpaTalk. Nat Commun. 2022;13 1 doi:10.1038/s41467-022-32111-8.
7. Pham D, Tan X, Xu J, Grice LF, Lam PY, Raghubar A, et al. stLearn: integrating spatial location, tissue morphology and gene expression to find cell types, cell-cell interactions and spatial trajectories within undissociated tissues. bioRxiv. 2020:2020.05.31.125658. doi:10.1101/2020.05.31.125658.
8. Dries R, Zhu Q, Dong R, Eng CHL, Li HP, Liu K, et al. Giotto: a toolbox for integrative analysis and visualization of spatial expression data. Genome Biol. 2021;22 1 doi:10.1186/s13059-021-02286-2.
9. Schürch CM, Bhate SS, Barlow GL, Phillips DJ, Noti L, Zlobec I, et al. Coordinated Cellular Neighborhoods Orchestrate Antitumoral Immunity at the Colorectal Cancer Invasive Front (vol 182, pg 1341, 2020). Cell. 2020;183 3:838-. doi:10.1016/j.cell.2020.10.021.
10. Geras A, Shafighi SD, Domzal K, Filipiuk I, Raczkowski L, Szymczak P, et al. Celloscope: a probabilistic model for marker-gene-driven cell type deconvolution in spatial transcriptomics data. Genome Biol. 2023;24 1 doi:10.1186/s13059-023-02951-8.
11. Long YH, Ang KS, Li MW, Chong KLK, Sethi R, Zhong CW, et al. Spatially informed clustering, integration, and deconvolution of spatial transcriptomics with GraphST. Nat Commun. 2023;14 1 doi:10.1038/s41467-023-36796-3.
12. Chen JW, Luo TY, Jiang MZ, Liu JD, Gupta GP and Li Y. Cell composition

inference and identification of layer-specific spatial transcriptional profiles with POLARIS. Sci Adv. 2023;9 9 doi:10.1126/sciadv.add9818.

13. Li B, Zhang W, Guo C, Xu H, Li LF, Fang MH, et al. Benchmarking spatial and single-cell transcriptomics integration methods for transcript distribution prediction and cell type deconvolution. Nat Methods. 2022;19 6:662-+. doi:10.1038/s41592-022-01480-9.

14. Kleshchevnikov V, Shmatko A, Dann E, Aivazidis A, King HW, Li T, et al. Cell2location maps fine-grained cell types in spatial transcriptomics. Nat Biotechnol. 2022;40 5:661-+. doi:10.1038/s41587-021-01139-4.

15. Traag VA, Waltman L and van Eck NJ. From Louvain to Leiden: guaranteeing well-connected communities. Sci Rep-Uk. 2019;9 doi:10.1038/s41598-019-41695-z.

16. Hore V, Viñuela A, Buil A, Knight J, McCarthy MI, Small K, et al. Tensor decomposition for multiple-tissue gene expression experiments. Nat Genet. 2016;48 9:1094-+. doi:10.1038/ng.3624.

17. Chen A, Liao S, Cheng M, Ma K, Wu L, Lai Y, et al. Spatiotemporal transcriptomic atlas of mouse organogenesis using DNA nanoball-patterned arrays. Cell. 2022;185 10:1777-92.e21. doi:10.1016/j.cell.2022.04.003.

18. Cho CS, Xi JY, Si YC, Park SR, Hsu JE, Kim M, et al. Microscopic examination of spatial transcriptome using Seq-Scope. Cell. 2021;184 13:3559-+. doi:10.1016/j.cell.2021.05.010.

19. Chen KH, Boettiger AN, Moffitt JR, Wang SY and Zhuang XW. Spatially resolved, highly multiplexed RNA profiling in single cells. Science. 2015;348 6233 doi:10.1126/science.aaa6090.

20. Eng CHL, Lawson M, Zhu Q, Dries R, Koulena N, Takei Y, et al. Transcriptome-scale super-resolved imaging in tissues by RNA seqFISH. Nature. 2019;568 7751:235-+. doi:10.1038/s41586-019-1049-y.

21. Wang X, Allen WE, Wright MA, Sylwestrak EL, Samusik N, Vesuna S, et al. Three-dimensional intact-tissue sequencing of single-cell transcriptional states. Science. 2018;361 6400 doi:ARTN eaat5691
10.1126/science.aat5691.

22. Li M, Liu H, Li M, Fang S, Kang Q, Zhang J, et al. StereoCell enables high accuracy single cell segmentation for spatial transcriptomic dataset. bioRxiv. 2023:2023.02.28.530414. doi:10.1101/2023.02.28.530414.

23. Palla G, Spitzer H, Klein M, Fischer D, Schaar AC, Kuemmerle LB, et al. Squidpy: a scalable framework for spatial omics analysis. Nat Methods. 2022;19 2:171-+. doi:10.1038/s41592-021-01358-2.

24. Singhal V, Chou NG, Lee JS, Yue YF, Liu JY, Chock WK, et al. BANKSY unifies cell typing and tissue domain segmentation for scalable spatial omics data analysis. Nat Genet. 2024; doi:10.1038/s41588-024-01664-3.

25. Hu J, Li XJ, Coleman K, Schroeder A, Ma N, Irwin DJ, et al. SpaGCN: Integrating gene expression, spatial location and histology to identify spatial domains and spatially variable genes by graph convolutional network. Nat Methods. 2021;18 11:1342-+. doi:10.1038/s41592-021-01255-8.

26. Chidester B, Zhou TM, Alam S and Ma J. SpiceMix enables integrative single-cell spatial modeling of cell identity. Nat Genet. 2023;55 1:78-+. doi:10.1038/s41588-022-01256-z.
27. Dong KN and Zhang SH. Deciphering spatial domains from spatially resolved transcriptomics with an adaptive graph attention auto-encoder. Nat Commun. 2022;13 1 doi:10.1038/s41467-022-29439-6.
28. Zhao E, Stone MR, Ren X, Guenthoer J, Smythe KS, Pulliam T, et al. Spatial transcriptomics at subspot resolution with BayesSpace. Nat Biotechnol. 2021;39 11:1375-+. doi:10.1038/s41587-021-00935-2.
29. Li L, Su H, Ji Y, Zhu F, Deng J, Bai X, et al. Deciphering Cell–Cell Interactions with Integrative Single-Cell Secretion Profiling. Advanced Science. 2023;10 19:2301018. doi:https://doi.org/10.1002/advs.202301018.
30. Matsuda S, Matsuda Y and D'Adamio L. CD74 interacts with APP and suppresses the production of Aβ. Mol Neurodegener. 2009;4 doi:10.1186/1750-1326-4-41.
31. Jin S, Plikus MV and Nie Q. CellChat for systematic analysis of cell-cell communication from single-cell and spatially resolved transcriptomics. bioRxiv. 2023:2023.11.05.565674. doi:10.1101/2023.11.05.565674.
32. Conlon J, Burdette DL, Sharma S, Bhat N, Thompson M, Jiang Z, et al. Mouse, but not human STING, binds and signals in response to the vascular disrupting agent 5,6-dimethylxanthenone-4-acetic acid. J Immunol. 2013;190 10:5216-25. doi:10.4049/jimmunol.1300097.
33. Ravirala D, Pei G, Zhao Z and Zhang X. Comprehensive characterization of tumor immune landscape following oncolytic virotherapy by single-cell RNA sequencing. Cancer Immunol Immunother. 2022;71 6:1479-95. doi:10.1007/s00262-021-03084-2.
34. Coffelt SB, Wellenstein MD and de Visser KE. Neutrophils in cancer: neutral no more. Nat Rev Cancer. 2016;16 7:431-46. doi:10.1038/nrc.2016.52.
35. Jaillon S, Ponzetta A, Di Mitri D, Santoni A, Bonecchi R and Mantovani A. Neutrophil diversity and plasticity in tumour progression and therapy. Nat Rev Cancer. 2020;20 9:485-503. doi:10.1038/s41568-020-0281-y.
36. Shaul ME and Fridlender ZG. Tumour-associated neutrophils in patients with cancer. Nat Rev Clin Oncol. 2019;16 10:601-20. doi:10.1038/s41571-019-0222-4.
37. Motwani M, Pesiridis S and Fitzgerald KA. DNA sensing by the cGAS-STING pathway in health and disease. Nat Rev Genet. 2019;20 11:657-74. doi:10.1038/s41576-019-0151-1.
38. Longo SK, Guo MG, Ji AL and Khavari PA. Integrating single-cell and spatial transcriptomics to elucidate intercellular tissue dynamics. Nat Rev Genet. 2021;22 10:627-44. doi:10.1038/s41576-021-00370-8.
39. Motwani M, Pesiridis S and Fitzgerald KA. DNA sensing by the cGAS-STING pathway in health and disease. Nat Rev Genet. 2019;20 11:657-74. doi:10.1038/s41576-019-0151-1.
40. Wolf FA, Angerer P and Theis FJ. SCANPY: large-scale single-cell gene expression data analysis. Genome Biol. 2018;19 1:15. doi:10.1186/s13059-017-

1382-0.

41. Hunter MV, Moncada R, Weiss JM, Yanai I and White RM. Spatially resolved transcriptomics reveals the architecture of the tumor-microenvironment interface. Nat Commun. 2021;12 1:6278. doi:10.1038/s41467-021-26614-z.

42. Wu L, Yan JY, Bai YQ, Chen FY, Zou XX, Xu JS, et al. An invasive zone in human liver cancer identified by Stereo-seq promotes hepatocyte-tumor cell crosstalk, local immunosuppression and tumor progression. Cell Res. 2023;33 8:585-603. doi:10.1038/s41422-023-00831-1.

43. Robinson MD, McCarthy DJ and Smyth GK. edgeR: a Bioconductor package for differential expression analysis of digital gene expression data. Bioinformatics. 2010;26 1:139-40. doi:10.1093/bioinformatics/btp616.

44. Squair JW, Gautier M, Kathe C, Anderson MA, James ND, Hutson TH, et al. Confronting false discoveries in single-cell differential expression. Nat Commun. 2021;12 1:5692. doi:10.1038/s41467-021-25960-2.

45. Yu G, Wang LG, Han Y and He QY. clusterProfiler: an R package for comparing biological themes among gene clusters. Omics. 2012;16 5:284-7. doi:10.1089/omi.2011.0118.

46. Badia IMP, Vélez Santiago J, Braunger J, Geiss C, Dimitrov D, Müller-Dott S, et al. decoupleR: ensemble of computational methods to infer biological activities from omics data. Bioinform Adv. 2022;2 1:vbac016. doi:10.1093/bioadv/vbac016.

47. Garcia-Alonso L, Holland CH, Ibrahim MM, Turei D and Saez-Rodriguez J. Benchmark and integration of resources for the estimation of human transcription factor activities. Genome Res. 2019;29 8:1363-75. doi:10.1101/gr.240663.118.

48. Szklarczyk D, Gable AL, Lyon D, Junge A, Wyder S, Huerta-Cepas J, et al. STRING v11: protein-protein association networks with increased coverage, supporting functional discovery in genome-wide experimental datasets. Nucleic Acids Res. 2019;47 D1:D607-d13. doi:10.1093/nar/gky1131.

49. Enright AJ, Van Dongen S and Ouzounis CA. An efficient algorithm for large-scale detection of protein families. Nucleic Acids Res. 2002;30 7:1575-84. doi:DOI 10.1093/nar/30.7.1575.

50. Hagberg AaS, Pieter J. and Schult, Daniel A. Exploring network structure, dynamics, and function using NetworkX. 2008.

Click here to access/download
Supplementary Material
Supplementary Table 2.csv

Click here to access/download
Supplementary Material
Response to Reviewers.docx
