# Supplementary material for: StereoSiTE: a framework to spatially and quantitatively profile the cellular neighborhood organized iTME
Source: Gigascience. 2024 Oct 25;13:giae078. doi: 10.1093/gigascience/giae078 (PMC11503478; doi:10.1093/gigascience/giae078)
Supplement: giae078_Supplemental_Figures_and_Tables [file giae078_supplemental_figures_and_tables.zip › Supplemental_Figures_and_Tables/Supplementary Figures.pdf]

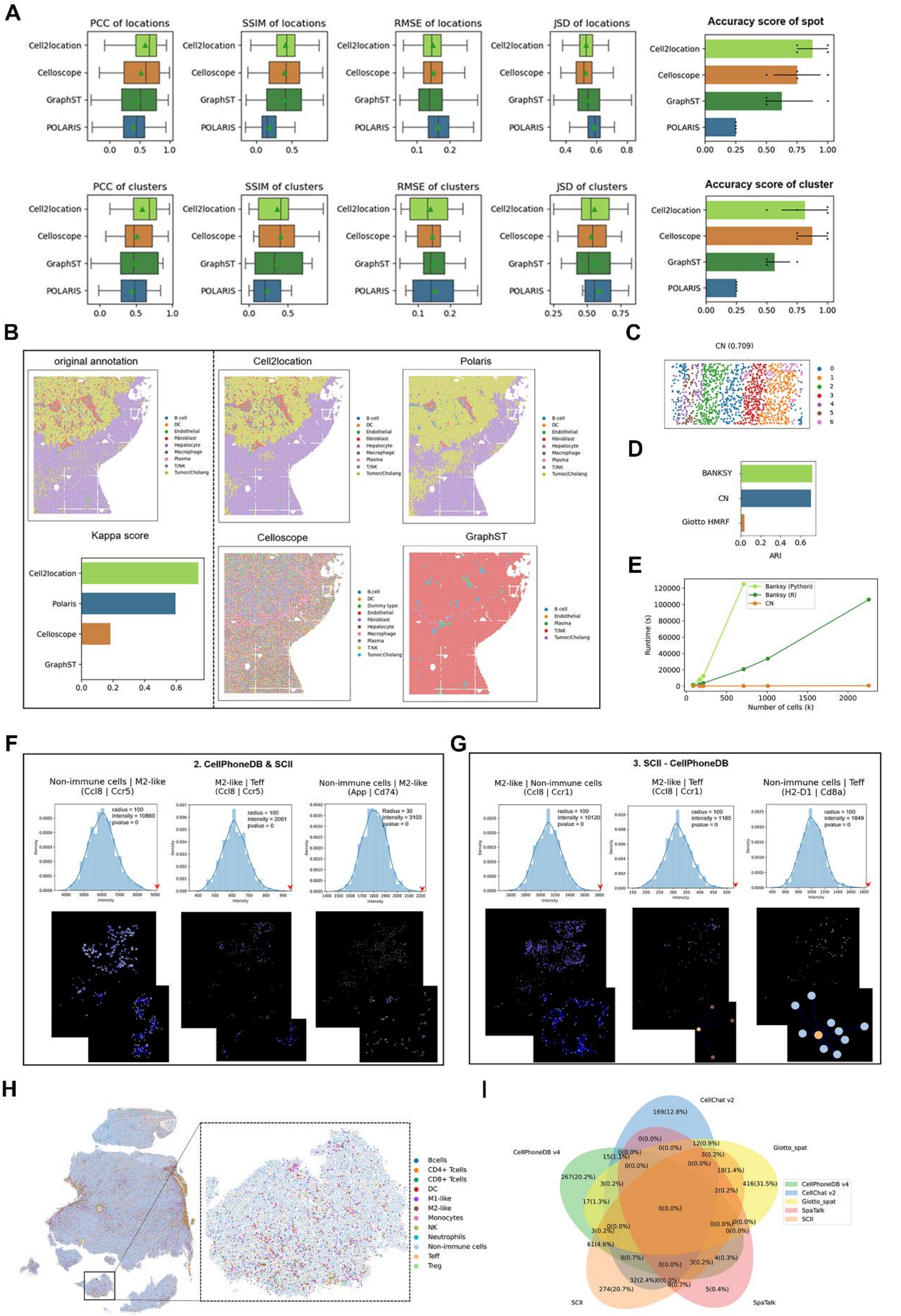

Supplementary Fig. S1. Performance comparison among different cell-type deconvolution methods, tissue domain division methods, and intercellular communication inference methods. (A) The performance correlation coefficient (PCC), structural similarity index (SSIM), root mean square error (RMSE), and Jensen–

Shannon divergence (JSD) values were calculated to assess the ac□curacy of cell-type composition in the STARmap dataset. These metrics were evaluated for both individual spots (top) and clusters (bottom) using 4 deconvolution methods. The aggregated ac□curacy scores for spots or clusters were derived from the PCC, SSIM, RMSE, and JSD values. (B) The spatial distribution of cell types predicted by each deconvolution method was examined in the stereo-seq dataset of liver cancer. A comparison was made between the results and the original cell-type annotations from the primary literature. The agreement between the different annotations was quantified using the kappa statistic (bottom left). (C) The spatial distribution of tissue domains segmented by cellular neighborhood (CN) was analyzed in the STARmap dataset. (D) Bar plot displaying the adjusted Rand index (ARI) for the domain di□vision results obtained from BANSKY, CN, and Giotto HMRF in the STARmap dataset. (E) Runtimes of CN and BANSKY for increasing cell numbers, up to 2 million cells. (F, G) Null distributions were generated through permutation tests for each interaction (top) produced by both CellPhoneDB and SCII, as well as by SCII alone. The red arrow indicates the actual intensity measured by SCII. The spatial distribution of connections between sender cells and receiver cells is shown at the bottom, with a zoomed-in region dis□played for clearer observation. (H) A subset of the stereo-seq demo data was extracted to compare the SCII method with other cell–cell interaction (CCI) inference techniques. The selected region is highlighted within a rectangular box. (I) Venn diagram was used to visualize the overlap and distinctions in the inferred interactions from various CCI inference methods, including CellphoneDB v4, CellChat v2, Spatalk, and Giotto.

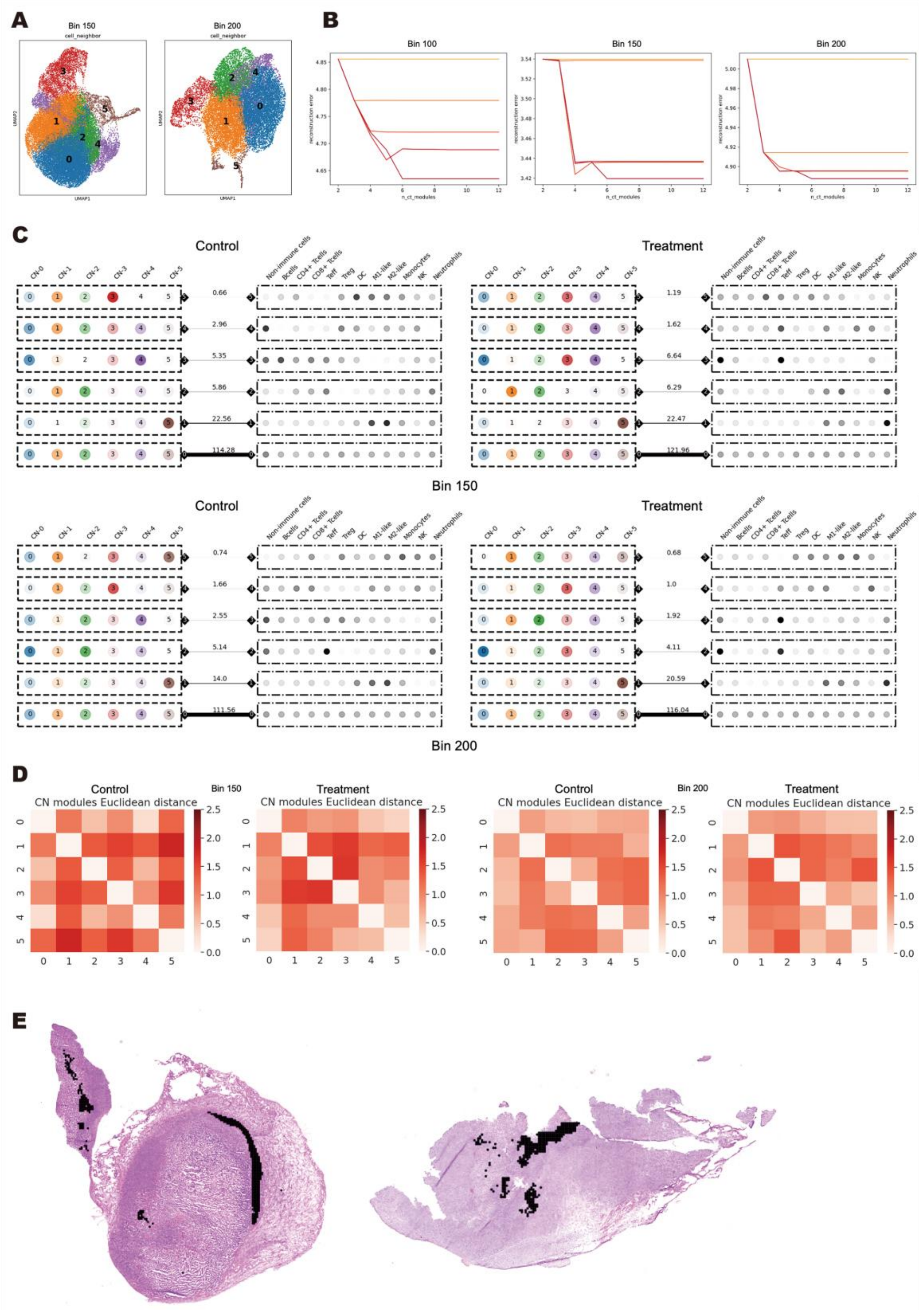

Supplementary Fig. S2. Construction of cellular neighborhood. (A) UMAP exhibiting the deconvolution of the identified CN clusters at a bin size of 150 (left) and 200 (right). (B) Rank selection of Tucker tensor decomposition at different bin sizes to stratify CN modules and CT modules. The tensor decomposition loss is shown in different CN modules (in different colors) or CT modules numbers (x-axis). (C) Decomposition

results for both groups at bin sizes of 150 and 200. The crosstalk extent of associated CN and CT is represented by the weight of the line with indicated numbers. (D) Heatmap showing the Euclidean distance between CN mod□ules constructed in the control (left) and treatment (right) groups at the indicated bin sizes, respectively. (E) Projection of CN5 on adjacent H&E staining of samples 518 (left) and 710 (right).

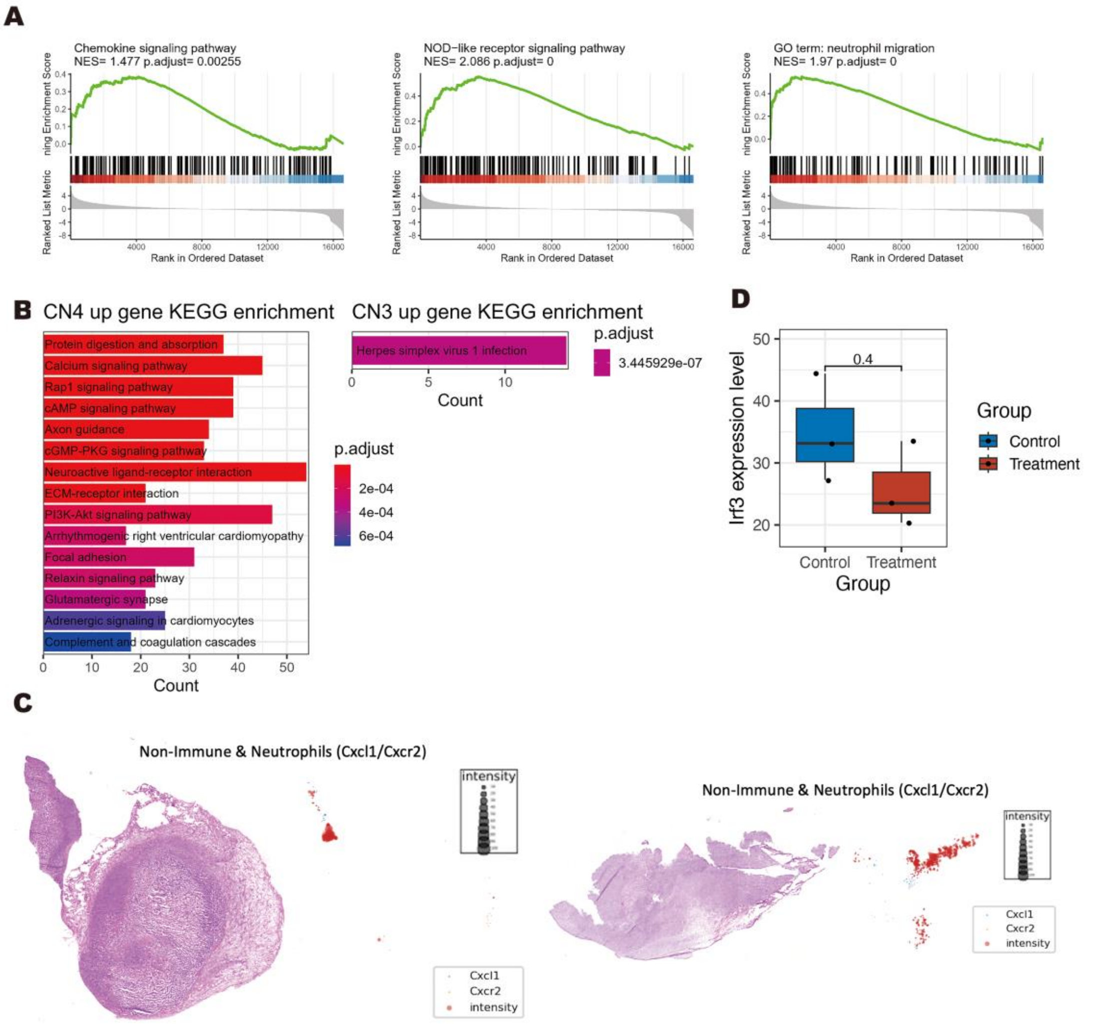

Supplementary Fig. S3. Deconvolution of CN of interest. (A) GSEA analysis of indicated signaling pathways in CN5 compared to those in counterpart CNs. (B) KEGG analysis of upregulated signaling pathways respectively in CN4 (left) and CN3 (right) compared to that in counterpart CNs. (C) In situ visualization of Cxcl1-Cxcr2 between nonimmune cells and neutrophils in 518 (left) and 710 (right) with adjacent H&E image displayed to exhibit the crosstalk coordinates. (D) Boxplot of Irf3 expression in control groups and treatment groups (P = 0.4).
