# Supplementary material for: StereoSiTE: a framework to spatially and quantitatively profile the cellular neighborhood organized iTME
Source: Gigascience. 2024 Oct 25;13:giae078. doi: 10.1093/gigascience/giae078 (PMC11503478; doi:10.1093/gigascience/giae078)
Supplement: giae078_Response_to_Reviewer_Comments_Original_Submission [file giae078_response_to_reviewer_comments_original_submission.pdf]

Author's Response To Reviewer Comments

(Note: We have prepared a Word document in response to the reviewer's suggestions, which includes replies to all comments along with figures for clarification. This document is provided in the supplementary materials for your review. Please refer to the uploaded supplementary materials for further details. Thank you.)

Dear editor and reviewers

First, we (the authors) would like to express our sincere gratitude to the editor and anonymous reviewers for their time and efforts given to our manuscript (Manuscript ID: GIGA-D-23-00276). Thanks a lot, and best regards.
Secondly, it is worth pointing out that the reviewers' comments and suggestions have really and constructively helped us improve the quality and presentation of our manuscript much further. Considering their inspiring comments and suggestions, we have duly and carefully revised the manuscript, with the main changes highlighted in red color in the revised manuscript.
Thirdly, with many thanks to the reviewers, we would like to address their comments below.

Authors Response to Comments of Editor

Comment: In addition, please register any new software application in the bio.tools and SciCrunch.org databases to receive RRID (Research Resource Identification Initiative ID) and biotoolsID identifiers and include these in your manuscript (in the availability section).
Response: Many thanks are given to the editor for this comment.
We have registered the software application on bio.tools and SciCrunch.org, respectively and added the RRID and biotoolsID to the "Code Availability" section of the revised manuscript on Page 23 (Lines 511-512).

Authors Response to Comments of Reviewer 1

Major comments:
Comment 1: The authors incorporated several open sources bioinformatics tools. However, how to ensure their combination is the optimal to the spatially resolved cell-cell communication inference performance? For example, cell2location was used to deconvolute cellular composition and construct cellular neighborhood. Why use cell2location for deconvoluting spatial transcriptomics data? Why not use the newest deconvolution algorithms, for example, SpaDecon, Celloscope, POLARIS, GraphST, SPASCER, and EnDecon? No model can adapt to all data. The authors should first verify that cell2location is the best appropriate cell type annotation tool corresponding to iTME. If not, the subsequent analyses will be not appropriate.
Response 1: Many thanks are given to the reviewer for the comment.
We have compared the performance of Cell2location with other deconvolution algorithms (Celloscope, GraphST, POLARIS) on both STARmap datasets and the stereo-seq dataset of liver cancer. Cell2location showed superior performance in the deconvolution and annotation of cell types. The results figures are presented in Supplementary Figure 1A, B. The detailed description was added to the Results and Methods section of the manuscript on Page 3 (Lines 71-76), Pages 27-28 (Lines 609-644).

Comment 2: The authors claimed that they computed the decomposition losses of different combinations of the number of CN modules and CT modules. Which combinations? The authors should list them.
Response 2: We sincerely thank the reviewer for the comment.
The results of various combinations have been displayed in Supplementary Figure 2B. The figure was misreferenced in a previously submitted manuscript. We have corrected this in the revised manuscript on Page 15 (Lines 352-354).

Comment 3: When measuring spatial cell interaction intensity, the authors only simply summed up the ligand and receptor gene expression information of the sender and receiver cells. Why not consider existing

classical intercellular communication intensity methods? The authors should compare other intercellular communication intensity measurement methods. Please refer to the following two cites:
Cell-cell communication inference and analysis in the tumour microenvironments from single-cell transcriptomics: data resources and computational strategies, briefings in bioinformatics.
CellDialog: A Computational Framework for Ligand-receptor-mediated Cell-cell Communication Analysis, IEEE Journal of Biomedical and Health Informatics.
Deciphering ligand-receptor-mediated intercellular communication based on ensemble deep learning and the joint scoring strategy from single-cell transcriptomic data, Computers in Biology and Medicine.
Response 3: Thank the reviewer very much for such a comment.
On the one hand, most of the existing classical methods for assessing intercellular communication intensity have not taken spatial information into account, as they were originally designed to analyze single-cell sequencing data, like CellPhoneDB and CellCat. On the other hand, there are some newly published methods for inferring spatial intercellular interactions, such as Giotto, SpaTalk, and CellChat v2. However, these methods have not efficiently utilized spatial information. They simply set a consistent distance threshold without considering different types of interactions or compute N neighboring cells by KNN without considering actual distances. What's more, the amount of stereo-seq data is substantial, often involving millions of cells, which necessitates a significant computational workload. However, most of the published methods were implemented in R and have not been optimized for performance, resulting in the inability to analyze stereo-seq datasets. Instead of integrating gene expression with cell spatial information to infer intercellular communication, SCII optimized computational performance by utilizing matrix calculations to implement functionality. This enables it to process large amounts of data quickly. Despite the aforementioned reasons, we have systematically compared SCII with several other methods for measuring communication intensity (CellphoneDB, CellChat v2, Giotto, SpaTalk) using a small portion of our stereo-seq demo data. SCII showed superior performance compared to other methods. The results are depicted in Figures 3H, I, J. The detailed description was added to the Results and Methods section of the revised manuscript on Pages 10-11 (Lines 241-258) and Page 31 (Lines 717-731).

Comment 4: For protein-protein interaction analysis, the authors queried 628 significant up regulated genes in CN5 area of treatment samples from STRING. Can all obtained proteins be ligands or receptors? In addition, they labeled hub genes and key protein-protein interaction networks, what were these hub genes and key networks used for?
Response 4: Thanks to the reviewer for the comment.
Out of the 628 significantly upregulated genes, 505 proteins were mapped in the STRING database version 11.5. Not all the identified proteins are ligands or receptors. We queried 505 proteins from CellPhoneDB, CellChatDB, and NicheNet ligand-receptor databases, and found that 119 proteins belong to ligands or receptors. The other proteins may interact with proteins within cells that are involved in cellular pathways, such as Irf1 (Interferon regulatory factor 1), which plays a role in regulating IFN and IFN-inducible genes. We labeled the hub genes and key PPI network in Figure 6G. These hub genes and key networks were used to elucidate the dominant biological protein-protein interactions in the functional iTME unit CN5. The hub genes, including Il1b, Ccl4, Cxcl2, Cxcl1, and Il6, were the responsive downstream targets of NF-kappa B in the context of STING signaling activation, which was well validated in previous publication as follow:

37. Motwani M, Pesiridis S and Fitzgerald KA. DNA sensing by the cGAS-STING pathway in health and disease. Nat Rev Genet. 2019;20 11:657-74. doi:10.1038/s41576-019-0151-1.

Comment 5: Which ligand-receptor pairs could mediate intercellular communication within immune tumor microenvironment? Among these L-R pairs, which L-R pairs are known in existing databases and which L-R pairs are the predicted ones?
Response 5: Thanks to the reviewer for the comment.
In this study, we introduced the existing L-R database from CellChatDB as reference. It integrates signaling molecule interaction data from the KEGG Pathway database and incorporates information from recent experimental studies. Theoretically, all L-R pairs could mediate intercellular communication within the immune tumor microenvironment, and all of them are known in existing databases without any predicted

ones.

5. Jin SQ, Guerrero-Juarez CF, Zhang LH, Chang I, Ramos R, Kuan CH, et al. Inference and analysis of cell-cell communication using CellChat. Nat Commun. 2021;12 1 doi:10.1038/s41467-021-21246-9.

Comment 6: "The enrichment analysis of individual CN showed that each CN had a dominant cell type with a spatial aggregation (Fig 2F), which was increasingly obvious than that in whole slide (Fig 2E)." What's a dominant cell type? How to define it?
Response 6: We thank the reviewer for the suggestion.
The dominant cell type was determined based on the neighborhood enrichment matrix, which had the highest neighborhood enrichment score compared to the others. We can clearly identify dominant cell types in neighborhood enrichment heatmaps (Fig 2F) of each CN region. In the CN0 region, the neighborhood enrichment of Teff cells was more pronounced. In the CN1 region, the neighborhood enrichment of M1-like and M2-like cells was stronger than that of other cell types. In the CN5 region, there was a significant neighborhood enrichment of neutrophils, indicating a spatial preference for neutrophil aggregation in CN5.

Comment 7: "To reduce the variance among open-sourced L-R databases, we unified L-R database in SCII by choosing L-R dataset in CellChatDB, which assigned each L-R with an interaction distance associated classification as secreted signaling, ECM receptor and cell-cell contact." How to unify L-R database? Did it allow for user-specified LR databases and/or add user-specified LR databases?
Response 7: Many thanks are given to the reviewer for this comment.
Considering difference communication types (secreted signaling, ECM receptor and cell-cell contact) associated to different active distance, we recommend L-R dataset in CellChatDB for its clear classification (revised sentence in Pages 9 (Lines 202-205)). Also, the selection of databases is flexible and customer defined. It allows users to introduce LR databases of interest. The database file should be formatted in CSV format and contains columns 'source' and 'target', which indicate the ligand and receptor. If users want to process different types of LR with different strategies, the column 'annotation' indicating LR types is required. The SOP for "user defined database" was revised in the Methods section of the revised manuscript on Page 30 (Lines 704-710).

Comment 8: In figure 3, how to confirm which L-R pairs mediate intercellular communication?
Response 8: Many thanks are given to the reviewer for this comment.
Every intercellular communication inferred by SCII contains two values: spatial cell interaction intensity and p-value. We defined communications with p-values < 0.05 as significant and considered them as L-R pairs mediating intercellular communication. To be noted, the threshold is flexible. The user can set a threshold for intensity to filter out interactions of interest.

Comment 9: StereoSiTE is composed of multiple modules, is it scalable? Can some of these modules (such as clustering and cell type annotation) be replaced with other more powerful modules?
Response 9: Sincerely thank the reviewer for the comment.
StereoSiTE is a modular software implemented as a Python package (https://github.com/STOmics/stereosite, https://pypi.org/project/stereosite/). Each module can be called separately to analyze the data. Therefore, it is scalable; any module can be replaced with more powerful alternatives.

Comment 10: The authors claimed that "CellPhoneDB detected many false positive interactions". How to find these false positive LRIs? How to validate the LRIs be false positives? Please list the found false positive LRIs.
Response 10: We thank the reviewer for the suggestion.
We identified the intercellular interactions inferred by CellPhoneDB but not by SCII and classified them as false positive LRIs. To validate these LRIs as false positives, we calculated their median cell distance and compared it to the median cell distance of the interactions inferred by both CellPhoneDB and SCII. The results in Figure 3D indicate that false positive LRIs had a longer median cell distance compared to the others. Besides, we selected several representative false positive LRIs and showed their spatial distribution

and permutation test results in Figure 3G. They are clustered into two categories: 1) interactions that exhibit spatial co-expression between ligand and receptor but lack significance in spatial proximity with high p-values. 2) interactions show no spatial co-expression between ligands and receptors. The false positive LRIs found have been listed in Supplementary Table 1. A more detailed description can be found in the revised manuscript on Pages 9-10 (Lines 212-237).

Comment 11: In Figure 3, the authors should add comparison experiments between StereoSiTME and classical intercellular communication analysis tools.
Response 11: Thank the reviewer for such a comment.
We have compared the performance of SCII with other intercellular communication analysis tools (CellPhonDB, CellChat v2, Giotto, Spatalk). In the study, SCII demonstrated superior performance compared to other methods. The result figures are shown in Figure 3H, I, J. The detailed description was added to the Results and Methods section of the revised manuscript on Pages 10-11 (Lines 241-258) and Page 31 (Lines 717-731).

Minor Comments:
Comment 1: The text in subfigure A, B, and C in Supplementary Figure 2 is obscure. The authors should revise Supplementary Figure 2.
Response 1: We thank the reviewer for the suggestion.
We have revised Supplementary Figure 2, which has been renamed to Figure 3 as recommended. The figure can be found in the revised manuscript on Page 37.

Comment 2: In Section "Abstract", iTME should use full name when it first appears.
Response 2: We thank the reviewer for the suggestion.
We have added the full name in the section abstract of the revised manuscript on Page 1 (Line 22).

Comment 3: Which cites of "13 Li, M. et al. (2023)." is in the reference list?
Response 3: We thank the reviewer for the suggestion.
We have corrected the cite of "13 Li, M. et al. (2023)." in the reference list, which is on Page 39 (Lines 866-868).

22. Li M, Liu H, Li M, Fang S, Kang Q, Zhang J, et al. StereoCell enables high accuracy single cell segmentation for spatial transcriptomic dataset. bioRxiv. 2023:2023.02.28.530414. doi:10.1101/2023.02.28.530414.

Authors Response to Comments of Reviewer 2
Comment: Although the authors performed comprehensive works to demonstrate the potential applications of StereoSiTE. This reviewer has strong concerns about the potential novelty and effectiveness of StereoSiTE over existing methods. The CN results were not mindful of the spatial information, and the labeled cellular neighborhood (CN) may mislead users. Additionally, although the L-R pair could be categorized into three classifications based on interaction distance, the SCII only uses different radius to infer cell communication without employing a different strategy for predicting interactions in distinct L-R pairs.
Response: Sincerely thank the reviewer for the comment.

The concept of cellular neighborhood has been first proposed to analyze multi-fluorescence data. This is a well-recognized concept to decode the TME with cellular identification. The design of Stereosite framework to address the question of “how iTME respond and function under stimulation” with stereo-seq data. Therefore, the composition in iTME community is critical for downstream analysis. We believe the novelties of our work are: 1. A self-developing algorithm called SCII to quantitively calculate the interaction intensity by introducing spatial information; 2. Providing flexible and user friendly framework to analysis spatially resolved data at single cell resolution.

Although CN does not utilize spatial information, it is capable of identifying tissue domains. CN showed good performance compared to other tissue domain division methods (BANKSY, Giotto HMRF). The results have been added to the Results section of the revised manuscript on Page 7 (Lines 170-177). In the current framework, the CN is designed to identify functional domain. Afterwards, cellular interaction within CN of interest will be decoded by SCII with full consideration of spatial information. The identification of domain should correspond to the research design; therefore, our framework allows replacement of CN module by other method. Meanwhile, we are planning to integrate spatial information into the CN analysis to take spatial pattern into consideration.
Considering the comment regarding the strategy for predicting interactions in distinct L-R pairs, we have updated the SCII function of the StereoSiTE software. Different strategies for predicting interactions in distinct L-R pair types have been implemented. Users can specify different radius thresholds for distinct L-R types using parameters, for example: {'Secreted Signaling': 100, 'ECM-Receptor': 100, 'Cell-Cell Contact': 30}. The new version of StereoSiTE has been updated on GitHub and PyPI (https://github.com/STOmics/stereosite, https://pypi.org/project/stereosite/). At the same time, we have updated the Results section of the revised manuscript on Pages 9-10 (Lines 202-240), and Figure 3A-G.

Comments:
Comment 1: The authors fail to show the novelty and advantages of CN compared to other methods, such as DeepST, which integrates gene expression, spatial location and image information. The authors should provide the comparison with the several recent strategies that consider the effect of local niches including BANKSY, stLearn, Giott, and DeepST.
Response 1: Sincerely thank the reviewer for the comment.
We have compared CN with other methods. The results demonstrate that the CN method exhibits nearly consistent performance with BANSKY, while outperforming Giotto HMRF on the benchmark dataset STARmap. The detailed description has been added to the Results section of the revised manuscript on Page 7 (Lines 170-177). Because stLearn and DeepST require H&E images, which were not included in our dataset, we have not been able to conduct a comparison with them.

Comment 2: The authors should compare SCII with additional methods such as CellPhoneDB v3 and Cellchat v2, demonstrating its superior performance.
Response 2: Thank the reviewer for such a comment.
We have compared SCII with additional methods (CellPhonDB v4, CellCgat v2, Giotto, Spatalk) using part of our stereo-seq demo data. SCII showed superior performance compared to other methods. The results are depicted in Figure 3H, I, J. The detailed description was added to the Results and Methods section of the revised manuscript on Pages 10-11 (Lines 241-258) and Page 31 (Lines 717-731).

Comment 3: The method used for cell segmentation should offer more comprehensive information rather than solely citing "Li, M. et al. (2023)".
Response 3: Many thanks are given to the reviewer for raising the comment.
We have added comprehensive information about the cell segmentation method in the method section of the revised manuscript on Page 26 (Lines 573-581).

Comment 4: Format of the paper. The alignment inconsistency within the manuscript—with some paragraphs centered and others justified—should be corrected for uniformity.
Response 4: Sincerely thank the reviewer for the comment.
We have corrected the format of the revised manuscript.

Comment 5: The figures and manuscript containing 'Teff' and 'M2-like' cell types should provide a legend explaining the abbreviations for clarity.
Response 5: Sincerely thank the reviewer for the comment.
We have provided a legend explaining the abbreviations for clarity in the legend of figure 2 on Page 8

(Lines 191-193).

Comment 6: The font size of the labels in Figures 5E-F is insufficient for easy reading and should be enlarged.
Response 6: Sincerely thank the reviewer for the comment.
We readjusted the font size of Figures 5E-F for easy reading. The figure can be found in the revised manuscript on Page 17.

Authors Response to Comments of Reviewer 3
Comment 1: The paper's objective is commendable, and the overall organization of the content, along with the obtained results, holds great promise. Nevertheless, certain aspects need to be addressed. The proposed approach's novelty is significantly anchored in the SCII software. However, the paper has notable drawbacks. It falls short in providing a theoretical and scientific comparison with other similar tools. Moreover, the comparison includes systems that do not incorporate spatial considerations, posing a limitation in assessing the method's uniqueness in a broader context.
Response 1: Sincerely thank the reviewer for the comment.
We have systematically compared SCII with several other intercellular communication measurement methods (CellPhoneDB v4, CellChat v2, Giotto, Spatalk). Considering the limited computational efficiency, we selected part of the demo data containing 11,214 cells for comparison. We calculated the co-expression percentage of interactions between sender and receiver cells inferred by distinct methods. We hypothesized that a higher co-expression percentage indicates more reliable inference. Interactions inferred by SCII showed the highest level of co-expression, indicating the superior performance of SCII in measuring spatial cell-cell communication. The results have been updated in Figures 3H, I, J, and on Pages 10-11 (Lines 241-258). The method has been added on Page 31 (Lines 717-731).

Comment 2: Give more details on which systems are you referring here: "To improve accuracy, we recommended using spatially resolved data at single cell resolution". Please provide your insights on the rationale for employing or abstaining from downstream analysis to comprehend the spatial distribution of gene expression in tissue, as https://doi.org/10.1093/gigascience/giac075 and https://doi.org/10.1038/s41467-023-36796-3. Additionally, consider discussing how this is associated with the prediction, validation of the functional enrichment or on step: Clustering bins into different cellular neighborhoods based on their cellular composition.
Response 2: Sincerely thank the reviewer for the comment.
We have given more details about the single cell resolution systems mentioned in the paper in the Results section of the revised manuscript on Page 4 (Lines 93-95).
Compared with methods like GraphST and Stardust, which achieve tissue domain division by utilizing gene expression matrix and genes' spatial distribution, tissue domain division based on cellular composition (CN) offers a more detailed and accurate understanding of tissue biology, cellular function, and disease mechanisms. 1. It allows for a more accurate identification of specific cell types present within a tissue. This can help in understanding the functional roles and characteristics of individual cell types within a tissue, providing a more detailed insight into tissue function. 2. By focusing on cellular composition, tissue domain division allows for the analysis of individual cell types within a tissue. This can help uncover specific cellular functions, interactions, and pathways that may be relevant to tissue function or disease processes. 3. Tissue domain division based on cellular composition can provide insights into disease mechanisms at the cellular level. By studying specific cell types implicated in a disease, researchers can better understand the underlying cellular processes and develop targeted therapies.
Tissue domain division by CN can help us focus on specific regions with an enrichment of the cell types of interest. As described in our manuscript, we next analyzed the intercellular communications in the specific CN region and found some biologically meaningful interactions. Theoretically, after the interaction between the ligand expressed by sender cells and the receptor expressed by receiver cells, downstream transcriptome factors and corresponding pathways in receiver cells will be activated. Therefore, we analyzed the transcription factor activities and pathway enrichment in the specific CN region.

Finally, we (the authors) would like to express thanks again sincerely to the editor and anonymous reviewers for their time and efforts spent in handing the manuscript, as well as providing us many constructive comments for improving further the presentation and quality of this manuscript.

Sincerely,

Xing LIU

(On behalf of all authors)

11 Apr 2024
