# Supplementary material for: StereoSiTE: a framework to spatially and quantitatively profile the cellular neighborhood organized iTME
Source: Gigascience. 2024 Oct 25;13:giae078. doi: 10.1093/gigascience/giae078 (PMC11503478; doi:10.1093/gigascience/giae078)
Supplement: giae078_Response_to_Reviewer_Comments_Revision_1 [file giae078_response_to_reviewer_comments_revision_1.pdf]

Author's Response To Reviewer Comments

Dear editor and reviewers

Firstly, we (the authors) wish to express our sincere appreciation to the editor and anonymous reviewers for dedicating their time and efforts to evaluating our revised manuscript (Manuscript ID: GIGA-D-23-00276R1). Thank you, very much, and best regards.
Secondly, it is noteworthy that the feedback and recommendations provided by the reviewers have significantly contributed to enhancing the quality and presentation of our manuscript. The insightful comments and suggestions have guided us in making thorough revisions to the manuscript.
Thirdly, we express our gratitude to the reviewers and aim to address their comments as outlined below.

Authors Response to Comments of Editor

Comment: Specifically, to substantiate the preference for CN over BANKSY, the authors are encouraged to provide evidence of its user-friendly interface, shorter run time, or lower memory usage. Please also consider getting this paper copy edited by a native English speaker to improve the language.
Response: Many thanks are given to the editor for this comment.
We conducted a comparison of the runtimes of CN and BANSKY for increasing cell numbers, up to 2 million cells. CN demonstrated the ability to manage large-scale datasets more efficiently than BANSKY, resulting in reduced processing time and improved user-friendliness. This finding has been incorporated into the revised manuscript on page 8 (Lines 210-212).
We have improved the linguistic quality of the entire manuscript.

Authors Response to Comments of Reviewer 1

Major comments:
Comment 1: The authors have compared the performance of Cell2location with other cell type identification methods, Celloscope[10], GraphST[11], and POLARIS[12] on both STARmap and stereo-seq dataset of liver cancer. How about its performance on other unlabeled datasets? Please compare it with "STGNNks: Identifying cell types in spatial transcriptomics data based on graph neural network, denoising auto-encoder, and -sums clustering".
Response 1: Many thanks are given to the reviewer for the comment.
Instead of cell type identification method, the study "Identifying cell types in spatial transcriptomics data based on graph neural network, denoising auto-encoder, and -sums clustering” introduced a spatial clustering approach. Consequently, it is not able to directly comparable to other methods for cell type identification.

Comment 2: Cell-cell communication is usually mediated by LRIs. The construction of high-quality LRI databases is very important to cell-cell communication. The authors should introduce these LRI data resources and potential LRI prediction methods and cite them, for example, PMID: 37976192, 37364528, 38367445.
Response 2: We sincerely thank the reviewer for the comment.
We have introduced the utilized LRI data resources and potential LRI prediction methods and cited them in the revised manuscript on Page 3 (Lines 83-87).

Comment 3: In Figure 4B, 4C, 4D, and 4F, Figure 5A and 5B, Figure 6B and 6C, the fonts are too small. Please enlarge the fonts.
Response 3: Thank the reviewer very much for such a comment.
We have enlarged the fonts in these figures.

Comment 4: The organization and structure of this manuscript must be carefully revised. For example, The structure in Discussion is obscure. In the first paragraph in this section, the authors have introduced their proposed method, next, they described it in detail. But the third paragraph elucidated the reason why to develop this reason.

In addition, "Figure 3 highlights that the analysis without distance threshold may lead to false positive results, and SCII showed more superior performance than other methods." why to Figure 3? Did not the other results support their conclusion?
The final paragraph in Discussion introduced their method again.
It HAS NO logic.
Response 4: Thanks to the reviewer for the comment.
We have carefully revised the organization and structure of the manuscript.
The structure of the Discussion section has been reorganized. The first paragraph introduces StereoSiTE, followed by a description of the two key functions in the analytical framework: CN and SCII. The third paragraph presents the application results of StereoSiTE, while the final paragraph discusses the limitations and potential resolutions. This reorganization spans pages 22-24 (Lines 478-534).
The content about the performance of SCII is illustrated in Figure 3, which includes a comparison between SCII and methods that do not take physical distance into account, as well as other methods that consider spatial information. It is deemed appropriate to present these results in Figure 3.

Comment 5: Where is the conclusion of this manuscript?
Response 5: Thanks to the reviewer for the reasonable suggestion.
We have made revisions to the conclusion presented in the Abstract on page 2 (Lines 38-42).

Comment 6: The authors should analyze the limitations of this work for further work in the future.
Response 6: We thank the reviewer for the suggestion.
The limitations of the current study and potential avenues for future research have been examined in the concluding section of the Discussion on page 24 (Lines 525-534).

Comment 7: English is VERY POOR. This manuscript must be carefully revised. For example, "prove that spatial proximity is a must to guarantee an effective investigation.", is a must to do?
Response 7: Many thanks are given to the reviewer for this comment.
The manuscript has been carefully revised.

Authors Response to Comments of Reviewer 2

Comments:
Comment 1: Notably, the SCII component of the framework combines spatial information and expression patterns to infer that cell-cell communication can limit reachable interactions, thereby reducing false positive interactions. The authors have also employed distinct strategies to predict different types of L-R pairs with varying interaction distances, encompassing secreted signaling, ECM-receptor, and cell-cell contact. In the case of secreted type L-R pairs, SCII enables the specification of varying radius thresholds to infer spatial cell communication. However, it is recommended that the authors consider the exponential decay of expression values, particularly when the radius exceeds 100 μm.
Response 1: Sincerely thank the reviewer for the comment.
We have updated the SCII function in StereoSiTE by introducing the functionality to compute the spatial cell interaction intensity while considering the exponential decay of expression values of ligand genes using the formula: C=C0*e^(-k*d). Users are now empowered to specify the attenuation constant 'k' in the formula by defining the new parameter 'distance_coefficient'. Considering ligand-receptor type of Cell-Cell Contact does not involve molecular secretion, and the LR type of ECM-Receptor requires a distinct attenuation constant compared to Secreted Signaling, StereoSiTE enables users to assign different values to the distance_coefficient parameter for various LR types.
Based on the relevant background information regarding cell-cell interaction distances that have been reviewed[1, 2], it is evident that stable interaction distances vary depending on the cell type, and the diffusion coefficient of ligand molecules is associated with their molecular size. It is noteworthy that there is no universally accepted formula or coefficient for precisely modeling molecular diffusion. Therefore, the decision to consider the decay of expression values of ligand genes was deemed risky, and the potential enhancement in accuracy in computing the spatial cell interaction intensity through the introduction of an exponential decay formula remains uncertain.

Comment 2: The response also outlines the authors' claim that CN exhibits good performance compared to

other tissue domain division methods (BANKSY and Giotto HMRF). However, upon reviewing the performance comparison results, it becomes apparent that BANKSY outperforms the other methods, although the CN method shows nearly consistent performance with BANKSY on the benchmark dataset STARmap. To substantiate the preference for CN over BANKSY, the authors are encouraged to provide evidence of its user-friendly interface, shorter run time, or lower memory usage.
Response 2: Thank the reviewer for such a comment.
We have conducted a comparison of the runtimes of CN and BANSKY as the cell number increases, demonstrating the improved user-friendliness of CN. This analysis can be found on Page 8 (Lines 210-212).

Detailed information is supplied within the response letter in attachment.
We (the authors) wish to express sincere gratitude to the editor and anonymous reviewers for dedicating their time and effort to reviewing the response letter and manuscript. Their valuable feedback has been instrumental in enhancing the presentation and quality of this manuscript.
