# Supplementary material for: StereoSiTE: a framework to spatially and quantitatively profile the cellular neighborhood organized iTME
Source: Gigascience. 2024 Oct 25;13:giae078. doi: 10.1093/gigascience/giae078 (PMC11503478; doi:10.1093/gigascience/giae078)
Supplement: giae078_Reviewer_1_Report_Original_Submission — Lihong Peng -- 12/23/2023 Reviewed [file giae078_reviewer_1_report_original_submission.pdf]

Reviewer Report

Title: StereoSiTE: A framework to spatially and quantitatively profile the cellular neighborhood organized iTME

Version: Original Submission **Date:** 12/23/2023

Reviewer name: Lihong Peng

Reviewer Comments to Author:

In this manuscript, the authors developed a computational framework named StereoSiTE to spatially and quantitatively profile the cellular neighborhood organized iTME by incorporating open source bioinformatics tools with their self-proposed algorithm named SCII. This study is very meaningful. However, it remains several problems.

Major comments:

1. The authors incorporated several open sources bioinformatics tools. However, how to ensure their combination is the optimal to the spatially resolved cell-cell communication inference performance? For example, cell2location was used to deconvolute cellular composition and construct cellular neighborhood. Why to use cell2location for deconvoluting spatial transcriptomics data? why not use the newest deconvolution algorithms, for example, SpaDecon, Celloscope, POLARIS, GraphST, SPASCER, and EnDecon? No model can adapt to all data. The authors should first verify that cell2location is the best appropriate cell type annotation tool corresponding to iTME. If not, the subsequent analyses will be not appropriate.
2. The authors claimed that they computed the decomposition losses of different combinations of the number of CN modules and CT modules. Which combinations? The authors should list them.
3. When measuring spatial cell interaction intensity, the authors only simply summed up the ligand and receptor gene expression information of the sender and receiver cells. Why not consider existing classical intercellular communication intensity methods? The authors should compare other intercellular communication intensity measurement methods. Please refer to the following two cites:

Cell-cell communication inference and analysis in the tumour microenvironments from single-cell transcriptomics: data resources and computational strategies, briefings in bioinformatics.

CellDialog: A Computational Framework for Ligand-receptor-mediated Cell-cell Communication Analysis, IEEE Journal of Biomedical and Health Informatics.

Deciphering ligand-receptor-mediated intercellular communication based on ensemble deep learning and the joint scoring strategy from single-cell transcriptomic data, Computers in Biology and Medicine.

4. For protein-protein interaction analysis, the authors queried 628 significant up regulated genes in CN5 area of treatment samples from STRING. Can all obtained proteins be ligands or receptors? In addition, they labeled hub genes and key protein-protein interaction networks, what were these hub genes and key networks used for?
5. Which ligand-receptor pairs could mediate intercellular communication within immune tumor microenvironment? Among these L-R pairs, which L-R pairs are known in existing databases and which L-R pairs are the predicted ones?

6. "The enrichment analysis of individual CN showed that each CN had a dominant cell type with a spatial aggregation (Fig 2F), which was increasingly obvious than that in whole slide (Fig 2E)." What's a dominant cell type? How to define it?
7. "To reduce the variance among open-sourced L-R databases, we unified L-R database in SCII by choosing L-R dataset in CellChatDB, which assigned each L-R with an interaction distance associated classification as secreted signaling, ECM receptor and cell-cell contact." How to unify L-R database? Did it allow for user-specified LR databases and/or add user-specified LR databases?
8. In figure 3, how to confirm which L-R pairs mediate intercellular communication?
9. StereoSiTE is composed of multiple modules, is it scalable? Can some of these modules (such as clustering and cell type annotation) be replaced with other more powerful modules?
10. The authors claimed that "CellPhoneDB detected many false positive interactions". How to find these false positive LRIs? How to validate the LRIs be false positives? Please list the found false positive LRIs.
11. In Figure 3, the authors should add comparison experiments between StereoSiTME and classical intercellular communication analysis tools.
Minor comments:
1. The text in subfigure A, B, and C in Supplementary Figure 2 is obscure. The authors should revise Supplementary Figure 2.
2. In Section "Abstract", iTME should use full name when it first appears.
3. Which cites of "13 Li, M. et al. (2023)." is in the reference list?

Methods

Are the methods appropriate to the aims of the study, are they well described, and are necessary controls included? Choose an item.

Conclusions

Are the conclusions adequately supported by the data shown? Choose an item.

Reporting Standards

Does the manuscript adhere to the journal's guidelines on [minimum standards of reporting?]() Choose an item.

Choose an item.

Statistics

Are you able to assess all statistics in the manuscript, including the appropriateness of statistical tests used? Choose an item.

Quality of Written English

Please indicate the quality of language in the manuscript: Choose an item.

Declaration of Competing Interests

Please complete a declaration of competing interests, considering the following questions:

- Have you in the past five years received reimbursements, fees, funding, or salary from an organisation that may in any way gain or lose financially from the publication of this manuscript, either now or in the future?
- Do you hold any stocks or shares in an organisation that may in any way gain or lose financially from the publication of this manuscript, either now or in the future?
- Do you hold or are you currently applying for any patents relating to the content of the manuscript?
- Have you received reimbursements, fees, funding, or salary from an organization that holds or has applied for patents relating to the content of the manuscript?
- Do you have any other financial competing interests?
- Do you have any non-financial competing interests in relation to this paper?

If you can answer no to all of the above, write 'I declare that I have no competing interests' below. If your reply is yes to any, please give details below.

I declare that I have no competing interests.

I agree to the open peer review policy of the journal. I understand that my name will be included on my report to the authors and, if the manuscript is accepted for publication, my named report including any attachments I upload will be posted on the website along with the authors' responses. I agree for my report to be made available under an Open Access Creative Commons CC-BY license (http://creativecommons.org/licenses/by/4.0/). I understand that any comments which I do not wish to be included in my named report can be included as confidential comments to the editors, which will not be published.

Choose an item.

To further support our reviewers, we have joined with Publons, where you can gain additional credit to further highlight your hard work (see: https://publons.com/journal/530/gigascience). On publication of this paper, your review will be automatically added to Publons, you can then choose whether or not to claim your Publons credit. I understand this statement.

Yes Choose an item.
