# Supplementary material for: StereoSiTE: a framework to spatially and quantitatively profile the cellular neighborhood organized iTME
Source: Gigascience. 2024 Oct 25;13:giae078. doi: 10.1093/gigascience/giae078 (PMC11503478; doi:10.1093/gigascience/giae078)
Supplement: giae078_Reviewer_1_Report_Revision_1 — Lihong Peng -- 12/23/2023 Reviewed [file giae078_reviewer_1_report_revision_1.pdf]

Reviewer Report

Title: StereoSiTE: A framework to spatially and quantitatively profile the cellular neighborhood organized iTME

Version: Revision 1 **Date:** 12/23/2023

Reviewer name: Lihong Peng

Reviewer Comments to Author:

In the revised manuscript, the authors conducted lots of revisions. However, it still remains many problems to solve:
1. The authors have compared the performance of Cell2location with other cell type identification methods, Celloscope[10], GraphST[11], and POLARIS[12] on on both STARmap and stereo-seq dataset of liver cancer. How about its performance on other unlabeled datasets? Please compare it with "STGNNks: Identifying cell types in spatial transcriptomics data based on graph neural network, denoising auto-encoder, and ð‘˜-sums clustering".
2. Cell-cell communication is usually mediated by LRIs. The construction of high-quality LRI databases is very important to cell-cell communication. The authors should introduce these LRI data resources and potential LRI prediction methods and cite them, for example, PMID: 37976192, 37364528, 38367445.
3. In Figure 4B, 4C, 4D, and 4F, Figure 5A and 5B, Figure 6B and 6C, the fonts are too small. Please enlarge the fonts.
4. The organization and structure of this manuscript must be carefully revised. For example,
The structure in Discussion is obscure. In the first paragraph in this section, the authors have introduced their proposed method, next, they described it in details. But the third paragraph elucidated the reason why to develop this reason.
In addition, "Figure 3 highlights that the analysis without distance threshold may lead to false positive results, and SCII showed more superior performance than other methods." why to Figure 3? Did not the other results support their conclusion?
The final paragraph in Discussion introduced their method again.
It HAS NO logic.
5. Where is the conclusion of this manuscript?
6. The authors should analyze the limitations of this work for further work in the future.
7. English is VERY POOR. This manuscript must be carefully revised. For example,
"prove that spatial proximity is a must to guarantee an effective investigation.", is a must to do?

Methods

Are the methods appropriate to the aims of the study, are they well described, and are necessary controls included? Choose an item.

Conclusions

Are the conclusions adequately supported by the data shown? Choose an item.

Reporting Standards

Does the manuscript adhere to the journal's guidelines on minimum standards of reporting? Choose an item.

Choose an item.

Statistics

Are you able to assess all statistics in the manuscript, including the appropriateness of statistical tests used? Choose an item.

Quality of Written English

Please indicate the quality of language in the manuscript: Choose an item.

Declaration of Competing Interests

Please complete a declaration of competing interests, considering the following questions:

- Have you in the past five years received reimbursements, fees, funding, or salary from an organisation that may in any way gain or lose financially from the publication of this manuscript, either now or in the future?
- Do you hold any stocks or shares in an organisation that may in any way gain or lose financially from the publication of this manuscript, either now or in the future?
- Do you hold or are you currently applying for any patents relating to the content of the manuscript?
- Have you received reimbursements, fees, funding, or salary from an organization that holds or has applied for patents relating to the content of the manuscript?
- Do you have any other financial competing interests?
- Do you have any non-financial competing interests in relation to this paper?

If you can answer no to all of the above, write 'I declare that I have no competing interests' below. If your reply is yes to any, please give details below.

I declare that I have no competing interests.

I agree to the open peer review policy of the journal. I understand that my name will be included on my report to the authors and, if the manuscript is accepted for publication, my named report including any attachments I upload will be posted on the website along with the authors' responses. I agree for my report to be made available under an Open Access Creative Commons CC-BY license (http://creativecommons.org/licenses/by/4.0/). I understand that any comments which I do not wish to

be included in my named report can be included as confidential comments to the editors, which will not be published.

Choose an item.

To further support our reviewers, we have joined with Publons, where you can gain additional credit to further highlight your hard work (see: https://publons.com/journal/530/gigascience). On publication of this paper, your review will be automatically added to Publons, you can then choose whether or not to claim your Publons credit. I understand this statement.

Yes Choose an item.
