# Supplementary material for: StereoSiTE: a framework to spatially and quantitatively profile the cellular neighborhood organized iTME
Source: Gigascience. 2024 Oct 25;13:giae078. doi: 10.1093/gigascience/giae078 (PMC11503478; doi:10.1093/gigascience/giae078)
Supplement: giae078_Reviewer_2_Report_Original_Submission — Chenfei Wang -- 12/25/2023 Reviewed [file giae078_reviewer_2_report_original_submission.pdf]

Reviewer Report

Title: StereoSiTE: A framework to spatially and quantitatively profile the cellular neighborhood organized iTME

Version: Original Submission **Date:** 12/25/2023

Reviewer name: Chenfei Wang

Reviewer Comments to Author:

In this manuscript, Xin. et al. provided a framework called StereoSiTE that incorporated the established methodologies with their developed algorithm to defined cellular neighborhood (CN) organized immune tumor microenvironment (iTME) based on cellular compositions, and to dissected the spatial cell interaction intensity (SCII) in spatial transcriptomics (ST). StereoSiTE has the following improvements compared to existing methods. First, SCII detects cell-cell communication using both cell space nearest neighbor graph and targeted L-R expression. Second, SCII taken the interaction distance account for different interaction classification such as secreted signaling, ECM receptor and cell-cell contact. Finally, StereoSiTE could avoided to detected the false positive interactions caused by limited reachable interaction.

Although the authors performed comprehensive works to demonstrate the potential applications of StereoSiTE. This reviewer has strong concerns about the potential novelty and effectiveness of StereoSiTE over existing methods. The CN results were not mindful of the spatial information, and the labeled cellular neighborhood (CN) may mislead users. Additionally, although the L-R pair could be categorized into three classifications based on interaction distance, the SCII only uses different radius to infer cell communication without employing a different strategy for predicting interactions in distinct L-R pairs.

I have the following detailed comments.

Commentsï¼š

1. The authors fail to show the novelty and advantages of CN compared to other methods, such as DeepST, which integrates gene expression, spatial location and image information. The authors should provide the comparison with the several recent strategies that consider the effect of local niches including BANKSY, stLearn, Giott, and DeepST.
2. The authors should compare SCII with additional methods such as CellPhoneDB v3 and Cellchat v2, demonstrating its superior performance.
3. The method used for cell segmentation should offer more comprehensive information rather than solely citing "Li, M. et al. (2023)".
4. Format of the paper. The alignment inconsistency within the manuscriptâ€"with some paragraphs centered and others justifiedâ€"should be corrected for uniformity.
5. The figures and manuscript containing 'Teff' and 'M2-like' cell types should provide a legend explaining the abbreviations for clarity.
6. The font size of the labels in Figures 5E-F is insufficient for easy reading and should be enlarged.

Methods

Are the methods appropriate to the aims of the study, are they well described, and are necessary controls included? Choose an item.

Conclusions

Are the conclusions adequately supported by the data shown? Choose an item.

Reporting Standards

Does the manuscript adhere to the journal's guidelines on [minimum standards of reporting?]() Choose an item.

Choose an item.

Statistics

Are you able to assess all statistics in the manuscript, including the appropriateness of statistical tests used? Choose an item.

Quality of Written English

Please indicate the quality of language in the manuscript: Choose an item.

Declaration of Competing Interests

Please complete a declaration of competing interests, considering the following questions:

- Have you in the past five years received reimbursements, fees, funding, or salary from an organisation that may in any way gain or lose financially from the publication of this manuscript, either now or in the future?
- Do you hold any stocks or shares in an organisation that may in any way gain or lose financially from the publication of this manuscript, either now or in the future?
- Do you hold or are you currently applying for any patents relating to the content of the manuscript?
- Have you received reimbursements, fees, funding, or salary from an organization that holds or has applied for patents relating to the content of the manuscript?
- Do you have any other financial competing interests?
- Do you have any non-financial competing interests in relation to this paper?

If you can answer no to all of the above, write 'I declare that I have no competing interests' below. If your reply is yes to any, please give details below.

I declare that I have no competing interests.

I agree to the open peer review policy of the journal. I understand that my name will be included on my report to the authors and, if the manuscript is accepted for publication, my named report including any attachments I upload will be posted on the website along with the authors' responses. I agree for my report to be made available under an Open Access Creative Commons CC-BY license (http://creativecommons.org/licenses/by/4.0/). I understand that any comments which I do not wish to be included in my named report can be included as confidential comments to the editors, which will not be published.

Choose an item.

To further support our reviewers, we have joined with Publons, where you can gain additional credit to further highlight your hard work (see: https://publons.com/journal/530/gigascience). On publication of this paper, your review will be automatically added to Publons, you can then choose whether or not to claim your Publons credit. I understand this statement.

Yes Choose an item.
