# Supplementary material for: StereoSiTE: a framework to spatially and quantitatively profile the cellular neighborhood organized iTME
Source: Gigascience. 2024 Oct 25;13:giae078. doi: 10.1093/gigascience/giae078 (PMC11503478; doi:10.1093/gigascience/giae078)
Supplement: giae078_Reviewer_2_Report_Revision_1 — Chenfei Wang -- 5/4/2024 Reviewed [file giae078_reviewer_2_report_revision_1.pdf]

Reviewer Report

Title: StereoSiTE: A framework to spatially and quantitatively profile the cellular neighborhood organized iTME

Version: Revision 1 **Date:** 5/4/2024

Reviewer name: Chenfei Wang

Reviewer Comments to Author:

In the response letter, the author emphasizes the novelties of the StereoSiTE framework and demonstrates how the StereoSiTE software was specifically designed to address the question of "how iTME responds and functions under stimulation" using stereo-seq data. The author highlights notable enhancements to the self-development algorithm, including CN and SCII. The CN algorithm focuses on evaluating the cell composition in iTME, while SCII is designed to infer the intensity of spatial cell interactions. These advancements have been incorporated into the updated version of the manuscript. Notably, the SCII component of the framework combines spatial information and expression patterns to infer that cell-cell communication can limit reachable interactions, thereby reducing false positive interactions. The authors have also employed distinct strategies to predict different types of L-R pairs with varying interaction distances, encompassing secreted signaling, ECM-receptor, and cell-cell contact. In the case of secreted type L-R pairs, SCII enables the specification of varying radius thresholds to infer spatial cell communication. However, it is recommended that the authors consider the exponential decay of expression values, particularly when the radius exceeds 100 Î¼m.
The response also outlines the authors' claim that CN exhibits good performance compared to other tissue domain division methods (BANKSY and Giotto HMRF). However, upon reviewing the performance comparison results, it becomes apparent that BANKSY outperforms the other methods, although the CN method shows nearly consistent performance with BANKSY on the benchmark dataset STARmap. To substantiate the preference for CN over BANKSY, the authors are encouraged to provide evidence of its user-friendly interface, shorter run time, or lower memory usage.
Overall, the revisions and enhancements made to the StereoSiTE framework significantly improve its functionality and analytical capabilities. The StereoSiTE software holds great promise in providing invaluable insights and support for potential users and researchers in the field.

Methods

Are the methods appropriate to the aims of the study, are they well described, and are necessary controls included? Choose an item.

Conclusions

Are the conclusions adequately supported by the data shown? Choose an item.

Reporting Standards

Does the manuscript adhere to the journal's guidelines on [minimum standards of reporting?]() Choose an item.

Choose an item.

Statistics

Are you able to assess all statistics in the manuscript, including the appropriateness of statistical tests used? Choose an item.

Quality of Written English

Please indicate the quality of language in the manuscript: Choose an item.

Declaration of Competing Interests

Please complete a declaration of competing interests, considering the following questions:

- Have you in the past five years received reimbursements, fees, funding, or salary from an organisation that may in any way gain or lose financially from the publication of this manuscript, either now or in the future?
- Do you hold any stocks or shares in an organisation that may in any way gain or lose financially from the publication of this manuscript, either now or in the future?
- Do you hold or are you currently applying for any patents relating to the content of the manuscript?
- Have you received reimbursements, fees, funding, or salary from an organization that holds or has applied for patents relating to the content of the manuscript?
- Do you have any other financial competing interests?
- Do you have any non-financial competing interests in relation to this paper?

If you can answer no to all of the above, write 'I declare that I have no competing interests' below. If your reply is yes to any, please give details below.

I declare that I have no competing interests

I agree to the open peer review policy of the journal. I understand that my name will be included on my report to the authors and, if the manuscript is accepted for publication, my named report including any attachments I upload will be posted on the website along with the authors' responses. I agree for my report to be made available under an Open Access Creative Commons CC-BY license (http://creativecommons.org/licenses/by/4.0/). I understand that any comments which I do not wish to be included in my named report can be included as confidential comments to the editors, which will not be published.

Choose an item.

To further support our reviewers, we have joined with Publons, where you can gain additional credit to further highlight your hard work (see: https://publons.com/journal/530/gigascience). On publication of this paper, your review will be automatically added to Publons, you can then choose whether or not to claim your Publons credit. I understand this statement.

Yes Choose an item.
