# Supplementary material for: StereoSiTE: a framework to spatially and quantitatively profile the cellular neighborhood organized iTME
Source: Gigascience. 2024 Oct 25;13:giae078. doi: 10.1093/gigascience/giae078 (PMC11503478; doi:10.1093/gigascience/giae078)
Supplement: giae078_Reviewer_3_Report_Original_Submission — Rosalba Giugno -- 1/12/2024 Reviewed [file giae078_reviewer_3_report_original_submission.pdf]

Reviewer Report

Title: StereoSiTE: A framework to spatially and quantitatively profile the cellular neighborhood organized iTME

Version: Original Submission **Date:** 1/12/2024

Reviewer name: Rosalba Giugno

Reviewer Comments to Author:

Authors introduce StereoSiTE, which integrates open-source bioinformatics tools with the self-developed algorithm SCII. The aim is to dissect a cellular neighborhood (CN) organized iTME based on cellular compositions and accurately infer functional cell-cell communications with quantitatively defined interaction intensity in ST data.
The paper's objective is commendable, and the overall organization of the content, along with the obtained results, holds great promise. Nevertheless, certain aspects need to be addressed. The proposed approach's novelty is significantly anchored in the SCII software. However, the paper has notable drawbacks. It falls short in providing a theoretical and scientific comparison with other similar tools. Moreover, the comparison includes systems that do not incorporate spatial considerations, posing a limitation in assessing the method's uniqueness in a broader context.
Give more details on which systems are you referring here: "To improve accuracy, we recommended using spatially resolved data at single cell resolution". Please provide your insights on the rationale for employing or abstaining from downstream analysis to comprehend the spatial distribution of gene expression in tissue, as https://doi.org/10.1093/gigascience/giac075 and https://doi.org/10.1038/s41467-023-36796-3. Additionally, consider discussing how this is associated with the prediction, validation of the functional enrichment or on step: Clustering bins into different cellular neighborhoods based on their cellular composition.

Methods

Are the methods appropriate to the aims of the study, are they well described, and are necessary controls included? Choose an item.

Conclusions

Are the conclusions adequately supported by the data shown? Choose an item.

Reporting Standards

Does the manuscript adhere to the journal's guidelines on minimum standards of reporting? Choose an item.

Choose an item.

Statistics

Are you able to assess all statistics in the manuscript, including the appropriateness of statistical tests used? Choose an item.

Quality of Written English

Please indicate the quality of language in the manuscript: Choose an item.

Declaration of Competing Interests

Please complete a declaration of competing interests, considering the following questions:

- Have you in the past five years received reimbursements, fees, funding, or salary from an organisation that may in any way gain or lose financially from the publication of this manuscript, either now or in the future?
- Do you hold any stocks or shares in an organisation that may in any way gain or lose financially from the publication of this manuscript, either now or in the future?
- Do you hold or are you currently applying for any patents relating to the content of the manuscript?
- Have you received reimbursements, fees, funding, or salary from an organization that holds or has applied for patents relating to the content of the manuscript?
- Do you have any other financial competing interests?
- Do you have any non-financial competing interests in relation to this paper?

If you can answer no to all of the above, write 'I declare that I have no competing interests' below. If your reply is yes to any, please give details below.

no

I agree to the open peer review policy of the journal. I understand that my name will be included on my report to the authors and, if the manuscript is accepted for publication, my named report including any attachments I upload will be posted on the website along with the authors' responses. I agree for my report to be made available under an Open Access Creative Commons CC-BY license (http://creativecommons.org/licenses/by/4.0/). I understand that any comments which I do not wish to be included in my named report can be included as confidential comments to the editors, which will not be published.

Choose an item.

To further support our reviewers, we have joined with Publons, where you can gain additional credit to further highlight your hard work (see: https://publons.com/journal/530/gigascience). On publication of this paper, your review will be automatically added to Publons, you can then choose whether or not to claim your Publons credit. I understand this statement.

Yes Choose an item.
